# New Derivatives of the Multi-Stage Active Malaria Box Compound MMV030666 and Their Antiplasmodial Potencies

**DOI:** 10.3390/ph15121503

**Published:** 2022-12-02

**Authors:** Theresa Hermann, Robin Wallner, Johanna Dolensky, Werner Seebacher, Eva-Maria Pferschy-Wenzig, Marcel Kaiser, Pascal Mäser, Robert Weis

**Affiliations:** 1 Pharmaceutical Chemistry, Institute of Pharmaceutical Sciences, University of Graz, Schubertstraße 1, 8010 Graz, Austria; 2 Pharmacognosy, Institute of Pharmaceutical Sciences, University of Graz, Beethovenstraße 8, 8010 Graz, Austria; 3 Swiss Tropical and Public Health Institute, Kreuzstraße 2, Allschwil, CH-4123 Basel, Switzerland; 4 Faculty of Philosophy and Natural Sciences, Swiss Tropical and Public Health Institute Petersplatz 1, University of Basel, CH-4003 Basel, Switzerland

**Keywords:** malaria, MMV, *Plasmodium falciparum*, cytotoxicity, PAMPA

## Abstract

MMV’s Malaria Box compound MMV030666 shows multi-stage activity against various strains of *Plasmodium falciparum* and lacks resistance development. To evaluate the importance of its diarylether partial structure, diarylthioethers and diphenylamines with varying substitution patterns were prepared. A number of evident structure-activity relationships were revealed. Physicochemical and pharmacokinetic parameters were determined experimentally (passive permeability) or calculated. Compared to the lead compound a diarylthioether was more active and less cytotoxic resulting in an excellent selectivity index of 850. In addition, pharmacokinetic and physicochemical parameters were improved.

## 1. Introduction

With an estimated 241 million cases and 627,000 deaths in 2020 the hard-earned reduction in malaria casualties fell victim to disruptions in prevention and care due to the COVID-19 pandemic. Furthermore, the number of malaria endemic countries rose from 26 in 2000 to 47 in 2020 [1]. Malaria belongs to the infectious diseases and is caused by eukaryotic, single-celled protozoans of the species *Plasmodium*. Various strains are human pathogens with *Plasmodium falciparum* being the most dangerous and deadly [2]. Emerging resistance to the gold standard in malaria therapy, the artemisinins and their partner drugs in the WHO African region are a serious cause for concern, as these countries are among those most affected [3,4,5]. To this day, double or triple Artemisinin-based combination therapies show acceptable efficacy, nonetheless alternative treatments and orally applicable drugs with new modes of action are urgently needed to successfully fight the malaria parasite [6,7,8]. Vaccine development is a difficult task to undertake due to the parasite’s complex life cycle and multiple possible targets. RTS,S/AS01, the first vaccine against any parasitic diseases, shows promising features and activities, however only partial efficacy [9,10,11].

The Medicines for Malaria Venture (MMV) is one of many foundations that made it their business to find new strategies to combat the increasing risk of untreatable malaria. Therefore, they published results of a huge screening project, the so-called Malaria Box, a collection of 400 drug- and probe-like compounds with activities against various strains of *Plasmodia* [12,13,14]. One of these compounds, the 2-phenoxybenzamide 1, exhibits multi-stage activity against sexual, asexual and liver stages of *P. falciparum* and lacks resistance development in sub-lethal doses. It shows a metabolomic profile resembling atovaquone. On the one hand, it disrupts the mitochondrial electron transport-chain by inhibiting the dihydroorotate-dehydrogenase and the cytochrome *bc1* complex, on the other hand, an interaction with the digestive vacuole was detected [15,16].

**Figure 1:**
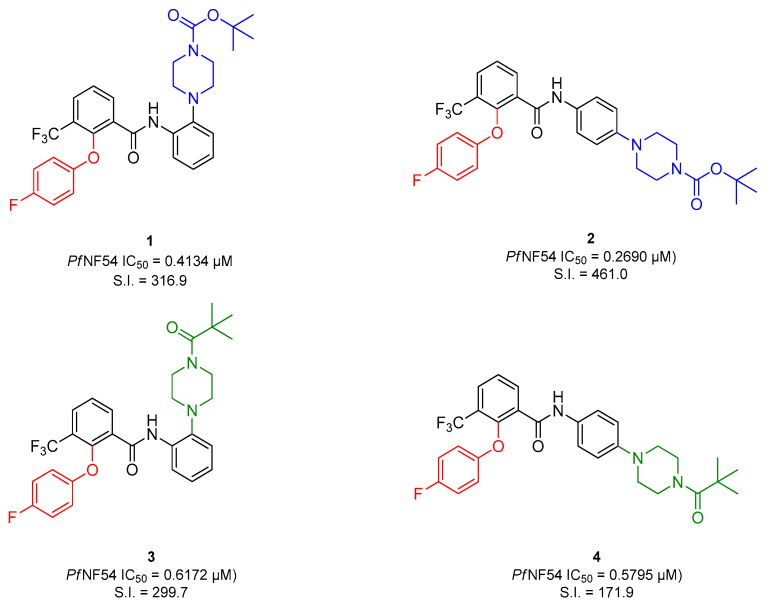
Structure-activity relationships of the lead compound **1** and its first series of derivatives [17].

Within our first study, we reported the retrosynthetic preparation of the lead compound **1** via a multi-step synthesis. Furthermore, a series of derivatives was prepared to gain first insights into structure-activity relationships. Thereby, the importance of the 2-phenoxy-group, as well as the beneficial effects of its 4-fluoro substituent on the antiplasmodial activity were determined. A shift of the *N*-*Boc*-piperazinyl group from 2′ to 4′ position of the benzanilide of the benzamide further increased the activity of compounds. Replacement of the *N*-*Boc*-group with a *N*-pivaloyl-group enhanced the acid-stability of compounds, whereby only a slight loss in activity was detected (Figure 1) [17]. This paper deals with the preparation of diversely substituted diarylethers, diarylthioethers and diphenylamines to gain further insights into structure-activity relationships.

## 2. Results and Discussion

### 2.1. Chemistry

**Figure 2:**
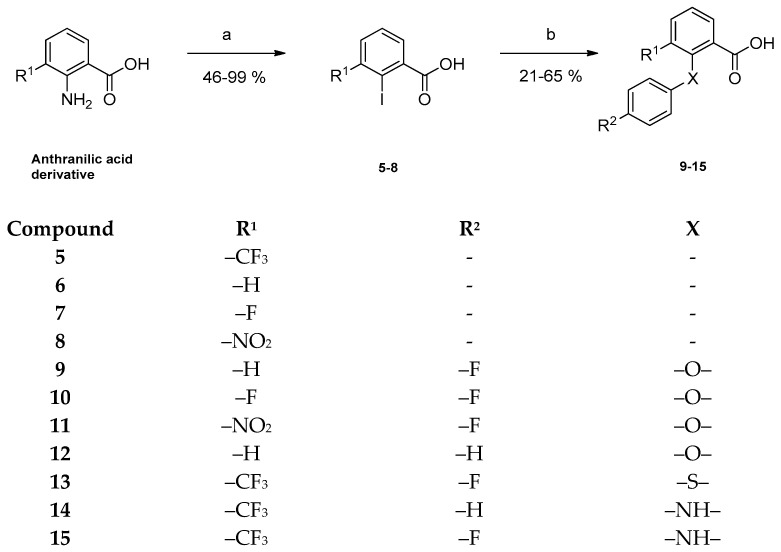
Preparation of the 2- and 3-substituted benzoic acid derivatives **9**–**15**. Reagents and conditions: (a) (1) H_2_SO_4_ 30%, dimethylsulfoxide (DMSO), 0 °C, 5 min; (2) NaNO_2_, rt, 2 h; (3) KI, H_2_O, rt, 1 h; (4) KI, H_2_O, rt, 1 h; (b) corresponding phenol or aniline, Cu, CuI, 1,8-diazabicyclo[5.4.0]undec-7-ene (DBU), dry pyridine, dry DMF, 160 °C, 2 h or 24 h.

New derivatives of the lead structure **1** were obtained by firstly synthesizing the corresponding carboxylic acid as well as anilino derivatives and subsequently coupling these partial structures to the desired benzamides. Preparation of the substituted benzoic acids started by treating the respective anthranilic acid with sodium nitrite under acidic conditions yielding the diazonium salts. Subsequent Sandmeyer-like reaction with an aqueous potassium iodide solution gave the desired 2-iodo-benzoic acid derivatives **5**, **6**, **7** and **8** as light brown solids in mostly high yields [18]. In our last study, only 2-phenoxybenzamides were prepared. In order to evaluate the importance of the diarylether partial structure, corresponding diarylthioethers and diphenylamines were prepared. In the course of a copper-catalyzed Ullmann-like ether synthesis, the obtained iodo-benzoic acids **5**, **6**, **7** and **8** were coupled with phenols or anilines, respectively, giving diarylethers (**9**–**12**), a diarylthioether (**13**) as well as diphenylamines (**14**, **15**) (Figure 2) [19]. Coupling was confirmed by the appearance of additional proton signals in the ^1^H NMR spectrum.

**Figure 3:**
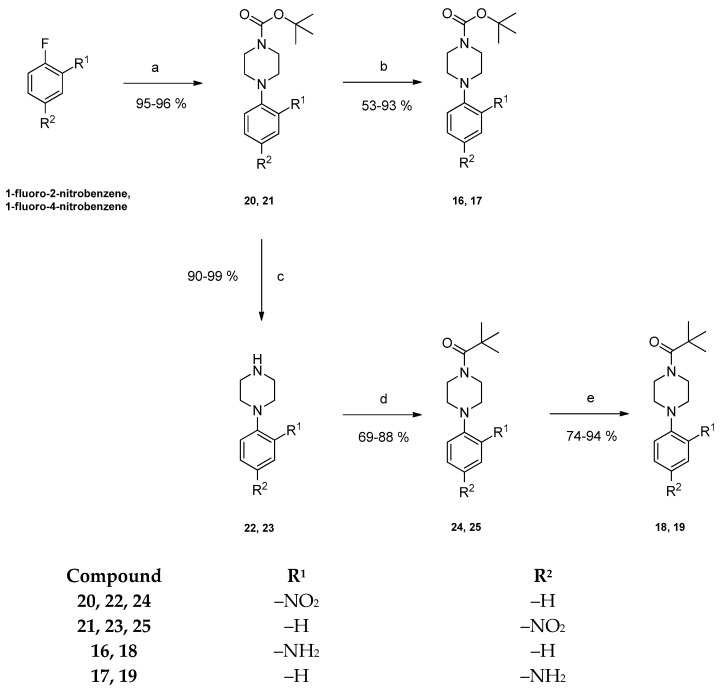
Preparation of anilino derivatives **16**–**19**. Reagents and conditions: (a) K_2_CO_3_, *N*-*Boc*-piperazine, dry DMSO, 80 °C, 72 h; (b) 15% (m/m) palladium on activated carbon, H_2_, methanol, rt, 24 h; (c) (1) dry CH_2_Cl_2_, 0 °C, 5 min; (2) trifluoroacetic acid, dry CH_2_Cl_2_, rt, 24 h; (d) pivaloyl chloride, triethylamine, dry CH_2_Cl_2_, rt, 24 h; (e) 15% (m/m) palladium on activated carbon, H_2_, methanol rt, 24 h.

In order to obtain the 2- and 4-substituted derivatives of aniline **16**, **17**, **18** and **19**, 1-fluoro-2-nitrobenzene and 1-fluoro-4-nitrobenzene were firstly treated with *N*-*Boc*-piperazine and potassium carbonate in dry DMSO in the course of a nucleophilic aromatic substitution yielding compounds **20** and **21** in high yields [20]. Their *N*-*Boc*-group was cleaved using trifluoroacetic acid in dry dichloromethane giving 1-(2-nitrophenyl)piperazine **22** and 1-(4-nitrophenyl)piperazine **23** [21]. Treatment of these piperazine derivatives with triethylamine and pivaloyl chloride in dry dichloromethane yielded the *N*-pivaloylpiperazinyl analogs **24** and **25** [22]. Subsequent reduction of the nitro group of compounds **20**, **21**, **24** and **25** with palladium in an atmosphere of hydrogen at the parr-apparatus gave the desired aromatic amines **16**, **17**, **18** and **19** (Figure 3) [23]. Successful reduction of the nitro group was detected in the ^1^H NMR spectrum by a shift of the aromatic protons to lower frequencies as well as by the appearance of a NH_2_-signal.

**Figure 4:**
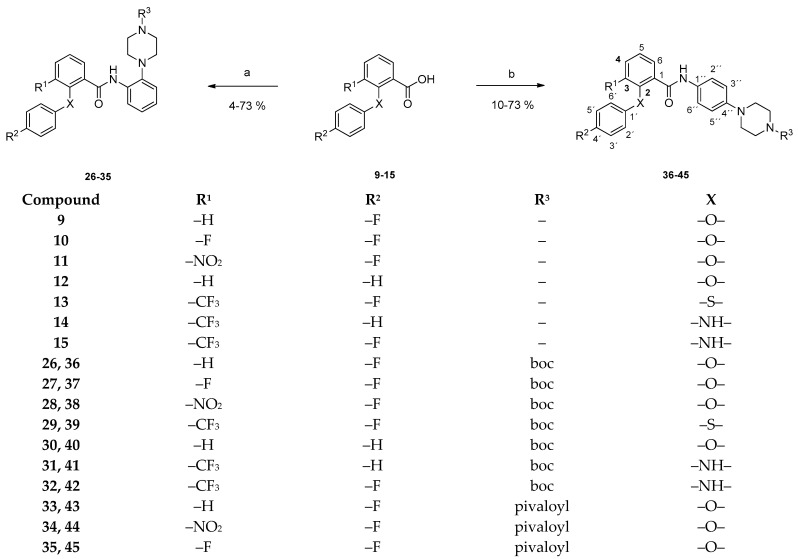
Preparation of compounds **26**–**45**. Reagents and conditions: (a) (1) amine **16**, dry CH_2_Cl_2_, 0 °C, 5 min; (2) 2-chloro-*N*-methylpyridinium iodide, DIPEA, rt, 24 h (compounds **26**–**28**, **30**–**32**) or (1) amine **16**, dry CH_2_Cl_2_, 0 °C, 5 min; (2) 2-chloro-*N*-methylpyridinium iodide, DIPEA, rt, 48 h (compound **29**) or (1) amine **18**, dry CH_2_Cl_2_, 0 °C, 5 min; (2) 2-chloro-*N*-methylpyridinium iodide, DIPEA, rt, 24 h (compounds **33**–**35**); (b) (1) amine **17**, dry CH_2_Cl_2_, 0 °C, 5 min; (2) 2-chloro-*N*-methylpyridinium iodide, DIPEA, rt, 24 h (compounds **36**–**42**) or (1) amine **19**, dry CH_2_Cl_2_, 0 °C, 5 min; (2) 2-chloro-*N*-methyl-pyridinium iodide, diisopropylethylamine, rt, 24 h (compounds **43**–**45**).

Amide synthesis of carboxylic acids and anilino derivatives was achieved using a combination of 2-chloro-*N*-methylpyridinium iodide (Mukaiyama reagent) and diisopropylethylamine (DIPEA) in dry dichloromethane (Figure 4) [24]. Efficient amide bond formation was detected in the ^1^H NMR spectrum. The NH resonance was shifted 6 ppm downfield.

Coupling of the benzoic acid derivatives **9**–**15** with the 2-substituted anilino derivatives **16** and **18** gave the *N*-[2-(4-*N*-*Boc*-piperazinyl)phenyl]benzamides and the *N*-[2-(4-*N*-pivaloyl-piperazinyl)phenyl]benzamides **26**–**35**. Reaction of the carboxylic acids with the 4-substituted aniline analogs **17** and **19** on the other hand, yielded the *N*-[4-(4-*N*-*Boc*-piperazinyl)phenyl]benzamides **36**–**42**, as well as the *N*-[4-(4-*N*-pivaloylpiperazinyl)phenyl]benzamides **43**–**45**.

**Figure 5:**
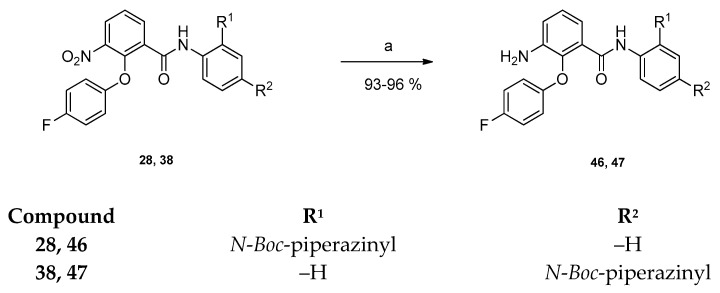
Preparation of compounds **46** and **47**. Reagents and conditions: (a) 15% (m/m) palladium on activated carbon, H_2_, methanol, rt, 24 h.

In order to evaluate the influence of an amino-group compared to electron withdrawing trifluoromethyl or nitro groups in ring position 3 of the benzamide, compounds **46** and **47** were prepared. The nitro group of **28** and **38** was reduced with palladium in an atmosphere of hydrogen at the parr-apparatus yielding compounds **46** and **47** (Figure 5) [23]. The reduction was detected by the appearance of a singlet for two amino protons in the ^1^H NMR spectrum.

### 2.2. Antiplasmodial Activity and Cytotoxicity

All newly prepared compounds were tested for their in vitro activity against the chloroquine sensitive strain NF54 of *P. falciparum*. Cytotoxicity was determined using L-6 cells (rat skeletal myofibroblasts). Chloroquine and podophyllotoxin were used as standards. Results obtained are summarized in Table 1.

MMVs Malaria Box compound **1** exhibits sub micromolar antiplasmodial activity (*Pf*NF54 IC_50_ = 0.4134 µM) and promising selectivity (S.I. = 316.9). Apart from the lead compound, its *para*-substituted analog **2** as well as their *N*-pivaloyl analogs **3** and **4** served as comparisons for the newly synthesized derivatives.

Replacing the 3-trifluoromethyl group of **1** with other electron-withdrawing groups or hydrogen yielded compounds **26**–**28** with slightly decreased antiplasmodial activities (*Pf*NF54 IC_50_ = 0.5908–0.6496 µM) and selectivity indices (S.I. = 153.9–245.5). However, their amino analog **46** showed reduced activity (*Pf*NF54 IC_50_ = 1.010 µM) and selectivity (S.I. = 126.0). A stark loss of activities was observed in the groups of *para*-substituted analogs as well as in both *N*-pivaloyl groups. Substitution of the 3-trifluoromethyl group of **2** (*Pf*NF54 IC_50_ = 0.2690 µM; S.I. = 461.0) by above-mentioned groups gave compounds **36**–**38** and **47** with distinctly reduced activity (*Pf*NF54 IC_50_ = 1.432–4.072 µM) and selectivity (S.I. = 8.676–59.88). The substitution of the 3-trifluoromethyl group of the *N*-pivaloyl analogs **3** (*Pf*NF54 IC_50_ = 0.6172 µM; S.I. = 299.7) and **4** (*Pf*NF54 IC_50_ = 0.5790 µM; S.I. = 171.8) by electron-withdrawing groups or hydrogen gave compounds **33**–**35** (*Pf*NF54 IC_50_ = 2.138–2.613 µM; S.I. = 7.275–43.75) and **43**–**45** (*Pf*NF54 IC_50_ = 1.498–5.615 µM; S.I. = 32.84–96.41), respectively, with markedly decreased antiplasmodial activities and selectivities.

Replacing the 2-(4-fluorophenoxy) group of **1** with a phenoxy group yielded compound **30** with moderate activity (*Pf*NF54 IC_50_ = 1.131 µM) and selectivity (S.I. = 19.87). In comparison the anilino analogs **31** and **32** showed only slightly decreased activity (*Pf*NF54 IC_50_ = 0.6142–0.6364 µM) and selectivity (S.I. = 193.8–209.6). However, the most promising variation in the pattern was the substitution with a 4-(fluorophenyl)sulfanyl group. Compound **29** exhibited distinctly improved selectivity (*Pf*NF54 IC_50_ = 0.1946 µM) and activity (S.I. = 850.5). The same changes were observed for the *para*-substituted analogs. The 2-phenoxy derivate **40** (*Pf*NF54 IC_50_ = 3.094 µM; S.I. = 31.78) was moderately active, but the anilino derivates **41** and **42** (*Pf*NF54 IC_50_ = 0.5389–0.6336 µM; S.I. = 53.29–174.8) were only slightly less active than **2**. Again, the 4-(fluorophenyl)sulfanyl derivate **39** (*Pf*NF54 IC_50_ = 0.1494 µM; S.I. = 212.8) was the most active.

In summary, highest antiplasmodial activity and selectivity was observed for compounds with a trifluoromethyl group in ring position 3 and a 4-(fluorophenyl)sulfanyl or a 4-fluorophenoxy substituent in ring position 2 of the benzamide. The anilino moiety should be substituted by a 4-bocpiperazinyl group in ring positions 2 or 4.

### 2.3. Physicochemical and Pharmacokinetic Properties

In addition to in vitro activity and cytotoxicity tests, some key pharmacokinetic parameters were calculated (log P, log D, LE) or determined experimentally (*Pe*). Results obtained are summarized in Table 2. The log P and log D_7.4_ values of compounds range from 4.73 up to 7.68. Ligand efficiency (LE) is an important parameter in early drug development. It is defined as the maximum in vitro binding affinity achievable by ligands, that is 1.5 kcal per mole per heavy atom (HA, non-hydrogen atom). The higher the LE value, the higher the binding affinity [25,26]. The calculated values range from 0.200 up to 0.236 kcal/mol/HA. Compounds **29** and **39** with the most promising antiplasmodial activities also exhibit highest ligand efficiencies of 0.230 and 0.234 kcal/mol/HA, respectively.

The parallel artificial membrane permeability assay (PAMPA) is a fast and easy high-throughput assay to determine passive permeability of compounds through semi-permeable membranes (for example the blood-brain-barrier) without the influence of efflux pumps or transporter molecules. Permeability is defined using hydrochlorothiazide (*Pe* = 0.09 × 10^−6^ cm/s) and caffeine (*Pe* = 8.00 × 10^−6^ cm/s) as standards. Compounds with a permeability higher than 1.5 × 10^−6^ cm/s are considered to be highly permeable. Permeability through a semi-permeable membrane could be detected for all compounds except **26**, **27**, **33** and **44** due to insufficient solubility in DMSO and methanol. Compounds **29** and **39** with the highest antiplasmodial activity also exhibit very promising permeability of 4.31 and 3.77 × 10^−6^ cm/s, respectively. The unsubstituted 2-phenoxybenzamides **30** (*Pe* = 8.52 × 10^−6^ cm/s) and **40** (*Pe* = 5.57 × 10^−6^ cm/s) as well as the *N*-pivaloylpiperazinyl derivatives **34** (*Pe* = 6.92 × 10^−6^ cm/s) and **45** (*Pe* = 5.13 × 10^−6^ cm/s) exhibit the by far highest permeabilities.

## 3. Materials and Methods

### 3.1. Instrumentation and Chemicals

IR spectra were acquired using a Bruker Alpha Platinum ATR FTIR spectrometer (Bruker, Ettlingen, Germany) (preparation of KBr discs). HRMS: Q Exactive Hybrid Quadrupole-Orbitrap mass spectrometer (Thermo Fisher Scientific, Waltham, MA, USA) run by Thermo Q Exactive 2.9 (Thermo Fisher Scientific, Waltham, MA, USA) and Thermo Xcalibur^TM^ Software Version 4.4 (Thermo Fisher Scientific, Waltham, MA, USA). The structures of all new compounds were determined by one- and two-dimensional NMR spectroscopy using a Bruker Avance Neo 400 MHz spectrometer, 5 mm tubes and TMS as internal standard. Shifts in ^1^H NMR (400 MHz) and ^13^C NMR (100 MHz) are reported in ppm; ^1^H- and ^13^C-resonances were assigned using ^1^H,^1^H- and ^1^H,^13^C-correlation spectra and are numbered as given in Figure 4. Signal multiplicities are abbreviated as follows: br, broad; d, doublet; dd, doublet of doublets; ddd, doublet of doublet of doublets; dt, doublet of triplets; m, multiplet; q, quartet; t, triplet; td, triplet of doublet; s, singlet. Melting points were determined using an Electrothermal IA 9200 melting point apparatus (Fisher Scientific, Birmingham, UK).

Materials: thin layer chromatography (TLC): TLC plates silica gel 60 (F254 (Merck); column chromatography (CC): silica gel 60 (Merck 70–230 mesh, pore diameter 60 Å), flash silica gel (VWR 230–400 mesh, pore diameter 60 Å or Merck 230–400 mesh, pore diameter 60 Å); PAMPA: 96-well pre-coated Corning Gentest PAMPA plate system (Corning, Glendale, AZ, USA), 96-well UV-star Microplates (Greiner Bio-One, Kremsmünster, Austria), SpectraMax M3 UV plate-reader (Molecular Devices, San Jose, CA, USA), ^1^H NMR and ^13^C NMR spectra of new compounds are available in the Supplementary Materials Section (Figures S1–S27).

### 3.2. Syntheses

#### 3.2.1. General Procedure for the Synthesis of Compounds **5**–**8**

The corresponding anthranilic acid (6.00 mmol) was dissolved in (DMSO) (11 mL) and the solution was ice-cooled. Upon adding 11 mL of 30% aq sulfuric acid (H_2_SO_4_), the reaction mixture was stirred at 0 °C for 5 min. After that, the ice bath was removed and sodium nitrate (13.28 mmol) was added. The reaction mixture was stirred at room temperature for 2 h. Subsequently, a solution of KI (10.92 mmol) in 5 mL of demineralized water was added dropwise with a syringe via a septum. The mixture was stirred for another hour before adding a second portion of KI (6.00 mmol) dissolved in 3 mL of aqua dest. After stirring for 1 h at ambient temperature, 50 mL of ethyl acetate was added. The aqueous and organic phases were separated. The organic phase was washed with water and brine, dried over anhydrous sodium sulfate, filtered and the solvent was removed in vacuo. The respective residues were purified by recrystallization from water.

2-Iodo-3-(trifluoromethyl)benzoic acid (**5**): The reaction of 3-(trifluoromethyl)anthranilic acid (2.11 g (10.33 mmol)) dissolved in DMSO (17 mL) and 30% aq H_2_SO_4_ (17 mL) with NaNO_2_ (1.54 g (22.35 mmol)) and KI (4.74 g (28.54 mmol)) in water (17 mL) gave the raw benzoic acid. It was purified by recrystallization from water (10 mL) giving compound **5** as brownish solid (2.97 g (91%)). m.P. 134 °C. NMR data were in accordance with literature data [27].

2-Iodobenzoic acid (**6**): The reaction of anthranilic acid (831 mg (6.06 mmol)) dissolved in DMSO (11 mL) and 30% aq H_2_SO_4_ (11 mL) with NaNO_2_ (921 mg (13.35 mmol)) and KI (2.83 g (17.02 mmol)) in water (8 mL) gave the raw iodobenzoic acid. It was purified by recrystallization from water (7 mL) yielding compound **6** as brown solid (1.48 g (99%)). m.P. 160 °C. NMR data were in accordance with literature data [28].

3-Fluoro-2-iodobenzoic acid (**7**): The reaction of 2-amino-3-fluorobenzoic acid (313 mg (2.02 mmol)) dissolved in DMSO (4 mL) and 30% aq H_2_SO_4_ (4 mL) with NaNO_2_ (309 mg (4.48 mmol)) and KI (939 mg (5.66 mmol)) in water (3 mL) yielded the raw product. It was purified by recrystallization from water (4 mL) giving compound **7** as brown solid (245 mg (46%)). m.P. 153 °C. NMR data were in accordance with literature data [29].

2-Iodo-3-nitrobenzoic acid (**8**): The reaction of 2-amino-3-nitrobenzoic acid (558 mg (3.06 mmol)) dissolved in DMSO (6 mL) and 30% aq H_2_SO_4_ (6 mL) with NaNO_2_ (460 mg (6.67 mmol)) and KI (1.42 g (8.55 mmol)) in water (4 mL) gave the raw product. It was purified by recrystallization from water (5 mL) yielding compound **8** as brown solid 799 mg (89%)). m.P. 207 °C. NMR data were in accordance with literature data [30].

#### 3.2.2. General Procedure for the Synthesis of Compounds **9**–**15**

The corresponding 2-iodobenzoic acid derivative (4.00 mmol) was dissolved in dry DMF (32 mL). The respective phenol, thiophenol or aniline (4.20 mmol), catalytic amounts of copper (0.53 mmol) and copper (I) iodide (0.18 mmol), 1,8-diazabicyclo[5.4.0]undec-7-ene (12.00 mmol) and dry pyridine (0.80 mmol) were added. The reaction mixture was refluxed in an oil bath at 160 °C for 2–24 h. Then, it was cooled to ambient temperature and acidified with 2 N HCl to a pH of 1. Ice and dichloromethane were added. Phases were separated. The aqueous phase was extracted twice with dichloromethane. The organic phases were combined, washed with water and brine, dried over anhydrous sodium sulfate and filtered. The solvent was evaporated in vacuo. The crude products were subsequently purified by column chromatography.

2-(4-Fluorophenoxy)benzoic acid (**9**): The reaction of compound **6** (688 mg (2.77 mmol)) with 4-fluorophenol (334 mg (2.98 mmol)), copper (31 mg (0.49 mmol)), copper (I) iodide (37 mg (0.19 mmol)), DBU (1.27 g (8.32 mmol)) and dry pyridine (44 mg (0.56 mmol)) in dry DMF (23 mL) for 2 h gave the crude product. It was purified by column chromatography (flash silica gel, cyclohexane (CH)/ethyl acetate (EtAc)/*Et*OH/AcOH) 9:1 (3:1:0.08)) yielding compound **9** as white amorphous solid (386 mg (60%)). NMR data were in accordance with literature data [30].

3-Fluoro-2-(4-fluorophenoxy)benzoic acid (**10**): To a solution of compound **7** (595 mg (2.24 mmol)) in dry DMF (18 mL), 4-fluorophenol ((263 mg (2.35 mmol)), copper (19 mg (0.30 mmol)), copper (I) iodide (19 mg (0.10 mmol)), DBU (1.02 g (6.72 mmol)) and dry pyridine (36 mg (0.45 mmol)) were added. The reaction mixture was refluxed for 2 h giving the crude product. It was purified by column chromatography (silica gel, CH_2_Cl_2_/*Me*OH/AcOH 75:1:1) yielding compound **10** as yellow amorphous solid (240 mg (43%)). m.P. 134 °C. IR = 3427, 1703, 1504, 1468, 1268, 1222, 1190, 770; ^1^H NMR (CDCl_3_) δ = 6.84–6.88 (m, 2H, 2′-H, 6′-H), 6.95–7.01 (m, 2H, 3′-H, 5′-H), 7.29 (td, *J* = 8.1, 4.8 Hz, 1H, 5-H), 7.40 (ddd, *J* = 10.0, 8.3, 1.7 Hz, 1H, 4-H), 7.86 (dt, *J* = 7.9, 1.5 Hz, 1H, 6-H); ^13^C NMR (CDCl_3_) δ = 116.09 (d, *J* = 23.6 Hz, C-3′, C-5′), 116.72 (d, *J* = 8.2 Hz, C-2′, C-6′), 122.02 (d, *J* = 18.7 Hz, C-4), 125.44 (C-1), 125.69 (d, *J* = 7.5 Hz, C-5), 127.75 (d, *J* = 3.6 Hz, C-6), 143.08 (q, *J* = 13.1 Hz, C-2), 153.92 (t, *J* = 2.3 Hz, C-1′), 155.63 (d, *J* = 252 Hz, C-3), 158.44 (d, *J* = 241 Hz, C-4′), 167.73 (C=O); HRMS (ESI−) calcd for C_13_H_7_F_2_O_3_^−^ [M − H]^−^: 249.0363; found: 249.0364.

2-(4-Fluorophenoxy)-3-nitrobenzoic acid (**11**): The reaction of compound **8** (2.46 g (8.41 mmol)) with 4-fluorophenol (1.00 g (8.92 mmol)), copper (83 mg (1.31 mmol)), copper (I) iodide (76 mg (0.40 mmol)), DBU (3.84 g (25.23 mmol) and dry pyridine (132 mg (1.67 mmol)) in dry DMF (68 mL) for 2 h gave the crude product. It was purified by column chromatography (silica gel, CH_2_Cl_2_/*Me*OH/AcOH 149:1:1) yielding compound 11 as pale orange amorphous solid (1.52 g (65%)). IR = 3077, 1707, 1539, 1504, 1447, 1359, 1309, 1249, 1221, 1180, 1096, 829, 782, 695; ^1^H NMR (CDCl_3_) δ = 6.74–6.78 (m, 2H, 2′-H, 6′-H), 6.93–7.00 (m, 2H, 3′-H, 5′-H), 7.48 (t, *J* = 8.0 Hz, 1H, 5-H), 8.12 (dd, *J* = 8.1, 1.8 Hz, 1H, 4-H), 8.25 (dd, *J* = 7.9, 1.8 Hz, 1H, 6-H); ^13^C NMR (CDCl_3_) δ = 116.15 (d, *J* = 23.6 Hz, C-3′, C-5′), 116.65 (d, *J* = 8.4 Hz, C-2′, C-6′), 125.45 (C-5), 126.23 (C-1), 130.06 (C-4), 136.67 (C-6), 144.92 (C-3), 148.06 (C-2), 154.04 (d, *J* = 2.4 Hz, C-1′), 158.41 (d, *J* = 241 Hz, C-4′), 167.94 (C=O); HRMS (ESI−): calcd for C_13_H_7_FNO_5_^−^ [M − H]^−^: 276.0308; found: 276.0308.

2-Phenoxybenzoic acid (**12**): The reaction of compound **6** (992 mg (4.00 mmol)) with phenol (395 mg (4.2 mmol)), copper (34 mg (0.53 mmol)), copper (I) iodide (34 mg (0.18 mmol)), DBU (1.83 g (12.00 mmol)) and dry pyridine (63 mg (0.80 mmol)) in dry DMF (32 mL) gave the crude product. It was purified by column chromatography (silica gel, CH/EtAc/EtOH/AcOH 160:30:9.2:0.8) yielding compound **12** as white amorphous solid (554 mg (65%)). NMR data were in accordance with literature data [30].

2-[(4-Fluorophenyl)sulfanyl]-3-(trifluoromethyl)benzoic acid (**13**): To a solution of compound **5** (1.90 g (6.02 mmol)) in dry DMF (48 mL), 4-fluorothiophenol (806 mg (6.29 mmol)), copper (76 mg (1.20 mmol)), copper (I) iodide (60 mg (0.32 mmol)), DBU (2.74 g (17.99 mmol)) and dry pyridine (94 mg (1.19 mmol)) were added. The reaction mixture was refluxed for 24 h giving the crude product. It was purified by column chromatography (flash silica gel, CH_2_Cl_2_/*Me*OH/AcOH 149:1:1) yielding compound **13** as light brown amorphous solid (199 mg (21%)). IR = 3066, 1714, 1582, 1492, 1315, 1274, 1231, 1209, 1135, 827, 676; ^1^H NMR (DMSO-d_6_) δ = 7.04–7.14 (m, 4H, 2′-H, 3′-H, 5′-H, 6′-H), 7.75 (t, *J* = 7.8 Hz, 1H, 5-H), 7.88 (d, *J* = 7.8 Hz, 1H, 6-H), 7.99 (d, *J* = 7.8 Hz, 1H, 4-H), 13.74 (br, 1H, OH); ^13^C NMR (DMSO-d_6_) δ = 116.11 (d, *J* = 22.3 Hz, C-3′, C-5′), 123.28 (q, *J* = 274 Hz, CF3), 127.83 (C-2), 128.72 (q, *J* = 5.7 Hz, C-4), 130.00 (d, *J* = 8.2 Hz, C-2′, C-6′), 130.67 (C-5), 132.12 (d, *J* = 3.1 Hz, C-1′), 132.52 (C-6), 133.04 (q, *J* = 29.1 Hz, C-3), 143.31 (C-1), 160.78 (d, *J* = 244 Hz, C-4′), 167.86 (C=O); HRMS (ESI−): calcd for C_14_H_7_F_4_O_2_S^−^ [M − H]^−^: 315.0109; found: 315.0114.

2-Anilino-3-(trifluoromethyl)benzoic acid (**14**): Reaction of compound **5** (1.26 g (4.00 mmol)) with aniline (391 mg (4.20 mmol)), copper (34 mg (0.53 mmol)), copper (I) iodide (34 mg (0.18 mmol)), DBU (1.83 mmol (12.00 mmol)) and dry pyridine (63 mg (0.80 mmol)) in dry DMF (32 mL) gave the crude product. It was purified by column chromatography (silica gel, CH_2_Cl_2_/*Me*OH/AcOH 29:1:0.3) yielding compound **14** as pale brown amorphous solid (231 mg (21%)). IR = 3344, 1674, 1594, 1497, 1453, 1317, 1253, 1136, 1090, 757, 665; ^1^H NMR (CDCl_3_) δ = 6.75–6.77 (m, 2H, 2′-H, 6′-H), 6.94–6.98 (m, 1H, 4′-H), 7.18–7.24 (m, 2H, 3′-H, 5′-H), 7.36 (t, *J* = 7.9 Hz, 1H, 5-H), 7.89 (dd, *J* = 7.9, 1.6 Hz, 1H, 4-H), 8.30 (dd, *J* = 7.9, 1.6 Hz, 1H, 6-H); ^13^C NMR (CDCl_3_) δ = 117.77 (C-2′, C-6′), 122.69 (C-4′), 123.49 (q, *J* = 274 Hz, CF_3_), 124.13 (C-5), 125.63 (C-1), 125.84 (q, *J* = 30.2 Hz, C-3), 129.32 (C-3′, C-5′), 132.55 (q, *J* = 5.2 Hz, C-4), 136.07 (C-6), 141.70 (br, C-2), 144.78 (C-1′), 168.62 (C=O); HRMS (ESI+): calcd for C_14_H_11_F_3_NO_2_^+^ [M + H]^+^: 282.0736; found: 282.0729.

2-(4-Fluoroanilino)-3-(trifluoromethyl)benzoic acid (**15**): Reaction of compound **5** (2.28 g (7.23 mmol)) with 4-fluoroaniline (707 mg (7.60 mmol)), copper (60 mg (0.96 mmol)), copper (I) iodide (62 mg (0.33 mmol)), DBU (3.31 g (21.00 mmol)) and dry pyridine (115 mg (1.45 mmol)) in dry DMF (58 mL) for 24 h gave the crude product. It was purified by column chromatography (flash silica gel, CH_2_Cl_2_/*Me*OH/AcOH 29:1:0.2) yielding compound 15 as yellow amorphous solid (564 mg (26%)). IR = 3330, 1671, 1594, 1508, 1445, 1308, 1264, 1219, 1171, 1128, 1090, 763, 685; ^1^H NMR (CDCl_3_) δ = 6.75–6.79 (m, 2H, 2′-H, 6′-H), 6.89–6.95 (m, 2H, 3′-H, 5′-H), 7.28 (t, *J* = 7.9 Hz, 1H, 5-H), 7.87 (dd, *J* = 7.9, 1.7 Hz, 1H, 4-H), 8.25 (dd, *J* = 7.9, 1.7 Hz, 1H, 6-H); ^13^C NMR (CDCl_3_) δ = 115.88 (d, *J* = 22.8 Hz, C-3′, C-5′), 120.36 (d, *J* = 8.0 Hz, C-2′, C-6′), 123.06 (C-5), 123.48 (q, *J* = 274 Hz, CF_3_), 123.79 (C-1), 124.78 (q, *J* = 30.3 Hz, C-3), 133.03 (q, *J* = 5.3 Hz, C-4), 136.12 (C-6), 140.93 (br, C-1′), 143.14 (C-2), 158.83 (d, *J* = 242 Hz, C-4′), 169.57 (C=O); HRMS (ESI+): calcd for C_14_H_10_F_4_NO_2_^+^ [M + H]^+^: 300.0642; found: 300.0635.

#### 3.2.3. General Procedure for the Synthesis of Compounds **20** and **21**

*N*-*Boc*-piperazine (14.00 mmol) and potassium carbonate (14 mmol) were suspended in dry DMSO (40 mL). The corresponding fluoronitrobenzene (7.00 mmol) was added and the suspension was refluxed at 80 °C for 72 h. The reaction mixture was cooled to ambient temperature and acidified with 2 N HCl to a pH of 1. Phases were separated. The aqueous phase was extracted with diethyl ether. The organic phases were combined, washed with ice water and brine, dried over anhydrous sodium sulfate and filtered. The solvent was evaporated in vacuo yielding the pure products.

*tert*-Butyl 4-(2-nitrophenyl)piperazine-1-carboxylate (**20**): The 1-fluoro-2-nitrobenzene (1.00 g (7.10 mmol)) was refluxed with *N*-*Boc*-piperazine (2.64 g (14.20 mmol)) and potassium carbonate (1.96 g (14.20 mmol)) in dry DMSO giving compound **20** as orange oil (2.07 g (95%)). It was used without further purification. NMR data were in accordance with literature data [23].

*tert-*Butyl 4-(4-nitrophenyl)piperazine-1-carboxylate (21): The reaction of 1-fluoro-4-nitrobenzene (988 mg (7.00 mmol)) with *N*-*Boc*-piperazine (2.69 g (14.44 mmol)) and potassium carbonate (1.94 g (14.02 mmol)) in dry DMSO yielded compound **21** as orange amorphous solid (2.07 g (96%)) which was used without further purification. NMR data were in accordance with literature data [31].

#### 3.2.4. General Procedure for the Synthesis of Compounds **22** and **23**

The corresponding *N*-*Boc*-piperazinyl derivative (1.00 mmol) was dissolved in dry dichloromethane (10 mL) and cooled to 0 °C in an ice bath. A solution of trifluoroacetic acid (30 mmol) in dry dichloromethane (3 mL) was added via a dropping funnel. After that, the ice bath was removed and the reaction mixture was stirred at room temperature for 24 h. Subsequently, the solvent and access trifluoroacetic acid were evaporated in vacuo. The residue was suspended in a solution of potassium carbonate (20 mmol) in water (6 mL). The aqueous suspension was extracted five times with dichloromethane/propan-2-ol 3:1. The combined organic phases were dried over anhydrous sodium sulfate and filtered. The solvent was evaporated in vacuo yielding the desired compounds as pure products.

1-(2-Nitrophenyl)piperazine (**22**): Compound **20** (2.24 g (7.30 mmol)) reacted with trifluoroacetic acid (5.00 g (43.80mmol)) in dry dichloromethane (95 mL) giving the protonated form of compound **22**. A solution of potassium carbonate (6.06 g (43.80 mmol)) in water (88 mL) was added to obtain compound **22** as orange oil (1.36 g (90%)) which was used without further purification. NMR data were in accordance with literature data [32].

1-(4-Nitrophenyl)piperazine (**23**): The reaction of compound **21** (1.02 g (3.33 mmol)) with trifluoroacetic acid (4.45 g (39.00 mmol)) in dry dichloromethane (42 mL) gave the protonated product **23**. Work-up with an aqueous solution of potassium carbonate (2.70 g (19.56 mmol)) in 40 mL of water yielded compound **23** as brownish amorphous solid (683 mg (99%)) which was used without further purification. NMR data were in accordance with literature data [33].

#### 3.2.5. General Procedure for the Synthesis of Compounds **24** and **25**

The corresponding piperazinyl derivative (2.00 mmol) was dissolved in dry dichloromethane (8 mL) and cooled to 0 °C in an ice bath. Dry triethylamine (3.00 mmol) was added dropwise. Pivaloyl chloride (2.10 mmol) was added with a syringe via a septum. The ice bath was removed and the reaction mixture was stirred at ambient temperature for 24 h. After that, water (30 mL) was added. The aqueous and organic phases were separated. The organic phase was washed with 2 N HCl, 8% aq NaHCO_3_ and brine, dried over anhydrous sodium sulfate and filtered. The solvent was evaporated in vacuo yielding the pivaloylpiperazine derivatives which were used without further purification.

2,2-Dimethyl-1-[4-(2-nitrophenyl)piperazin-1-yl]propan-1-one (**24**): The reaction of the piperazinyl derivative **22** (555 mg (2.68 mmol)) with dry triethylamine (815 mg (8.05 mmol)) and pivaloyl chloride (340 mg (2.82 mmol)) in dry dichloromethane (11 mL) yielded compound **24** as yellow amorphous solid (322 mg (69%)) which was used without further purification. NMR data were in accordance with literature data [17].

2,2-Dimethyl-1-[4-(4-nitrophenyl)piperazin-1-yl]propan-1-one (**25**): The reaction of compound **23** (414 mg (2.00 mmol)) with dry triethylamine (607 mg (6.00 mmol)) and pivaloyl chloride (253 mg (2.10 mmol)) in dry dichloromethane (8 mL) gave the pivaloylpiperazinyl derivative **25** as orange amorphous solid (513 mg (88%)) which was used without further purification. NMR data were in accordance with literature data [17].

#### 3.2.6. General Procedure for the Synthesis of Compounds **16**–**19**, **46** and **47**

To a solution of 15% (m/m) palladium on activated carbon in dry methanol (100 mL) the corresponding nitro compound (2.00 mmol) was added. Reduction of the nitro group was performed in an atmosphere of hydrogen (50 psi) at the parr apparatus at ambient temperature for 24 h. The reaction mixture was filtered and the solvent was removed in vacuo yielding the desired anilino-derivatives that were either purified by column chromatography or used without further purification.

*tert*-Butyl 4-(2-aminophenyl)piperazine-1-carboxylate (**16**): The nitro group of compound **20** (3.67 g (11.93 mmol)) was reduced with PdC (560 mg) in dry methanol (100 mL) to obtain the raw anilino derivative. It was purified by column chromatography (silica gel, CH_2_Cl_2_/*Me*OH 79:1) yielding compound **16** as pale brown amorphous solid (1.75 g (53%)). NMR data were in accordance with literature data [23].

*tert*-Butyl 4-(4-aminophenyl)piperazine-1-carboxylate (**17**): The reaction of compound **21** (1.98 g (6.45 mmol)) with PdC (299 mg) in dry methanol (100 mL) yielded the anilino derivative **17** as dark red oil (1.66 g (93%)) which was used without further purification. NMR data were in accordance with literature data [34].

1-[4-(2-Aminophenyl)piperazin-1-yl]-2,2-dimethylpropan-1-one (**18**): The nitro group of compound **24** (555 mg (1.90 mmol)) was reduced with PdC (111 mg) in dry methanol (90 mL) to obtain compound **18** as grey amorphous solid (367 mg (74%)) which was used without further purification. NMR data were in accordance with literature data [17].

1-[4-(4-Aminophenyl)piperazin-1-yl]-2,2-dimethylpropan-1-one (**19**): The nitro group of compound **25** (410 mg (1.41 mmol)) was reduced with PdC (69 mg) in dry methanol (100 mL) yielding the anilino derivative **19** as dark red oil (346 mg (94%)) which was used without further purification. NMR data were in accordance with literature data [17].

*tert*-Butyl 4-{2-[3-amino-2-(4-fluorophenoxy)benzamido]phenyl}piperazine-1-carboxylate (**46**): The reaction of compound **28** (294 mg (0.55 mmol)) with PdC (42 mg) in dry methanol (100 mL) gave the pure product **46** as white amorphous solid (258 mg (93%)). IR = 3354, 1692, 1619, 1498, 1452, 1365, 1249, 1191, 765; ^1^H NMR (CDCl_3_) δ = 1.50 (s, 9H, (CH_3_)_3_), 2.81–2.84 (m, 4H, N(CH_2_)_2_), 3.63 (br, 4H, N(CH_2_)_2_), 3.85 (s, 2H, NH_2_), 6.81–6.84 (m, 2H, 2′-H, 6′-H), 6.88–6.92 (m, 2H, 3′-H, 5′-H), 6.97 (dd, *J* = 8.0, 1.5 Hz, 1H, 4-H), 7.02–7.07 (m, 1H, 4″-H), 7.09–7.15 (m, 2H, 3″-H, 5″-H), 7.20 (t, *J* = 7.9 Hz, 1H, 5-H), 7.42 (dd, *J* = 7.8, 1.5 Hz, 1H, 6-H), 8.42 (d, *J* = 8.1 Hz, 1H, 6″-H), 9.70 (s, 1H, NH); ^13^C NMR (CDCl_3_) δ = 28.42 ((CH_3_)_3_), 44.05 (N(CH_2_)_2_), 52.12 (N(CH_2_)_2_), 80.07 (C*Me*_3_), 116.26 (d, *J* = 8.1 Hz, C-2′, C-6′), 116.46 (d, *J* = 23.6 Hz, C-3′, C-5′), 119.58 (C-4), 120.14 (C-3″), 120.26 (C-6″), 120.32 (C-6), 124.00 (C-4″), 125.54 (C-5″), 126.66 (C-5), 133.49 (C-1″), 138.49 (C-2), 140.26 (C-3), 141.23 (C-2″), 152.77 (d, *J* = 2.4 Hz, C-1′), 154.72 (N(C=O)O), 158.33 (d, *J* = 243 Hz, C-4′), 163.28 ((C=O)NH); HRMS (ESI+): calcd for C_28_H_32_FN_4_O_4_^+^ [M + H]^+^: 507.2408; found: 507.2416.

*tert*-Butyl 4-{4-[3-amino-2-(4-fluorophenoxy)benzamido]phenyl}piperazine-1-carboxylate (**47**): The nitro group of compound **38** (157mg (0.29 mmol)) was reduced with PdC (22 mg) in dry methanol (80 mL) yielding the pure compound **47** as pale yellow amorphous solid (142 mg (96%)). IR = 3396, 1687, 1516, 1499, 1474, 1417, 1366, 1323, 1231, 1196, 830, 767; ^1^H NMR (CDCl_3_) δ = 1.48 (s, 9H, (CH_3_)_3_), 3.04–3.08 (m, 4H, N(CH_2_)_2_), 3.54–3.57 (m, 4H, N(CH_2_)_2_), 3.80 (s, 2H, NH_2_), 6.84–6.91 (m, 4H, 2′-H, 3″-H, 5″-H, 6′-H), 6.94–7.00 (m, 3H, 3′-H, 4-H, 5′-H), 7.20 (t, *J* = 7.8 Hz, 1H, 5-H), 7.35 (d, *J* = 8.6 Hz, 2H, 2″-H, 6″-H), 7.53 (d, *J* = 7.7 Hz, 1H, 6-H), 8.71 (s, 1H, NH); ^13^C NMR (CDCl_3_) δ = 28.40 ((CH_3_)_3_), 43.57 (N(CH_2_)_2_), 49.75 (N(CH_2_)_2_), 79.89 (C*Me*_3_), 115.90 (d, *J* = 8.2 Hz, C-2′, C-6′), 116.70 (d, *J* = 23.6 Hz, C-3′, C-5′), 117.16 (C-3″, C-5″), 119.72 (C-4), 121.02 (C-6), 121.43 (C-2″, C-6″), 126.73 (C-5), 128.79 (C-1), 130.83 (C-1″), 138.01 (C-2), 148.30 (C-4″), 152.26 (d, *J* = 2.4 Hz, C-1′), 154.68 (N(C=O)O), 158.51 (d, *J* = 242 Hz, C-4′), 162.72 ((C=O)NH); HRMS (ESI−): calcd for C_28_H_30_FN_4_O_4_^−^ [M − H]^−^: 505.2251; found: 505.2251.

#### 3.2.7. General Procedure for the Synthesis of Compounds **26**–**45**

The corresponding benzoic acid derivative (1.00 mmol) and the anilino derivative (1.00 mmol) were dissolved in dry dichloromethane (30 mL) and cooled in an ice bath to 0 °C. The Mukaiyama reagent (1.75 mmol) and DIPEA (5.00 mmol) were added. The reaction mixture was stirred at room temperature for 24–48 h. After that, 20% aq ammonium chloride (50 mL) was added. Phases were separated. The aqueous phase was extracted twice with EtAc. The organic phases were combined, washed with 8% aq NaHCO_3_ and brine, dried over anhydrous sodium sulfate and filtered. The solvent was evaporated in vacuo yielding the raw products that were subsequently purified.

*tert*-Butyl 4-{2-[2-(4-fluorophenoxy)benzamido]phenyl}piperazine-1-carboxylate (**26**): Reaction of the carboxylic acid **9** (238 mg (1.02 mmol) with the amine **16** (281 mg (1.01 mmol)), 2-chloro-*N*-methylpyridinium iodide (451 mg (1.77 mmol) and DIPEA (646 mg (5.00 mmol)) in dry dichloromethane (30 mL) gave the raw benzamide. It was purified by column chromatography (silica gel, CH/EtAc 6:1) yielding compound **26** as pale orange amorphous solid (363 mg (73%)). IR = 3318, 2971, 1694, 1662, 1589, 1516, 1501, 1453, 1395, 1364, 1309, 1277, 1203, 1114, 854, 766; ^1^H NMR (CDCl_3_) δ = 1.48 (s, 9H, (CH_3_)_3_), 2.75–2.79 (m, 4H, N(CH_2_)_2_), 3.32 (br, 4H, N(CH_2_)_2_), 6.79 (br d, *J* = 8.3 Hz, 1H, 3H), 7.06–7.13 (m, 6H, 2′-H, 3′-H, 3″-H, 4″-H, 5′-H, 6′-H), 7.17–7.25 (m, 2H, 5-H, 5″-H), 7.41 (td, *J* = 7.8, 1.7 Hz, 1H, 4-H), 8.34 (dd, *J* = 7.9, 1.7 Hz, 1H, 6-H), 8.60 (d, *J* = 8.1 Hz, 1H, 6″-H), 10.45 (s, 1H, NH); ^13^C NMR (CDCl_3_) δ = 28.40 ((CH_3_)_3_), 43.73 (N(CH_2_)_2_), 52.21 (N(CH_2_)_2_), 79.90 (C*Me*_3_), 117.00 (d, *J* = 23.5 Hz, C-3′, C-5′), 117.32 (C-3), 120.35 (C-3″), 121.04 (C-6″), 122.39 (d, *J* = 8.4 Hz, C-2′, C-6′), 123.57 (C-5), 124.08 (C-1), 124.13 (C-4″), 125.59 (C-5″), 132.69 (C-6), 133.04 (C-4), 134.01 (C-1″), 141.99 (C-2″), 151.10 (d, *J* = 2.8 Hz, C-1′), 154.80 (N(C=O)O), 156.17 (N), 159.83 (d, *J* = 245 Hz, C-4′), 162.77 ((C=O)NH); HRMS (ESI+) calcd for C_28_H_31_FN_3_O_4_^+^ [M + H]^+^: 492.2299; found: 492.2301.

*tert*-Butyl 4-{4-[3-fluoro-2-(4-fluorophenoxy)benzamido]phenyl}piperazin-1-carboxylate (**27**): Reaction of the carboxylic acid 10 (100 mg (0.40 mmol)) with the amine **16** (111 mg (0.40 mmol)), 2-chloro-*N*-methylpyridinium iodide (179 mg (0.70 mmol)) and DIPEA (259 mg (2.00 mmol)) in dry dichloromethane (12 mL) gave the crude product. It was purified by column chromatography (flash silica gel, petroleum ether/EtAc 5.5:3) yielding compound **27** as white amorphous solid (62 mg (30%)). IR = 3441, 1692, 1668, 1501, 1455, 1267, 1178, 765; ^1^H NMR (CDCl_3_) δ = 1.50 (s, 9H, (CH_3_)_3_), 2.77–2.81 (m, 4H, N(CH_2_)_2_), 3.54 (br, 4H, N(CH_2_)_2_), 6.95 (br s, 2H, 3′-H, 5′-H), 6.97 (br s, 2H, 2′-H, 6′-H), 7.06–7.14 (m, 2H, 3″-H, 4″-H), 7.18 (br td, *J* = 7.4, 2.0 Hz, 1H, 5″-H), 7.29–7.35 (m, 2H, 4-H, 5-H), 8.00–8.02 (m, 1H, 6-H), 8.50 (d, *J* = 8.1 Hz, 1H, 6″-H), 10.07 (s, 1H, NH); ^13^C NMR (CDCl_3_) δ = 28.43 ((CH_3_)_3_), 43.97 (N(CH_2_)_2_), 52.22 (N(CH_2_)_2_), 80.05 (C*Me*_3_), 116.42 (d, *J* = 23.7 Hz, C-3′, C-5′), 117.99 (d, *J* = 8.2 Hz, C-2′, C-6′), 120.36 (d, *J* = 18.6 Hz, C-4), 120.40 (C-3″), 120.75 (C-6″), 124.47 (C-4″), 125.58 (C-5″), 126.08 (d, *J* = 7.7 Hz, C-5), 126.87 (d, *J* = 3.5 Hz, C-6), 130.14 (C-1), 133.40 (C-1″), 141.44 (d, *J* = 12.6 Hz, C-2), 141.75 (C-2″), 153.52 (t, *J* = 2.1 Hz, C-1′), 154.72 (N(C=O)O), 155.11 (d, *J* = 252 Hz, C-3), 158.91 (d, *J* = 243 Hz, C-4′), 161.73 (d, *J* = 3.1 Hz, ((C=O)NH)); HRMS (ESI+): calcd for C_28_H_30_F_2_N_3_O_4_^+^ [M + H]^+^: 510.2199; found: 510.2191.

*tert*-Butyl 4-{2-[2-(4-fluorophenoxy)-3-nitrobenzamido]phenyl}piperazine-1-carboxylate (**28**): Reaction of the carboxylic acid **11** (567 mg (2.05 mmol)) with the aniline **16** (555 mg (2.00 mmol)), 2-chloro-*N*-methylpyridinium iodide (895 mg (3.50 mmol)) and DIPEA (1292 mg (10.00 mmol)) in dry dichloromethane (60 mL) gave the raw product. It was purified by column chromatography (silica gel, CH/EtAc 3:1) yielding compound **28** as pale green amorphous solid (634 mg (59%)). IR = 3321, 1688, 1594, 1523, 1498, 1452, 1422, 1364, 1249, 1177, 1130, 837, 775; ^1^H NMR (CDCl_3_) δ = 1.50 (s, 9H, (CH_3_)_3_), 2.78–2.81 (m, 4H, N(CH_2_)_2_), 3.58 (br, 4H, N(CH_2_)_2_), 6.79–6.83 (m, 2H, 2′-H, 6′-H), 6.90–6.95 (m, 2H, 3′-H, 5′-H), 7.08–7.18 (m, 3H, 3″-H, 4″-H, 5″-H), 7.54 (t, *J* = 8.0 Hz, 1H, 5-H), 8.07 (dd, *J* = 8.0, 1.4 Hz, 1H, 4-H), 8.35–8.41 (m, 2H, 6-H, 6″-H), 9.81 (s, 1H, NH); ^13^C NMR (CDCl_3_) δ = 28.41 ((CH_3_)_3_), 44.20 (N(CH_2_)_2_), 52.29 (N(CH_2_)_2_), 80.15 (C*Me*_3_), 116.57 (d, *J* = 23.8 Hz, C-3′, C-5′), 117.36 (d, *J* = 8.3 Hz, C-2′, C-6′), 120.24 (C-6″), 120.61 (C-3″), 124.76 (C-4″), 125.76 (C-5″), 126.17 (C-5), 128.63 (C-4), 132.12 (C-1), 133.00 (C-1″), 136.07 (C-6), 141.48 (C-2″), 143.87 (C-3), 145.56 (C-2), 153.09 (d, *J* = 2.6 Hz, C-1′), 154.60 (N(C=O)O), 158.83 (d, *J* = 243 Hz, C-4′), 160.95 ((C=O)NH); HRMS (ESI+) calcd for C_28_H_30_FN_4_O_6_^+^ [M + H]^+^: 537.2149; found: 537.2155.

*tert*-Butyl 4-(2-{2-[(4-fluorophenyl)sulfanyl]-3-(trifluoromethyl)benz-amido}phenyl) piperazine-1-carboxylate (**29**): Reaction of the carboxylic acid **13** (235 mg (0.74 mmol)) with the amine **16** (212 mg (0.76 mmol)), 2-chloro-*N*-methylpyridinium iodide (339 mg (1.33 mmol)) and DIPEA (479 mg (3.70 mmol)) in dry dichloromethane (22 mL) for 48 h gave the raw product. It was purified by column chromatography (silica gel, CH/EtAc 4:1) yielding compound **29** as white amorphous solid (189 mg (44%)). IR = 3331, 2976, 1693, 1590, 1516, 1490, 1418, 1366, 1313, 1231, 1171, 828, 761, 687; ^1^H NMR (CDCl_3_) δ = 1.48 (s, 9H, (CH_3_)_3_), 2.78–2.81 (m, 4H, N(CH_2_)_2_), 3.47 (br, 4H, N(CH_2_)_2_), 6.75–6.79 (m, 2H, 3′-H, 5′-H), 7.05–7.13 (m, 3H, 2′-H, 4″-H, 6′-H), 7.15–7.22 (m, 2H, 3″-H, 5″-H), 7.58 (t, *J* = 7.7 Hz, 1H, 5-H), 7.75 (dd, *J* = 7.7, 1.4 Hz, 1H, 6-H), 7.89 (dd, *J* = 7.7, 1.4 Hz, 1H, 4-H), 8.34 (dd, *J* = 8.1, 1.5 Hz, 1H, 6″-H), 9.01 (s, 1H, NH); ^13^C NMR (CDCl_3_) δ = 28.39 ((CH_3_)_3_), 44.14 (N(CH_2_)_2_), 52.37 (N(CH_2_)_2_), 80.11 (C*Me*_3_), 116.16 (d, *J* = 22.2 Hz, C-3′, C-5′), 119.24 (C-6″), 120.86 (C-3″), 123.26 (q, *J* = 274 Hz, CF_3_), 124.32 (C-4″), 126.04 (C-5″), 128.48 (q, *J* = 5.7 Hz, C-4), 129.40 (C-5), 130.77 (d, *J* = 3.3 Hz, C-1′), 131.21 (C-2), 132.50 (d, *J* = 8.3 Hz, C-2′, C-6′), 132.75 (C-6), 133.35 (C-1″), 134.27 (q, *J* = 29.7 Hz, C-3), 140.89 (C-2″), 144.33 (C-1), 154.62 (N(C=O)O), 161.98 (d, *J* = 248 Hz, C-4′), 164.81 ((C=O)-NH); HRMS (ESI+): calcd for C_29_H_30_F_4_N_3_O_3_S^+^ [M + H]^+^: 576.1938; found: 576.1925.

*tert*-Butyl 4-[2-(2-phenoxybenzamido)phenyl]piperazin-1-carboxylate (**30**): Reaction of the carboxylic acid **12** (263 mg (1.23 mmol)) with the amine **16** (341 mg (1.23 mmol)), 2-chloro-*N*-methylpyridinium iodide (549 mg (2.15 mmol)) and DIPEA (795 mg (6.15 mmol)) in dry dichloromethane (37 mL) gave the crude product. It was purified by column chromatography (silica gel, CH/EtAc 3:1) yielding compound **30** as white amorphous solid (192 mg (33%)). IR = 3313, 2970, 1695, 1656, 1591, 1513, 1452, 1393, 1363, 1308, 1276, 1215, 1161, 1114, 762; ^1^H NMR (CDCl_3_) δ = 1.48 (s, 9H, ((CH_3_)_3_)), 2.74–2.78 (m, 4H, N(CH_2_)_2_), 3.30 (br, 4H, N(CH_2_)_2_), 6.83 (d, *J* = 8.2 Hz, 1H, 3-H), 7.05–7.15 (m, 4H, 2′-H, 3″-H, 4″-H, 6′-H), 7.17–7.26 (m, 3H, 4′-H, 5-H, 5″-H), 7.36–7.43 (m, 3H, 3′-H, 4-H, 5′-H), 8.36 (dd, *J* = 7.9, 1.9 Hz, 1H, 6-H), 8.60 (d, *J* = 8.0 Hz, 1H, 6″-H), 10.51 (br s, 1H, NH); ^13^C NMR (CDCl_3_) δ = 28.42 ((CH_3_)_3_), 43.56 (N(CH_2_)_2_), 52.18 (N(CH_2_)_2_), 79.73 (C*Me*_3_), 117.74 (C-3), 120.30 (C-3″), 120.86 (C-2′, C-6′), 121.10 (C-6″), 123.42 (C-5), 124.07 (C-4″), 124.08 (C-1), 125.31 (C-4′), 125.48 (C-5″), 130.27 (C-3′, C-5′), 132.58 (C-6), 132.98 (C-4), 134.03 (C-1″), 142.09 (C-2″), 154.79 (N(C=O)O), 155.29 (C-1′), 156.08 (C-2), 162.88 ((C=O)NH); HRMS (ESI+): calcd for C_28_H_32_N_3_O_4_^+^ [M + H]^+^: 474.2387; found: 474.2376.

*tert*-Butyl 4-{2-[2-anilino-3-(trifluoromethyl)benzamido]phenyl}piperazin-1-carboxylate (**31**): Reaction of the carboxylic acid **14** (376 mg (1.34 mmol)) with the amine **16** (370 mg (1.34 mmol)), 2-chloro-*N*-methylpyridinium iodide (598 mg (2.34 mmol)) and DIPEA (862 mg (6.67 mmol)) in dry dichloromethane (40 mL) gave the crude product. It was purified by column chromatography (flash silica gel, CH_2_Cl_2_/*Me*OH 149:1) obtaining compound **31** as pale yellow oil (50 mg (7%)). IR = 3302, 1693, 1649, 1594, 1518, 1453, 1366, 1323, 1251, 1171, 1132, 748, 691; ^1^H NMR (CDCl_3_) δ = 1.50 (s, 9H, (CH_3_)_3_), 2.73–2.76 (m, 4H, N(CH_2_)_2_), 3.55 (br, 4H, N(CH_2_)_2_), 6.23 (br s, 1H, NH), 6.70–6.74 (m, 2H, 2′-H, 6′-H), 6.78–6.83 (m, 1H, 4′-H), 6.99–7.03 (m, 1H, 4″-H), 7.05–7.11 (m, 4H, 3′-H, 3″-H, 5′-H, 5″-H), 7.39 (br t, *J* = 7.9 Hz, 1H, 5-H), 7.83 (dd, *J* = 7.9, 1.7 Hz, 1H, 4-H), 8.08 (dd, *J* = 7.9, 1.7 Hz, 1H, 6-H), 8.21 (br d, *J* = 7.6 Hz, 1H, 6″-H), 9.79 (s, 1H, NH); ^13^C NMR (CDCl_3_) δ = 28.41 ((CH_3_)_3_), 44.34 (N(CH_2_)_2_), 52.16 (N(CH_2_)_2_), 80.18 (C*Me*_3_), 116.39 (C-2′, C-6′), 119.76 (C-6″), 120.34 (C-3″), 121.93 (C-4′), 123.95 (q, *J* = 274 Hz, CF_3_), 124.05 (C-4″), 124.46 (C-5), 125.09 (q, *J* = 29.2 Hz, C-3), 125.66 (C-5″), 129.28 (C-3′, C-5′), 129.73 (q, *J* = 5.3 Hz, C-4), 132.87 (C-1), 133.20 (C-1″), 134.27 (C-6), 138.44 (q, *J* = 1.4 Hz, C-2), 141.13 (C-2″), 144.57 (C-1′), 154.64 (N(C=O)O), 164.07 ((C=O)NH); HRMS (ESI+): calcd for C_29_H_32_F_3_N_4_O_3_^+^ [M + H]^+^: 541.2421; found: 541.2409.

*tert*-Butyl 4-{2-[2-(4-fluoroanilino)-3-(trifluoromethyl)benzamido]phenyl}piperazin-1-carboxylate (**32**): Reaction of the carboxylic acid **15** (444 mg (1.48 mmol)) with the amine **16** (418 mg (1.50 mmol)), 2-chloro-*N*-methylpyridinium iodide (662 mg (2.58 mmol)) and DIPEA (957 mg (7.40 mmol)) in dry dichloromethane (45 mL) gave the crude product. It was purified by column chromatography (flash silica gel, CH_2_Cl_2_/*Me*OH 79+1) yielding compound **32** as pale yellow amorphous solid (22 mg (4%)). IR = 3328, 2977, 1693, 1591, 1508, 1453, 1366, 1319, 1169, 1034, 1001, 912, 824, 760, 690; ^1^H NMR (CDCl_3_) δ = 1.49 (s, 9H, (CH_3_)_3_), 2.75–2.78 (m, 4H, N(CH_2_)_2_), 3.54 (br, 4H, N(CH_2_)_2_), 6.26 (br s, 1H, NH), 6.67–6.71 (m, 2H, 2′-H, 6′-H), 6.76–6.81 (m, 2H, 3′-H, 5′-H), 7.01–7.12 (m, 3H, 3″-H, 4″-H, 5″-H), 7.37 (br t, *J* = 7.8 Hz, 1H, 5-H), 7.82 (dd, *J* = 7.8 Hz, 1H, 4-H), 8.03 (dd, *J* = 7.8, Hz, 1H, 6-H), 8.20 (d, *J* = 7.9 Hz, 1H, 6″-H), 9.68 (br s, 1H, NH); ^13^C NMR (CDCl_3_) δ = 28.40 ((CH_3_)_3_), 44.24 (N(CH_2_)_2_), 52.17 (N(CH_2_)_2_), 80.23 (C*Me*_3_), 115.90 (d, *J* = 22.9 Hz, C-3′, C-5′), 118.54 (d, *J* = 7.9 Hz, C-2′, C-6′), 119.64 (C-6″), 120.34 (C-3″), 123.93 (q, *J* = 273 Hz, CF_3_), 124.18 (C-5), 124.22 (C-4″), 124.69 (q, *J* = 29.4 Hz, C-3), 125.75 (C-5″), 129.80 (q, *J* = 5.2 Hz, C-4), 132.24 (C-1), 133.04 (C-1″), 134.13 (C-6), 139.10 (C-2), 140.78 (d, *J* = 2.6 Hz, C-1′), 141.05 (C-2″), 154.63 (N(C=O)O), 158.25 (d, *J* = 241 Hz, C-4′), 164.12 ((C=O)NH); HRMS (ESI+): calcd for C_29_H_31_F_4_N_4_O_3_^+^ [M + H]^+^: 559.2327; found: 559.2313.

*N*-{2-[4-(2,2-Dimethylpropanoyl)piperazin-1-yl]phenyl}-2-(4-fluorophenoxy)benzamid (**33**): Reaction of the carboxylic acid **9** (232 mg (1.00 mmol)) with the amine **18** (261 mg (1.00 mmol)), 2-chloro-*N*-methylpyridinium iodide (447 mg (1.75 mmol)) and DIPEA (646 mg (5.00 mmol)) in dry dichloromethane (30 mL) gave the crude product. It was purified by column chromatography (silica gel, CH/EtAc 3:1) yielding compound **33** as white amorphous solid (340 mg (72%)). IR = 3307, 1622, 1589, 1500, 1451, 1308, 1211, 1016, 750; ^1^H NMR (CDCl_3_) δ = 1.25 (s, 9H, (CH_3_)_3_), 2.78–2.82 (m, 4H, N(CH_2_)_2_), 3.51 (br, 4H, N(CH_2_)_2_), 6.79 (dd, *J* = 8.3, 1.1 Hz, 1H, 3-H), 7.07–7.14 (m, 6H, 2′-H, 3′-H, 3″-H, 4″-H, 5′-H, 6′-H), 7.19–7.27 (m, 2H, 5-H, 5″-H), 7.41 (ddd, *J* = 8.3, 7.3, 1.9 Hz, 1H, 4-H), 8.36 (dd, *J* = 7.9, 1.8 Hz, 1H, 6-H), 8.63 (dd, *J* = 8.3, 1.4 Hz, 1H, 6″-H), 10.50 (s, 1H, NH); ^13^C NMR (CDCl_3_) δ = 28.31 ((CH_3_)_3_), 38.59 (C*Me*_3_), 45.05 (N(CH_2_)_2_), 52.50 (N(CH_2_)_2_), 117.08 (d, *J* = 24.0 Hz, C-3′, C-5′), 117.10 (C-3), 120.49 (C-3″), 121.07 (C-6″), 122.60 (d, *J* = 8.4 Hz, C-2′, C-6′), 123.57 (C-5), 123.86 (C-1), 124.14 (C-4″), 125.81 (C-5″), 132.78 (C-6), 133.10 (C-4), 134.13 (C-1″), 141.51 (C-2″), 151.00 (d, *J* = 2.9 Hz, C-1′), 156.23 (C-2), 159.89 (d, *J* = 246 Hz, C-4′), 162.75 ((C=O)NH), 176.42 (C=O); HRMS (ESI+): calcd for C_28_H_31_FN_3_O_3_^+^ [M + H]^+^: 476.2344; found: 476.2332.

*N*-{2-[4-(2,2-Dimethylpropanoyl)piperazin-1-yl]phenyl}-2-(4-fluorophenoxy)-3-nitrobenzamid (**34**): Reaction of the carboxylic acid **11** (150 mg (0.54 mmol)) with the amine **18** (141 mg (0.54 mmol)), 2-chloro-*N*-methylpyridinium iodide (243 mg (0.95 mmol)) and DIPEA (349 mg (2.70 mmol)) in dry dichloromethane (16 mL) gave the crude product. It was purified by column chromatography (flash silica gel, CH_2_Cl_2_/CH/EtAc 3:2:1) yielding compound **34** as pale green amorphous solid (172 mg (61%)). IR = 3441, 1630, 1519, 1499, 1449, 1361, 1184, 775; ^1^H NMR (CDCl_3_) δ = 1.33 (s, 9H, (CH_3_)_3_), 2.81–2.85 (m, 4H, N(CH_2_)_2_), 3.79 (br, 4H, N(CH_2_)_2_), 6.80–6.84 (m, 2H, 2′-H, 6′-H), 6.91–6.96 (m, 2H, 3′-H, 5′-H), 7.08–7.19 (m, 3H, 3″-H, 4″-H, 5″-H), 7.55 (t, *J* = 8.0 Hz, 1H, 5-H), 8.06 (dd, *J* = 8.0, 1.8 Hz, 1H, 4-H), 8.37–8.42 (m, 2H, 6-H, 6″-H), 9.84 (s, 1H, NH); ^13^C NMR (CDCl_3_) δ = 28.43 ((CH_3_)_3_), 38.71 (C*Me*_3_), 45.48 (N(CH_2_)_2_), 52.55 (N(CH_2_)_2_), 116.64 (d, *J* = 23.8 Hz, C-3′, C-5′), 117.49 (d, *J* = 8.2 Hz, C-2′, C-6′), 120.37 (C-6″), 120.64 (C-3″), 124.81 (C-4″), 125.93 (C-5″), 126.20 (C-5), 128.67 (C-4), 131.98 (C-1), 133.04 (C-1″), 136.13 (C-6), 141.08 (C-2″), 143.92 (C-3), 145.63 (C-2), 153.04 (d, *J* = 2.6 Hz, C-1′), 158.89 (d, *J* = 244 Hz, C-4′), 160.93 ((C=O)NH), 176.48 (C=O); HRMS (ESI+): calcd for C_28_H_30_FN_4_O_5_^+^ [M + H]^+^: 521.2195; found: 521.2183.

*N*-{2-[4-(2,2-Dimethylpropanoyl)piperazin-1-yl]phenyl}-3-fluoro-2-(4-fluorophenoxy)benzamid (**35**): Reaction of the carboxylic acid **10** (100 mg (0.40 mmol)) with the amine **18** (105 mg (0.40 mmol)), 2-chloro-*N*-methylpyridinium iodide (179 mg (0.70 mmol)) and DIPEA (259 mg (2.00 mmol)) in dry dichloromethane (12 mL) gave the raw product. It was purified by column chromatography (flash silica gel, CH/EtAc 2.75:1) yielding compound **35** as white amorphous solid (114 mg (58%)). IR = 3331, 2928, 1670, 1631, 1579, 1499, 1454, 1267, 1184, 1015, 765; ^1^H NMR (CDCl_3_) δ = 1.31 (s, 9H, (CH_3_)_3_), 2.80–2.83 (m, 4H, N(CH_2_)_2_), 3.73 (br, 4H, N(CH_2_)_2_), 6.96–6.99 (m, 4H, 2′-H, 3′-H, 5′-H, 6′-H), 7.07–7.14 (m, 2H, 3″-H, 4″-H), 7.17–7.21 (m, 1H, 5″-H), 7.31–7.37 (m, 2H, 4-H, 5-H), 8.02–8.05 (m, 1H, 6-H), 8.52 (br d, *J* = 8.0 Hz, 1H, 6″-H), 10.11 (s, 1H, NH); ^13^C NMR (CDCl_3_) δ = 28.42 ((CH_3_)_3_), 38.70 (C*Me*_3_), 45.32 (N(CH_2_)_2_), 52.49 (N(CH_2_)_2_), 116.46 (d, *J* = 23.7 Hz, C-3′, C-5′), 118.23 (d, *J* = 8.4 Hz, C-2′, C-6′), 120.42 (d, *J* = 18.8 Hz, C-4), 120.49 (C-3″), 120.83 (C-6″), 124.50 (C-4″), 125.78 (C-5″), 126.07 (d, *J* = 7.6 Hz, C-5), 126.96 (d, *J* = 3.3 Hz, C-6), 129.93 (C-1), 133.49 (C-1″), 141.32 (C-2″), 141.59 (d, *J* = 12.4 Hz, C-2), 153.50 (t, *J* = 2.2 Hz, C-1′), 155.08 (d, *J* = 252 Hz, C-3), 157.05 (d, *J* = 243 Hz, C-4′), 161.71 (d, *J* = 3.2 Hz, (C=O)NH), 176.50 (C=O); HRMS (ESI+): calcd for C_28_H_30_F_2_N_3_O_3_^+^ [M + H]^+^: 494.2250; found: 494.2236.

*tert*-Butyl 4-{4-[2-(4-fluorophenoxy)benzamido]phenyl}piperazine-1-carboxylate (**36**): Reaction of the carboxylic acid **9** (240 mg (1.03 mmol)) with the amine **17** (281 mg (1.01 mmol)), 2-chloro-*N*-methylpyridinium iodide (449 mg (1.76 mmol) and DIPEA (646 mg (5.00 mmol)) in dry dichloromethane (30 mL) gave the raw product. It was purified by column chromatography (silica gel, CH/EtAc 2:1) yielding compound **36** as pale brown amorphous solid (136 mg (27%)). IR = 3376, 1689, 1662, 1597, 1538, 1501, 1476, 1449, 1414, 1365, 1317, 1282, 1233, 1202, 1119, 924, 866, 763; ^1^H NMR (CDCl_3_) δ = 1.48 (s, 9H, (CH_3_)_3_), 3.06–3.09 (m, 4H, N(CH_2_)_2_), 3.56–3.59 (m, 4H, N(CH_2_)_2_), 6.83 (d, *J* = 8.1 Hz, 1H, 3-H), 6.89–6.92 (m, 2H, 3″-H, 5″-H), 7.06–7.13 (m, 4H, 2′-H, 3′-H, 4′-H, 5′-H), 7.22–7.26 (m, 1H, 5-H), 7.41 (td, *J* = 8.1, 1.7 Hz, 1H, 4-H), 7.50–7.53 (m, 2H, 2″-H, 6″-H), 8.32 (dd, *J* = 7.9, 1.7 Hz, 1H, 6-H), 9.42 (s, 1H, NH); ^13^C NMR (CDCl_3_) δ = 28.41 ((CH_3_)_3_), 43.77 (N(CH_2_)_2_), 49.84 (N(CH_2_)_2_), 79.86 (C*Me*_3_), 116.94 (d, *J* = 23.6 Hz, C-3′, C-5′), 117.23 (C-3″, C-5″), 117.80 (C-3), 121.14 (d, *J* = 8.4 Hz, C-2′, C-6′), 121.68 (C-2″, C-6″), 123.96 (C-5), 124.07 (C-1), 131.09 (C-1″), 132.49 (C-6), 132.95 (C-4), 148.31 (C-4″), 151.07 (d, *J* = 2.7 Hz, C-1′), 154.66 (N(C=O)O), 155.42 (C-2), 159.64 (d, *J* = 244 Hz, C-4′), 162.33 ((C=O)NH); HRMS (ESI+) calcd for C_28_H_31_FN_3_O_4_^+^ [M + H]^+^: 492.2299; found: 492.2302.

*tert*-Butyl 4-{4-[3-fluoro-2-(4-fluorophenoxy)benzamido]phenyl}piperazine-1-carb-oxylate (**37**): Reaction of the carboxylic acid **10** (106 mg (0.42 mmol)) with the amine **17** (119 mg (0.43 mmol)), 2-chloro-*N*-methylpyridinium iodide (191 mg (0.75 mmol)) and DIPEA (273 mg (2.12 mmol)) in dry dichloromethane (13 mL) gave the raw product. It was purified by column chromatography (silica gel, CH_2_Cl_2_/EtAc 9:1) yielding compound **37** as light brown amorphous solid (92 mg (43%)). IR = 3276, 1688, 1654, 1601, 1517, 1501, 1468, 1415, 1365, 1326, 1266, 1232, 1183, 1122, 1002, 927, 881, 834, 768; ^1^H NMR (CDCl_3_) δ = 1.48 (s, 9H, (CH_3_)_3_), 3.06–3.09 (m, 4H, N(CH_2_)_2_), 3.55–3.58 (m, 4H, N(CH_2_)_2_), 6.87 (d, *J* = 8.9 Hz, 2H, 3″-H, 5″-H), 6.93–6.97 (m, 2H, 2′-H, 6′-H), 6.99–7.04 (m, 2H, 3′-H, 5′-H), 7.31–7.37 (m, 2H, 4-H, 5-H), 7.42 (d, *J* = 8.9 Hz, 2H, 2″-H, 6″-H), 8.05 (dd, *J* = 7.9, 1.9 Hz, 1H, 6-H), 9.02 (s, 1H, NH); ^13^C NMR (CDCl_3_) δ = 28.41 ((CH_3_)_3_), 43.52 (N(CH_2_)_2_), 49.68 (N(CH_2_)_2_), 79.88 (C*Me*_3_), 116.59 (d, *J* = 23.7 Hz, C-3′, C-5′), 116.72 (d, *J* = 8.3 Hz, C-2′, C-6′), 117.12 (C-3″, C-5″), 120.24 (d, *J* = 18.4 Hz, C-4), 121.56 (C-2″, C-6″), 126.24 (d, *J* = 7.6 Hz, C-5), 127.08 (d, *J* = 3.3 Hz, C-6), 129.39 (C-1), 130.52 (C-1″), 140.40 (d, *J* = 12.8 Hz, C-2), 148.53 (C-4″), 153.00 (t, *J* = 2.2 Hz, C-1′), 154.66 (N(C=O)O), 154.98 (d, *J* = 252 Hz, C-3), 158.84 (d, *J* = 243 Hz, C-4′), 161.17 (d, *J* = 3.1 Hz, ((C=O)NH)); HRMS (ESI+): calcd for C_28_H_30_F_2_N_3_O_4_^+^ [M + H]^+^: 510.2199; found: 510.2190.

*tert*-Butyl 4-{4-[2-(4-fluorophenoxy)-3-nitrobenzamido]phenyl}piperazine-1-carb-oxylate (**38**): Reaction of the carboxylic acid **11** (562 mg (2.03 mmol)) with the amine **17** (557 mg (2.01 mmol)), 2-chloro-*N*-methylpyridinium iodide (898 mg (3.51 mmol)) and DIPEA (1292 mg (10.00 mmol)) in dry dichloromethane (60 mL) gave the raw product. It was purified by column chromatography (silica gel, CH_2_Cl_2_/acetonitrile 12:1) yielding compound **38** as yellow amorphous solid (415 mg (39%)). IR = 3422, 1672, 1534, 1500, 1418, 1365, 1229, 1174, 828, 776; ^1^H NMR (CDCl_3_) δ = 1.48 (s, 9H, (CH_3_)_3_), 3.06–3.10 (m, 4H, N(CH_2_)_2_), 3.54–3.58 (m, 4H, N(CH_2_)_2_), 6.83–6.88 (m, 4H, 2′-H, 3″-H, 5″-H, 6′-H), 6.96–7.01 (m, 2H, 3′-H, 5′-H), 7.32 (d, *J* = 8.8 Hz, 2H, 2″-H, 6″-H), 7.54 (t, *J* = 8.0 Hz, 1H, 5-H), 8.07 (dd, *J* = 8.1, 1.7 Hz, 1H, 4-H), 8.43 (dd, *J* = 7.9, 1.7 Hz, 1H, 6-H), 8.66 (s, 1H, NH); ^13^C NMR (CDCl_3_) δ = 28.40 ((CH_3_)_3_), 43.48 (N(CH_2_)_2_), 49.51 (N(CH_2_)_2_), 79.93 (C*Me*_3_), 116.59 (d, *J* = 8.3 Hz, C-2′, C-6′), 116.81 (d, *J* = 22.9 Hz, C-3′, C-5′), 117.00 (C-3″, C-5″), 121.70 (C-2″, C-6″), 126.30 (C-5), 128.60 (C-4), 129.80 (C-1″), 131.13 (C-1), 136.57 (C-6), 143.82 (C-3), 144.96 (C-2), 148.80 (C-4″), 152.85 (d, *J* = 2.5 Hz, C-1′), 154.65 (N(C=O)O), 158.89 (d, *J* = 243 Hz, C-4′), 160.47 ((C=O)NH); HRMS (ESI−) calcd for C_28_H_28_FN_4_O_6_^−^ [M − H]^−^: 535.1993; found: 535.1989.

*tert*-Butyl 4-(4-{2-[(4-fluorophenyl)sulfanyl]-3-(trifluoromethyl)benzamido}phenyl) piperazine-1-carboxylate (**39**): Reaction of the carboxylic acid **13** (212 mg (0.67 mmol)) with the amine **17** (190 mg (0.69 mmol)), 2-chloro-*N*-methylpyridinium iodide (300 mg (1.17 mmol)) and DIPEA (433 mg (3.35 mmol)) in dry dichloromethane (20 mL) gave the raw product. It was purified by column chromatography (silica gel, CH_2_Cl_2_/*Me*OH 79:1) yielding compound **39** as pale brown amorphous solid (66 mg (17%)). IR = 3424, 1656, 1518, 1423, 1313, 1231, 1128, 830; ^1^H NMR (CDCl_3_) δ = 1.49 (s, 9H, (CH_3_)_3_), 3.09–3.12 (m, 4H, N(CH_2_)_2_), 3.57–3.60 (m, 4H, N(CH_2_)_2_), 6.82–6.90 (m, 4H, 3′-H, 3″-H, 5′-H, 5″-H), 7.06–7.10 (m, 2H, 2′-H, 6′-H), 7.26–7.30 (m, 2H, 2″-H, 6″-H), 7.58 (t, *J* = 7.8 Hz, 1H, 5-H), 7.70 (s, 1H, NH), 7.84 (d, *J* = 7.8 Hz, 1H, 6-H), 7.89 (d, *J* = 7.8 Hz, 1H, 4-H); ^13^C NMR (CDCl_3_) δ = 28.42 ((CH_3_)_3_), 43.40 (N(CH_2_)_2_), 49.67 (N(CH_2_)_2_), 79.94 (C*Me*_3_), 116.38 (d, *J* = 22.2 Hz, C-3′, C-5′), 117.08 (C-3″, C-5″), 120.96 (C-2″, C-6″), 123.25 (q, *J* = 274 Hz, CF_3_), 128.55 (q, *J* = 5.7 Hz, C-4), 129.50 (C-5), 130.25 (C-1″), 130.36 (C-2), 130.92 (d, *J* = 3.2 Hz, C-1′), 131.66 (d, *J* = 8.1 Hz, C-2′, C-6′), 133.67 (C-6), 134.32 (q, *J* = 29.5 Hz, C-3), 143.80 (C-1), 148.53 (C-4″), 154.68 (N(C=O)O), 161.95 (d, *J* = 248 Hz, C-4′), 164.48 ((C=O)NH); HRMS (ESI+): calcd for C_29_H_30_F_4_N_3_O_3_S^+^ [M + H]^+^: 576.1938; found: 576.1927.

*tert*-Butyl 4-[4-(2-phenoxybenzamido)phenyl]piperazin-1-carboxylate (**40**): Reaction of the carboxylic acid **12** (291 mg (1.36 mmol)) with the amine **17** (378 mg (1.36 mmol)), 2-chloro-*N*-methylpyridinium iodide (608 mg (2.38 mmol)) and DIPEA (879 mg (6.80 mmol)) in dry dichloromethane (40 mL) gave the crude product. It was purified by column chromatography (silica gel, CH/EtAc 2.5:1) yielding compound **40** as white amorphous solid (262 mg (40%)). IR = 3372, 1691, 1662, 1598, 1537, 1514, 1475, 1449, 1415, 1364, 1317, 1282, 1217, 1163, 1119, 923, 829, 753; ^1^H NMR (CDCl_3_) δ = 1.48 (s, 9H, ((CH_3_)_3_)), 3.06–3.09 (m, 4H, N(CH_2_)_2_), 3.55–3.58 (m, 4H, N(CH_2_)_2_), 6.87–6.91 (m, 3H, 3-H, 3″-H, 5″-H), 7.11 (d, *J* = 7.9 Hz, 2H, 2′-H, 6′-H), 7.20–7.27 (m, 2H, 4′-H, 5-H), 7.40–7.44 (m, 3H, 3′-H, 4-H, 5′-H), 7.51 (d, *J* = 8.9 Hz, 2H, 2″-H, 6″-H), 8.33 (d, *J* = 7.9, 1.8 Hz, 1H, 6-H), 9.51 (s, 1H, NH); ^13^C NMR (CDCl_3_) δ = 28.41 ((CH_3_)_3_), 43.52 (N(CH_2_)_2_), 49.86 (N(CH_2_)_2_), 79.85 (C*Me*_3_), 117.23 (C-3″, C-5″), 118.44 (C-3), 119.46 (C-2′, C-6′), 121.66 (C-2″, C-6″), 123.95 (C-5), 124.24 (C-1), 124.85 (C-4′), 130.27 (C-3′, C-5′), 131.20 (C-1″), 132.41 (C-6), 132.90 (C-4), 148.24 (C-4″), 154.67 (N(C=O)O), 155.14 (C-2), 155.36 (C-1′), 162.40 ((C=O)NH); HRMS (ESI+): calcd for C_28_H_32_N_3_O_4_^+^ [M + H]^+^: 474.2387; found: 474.2376.

*tert*-Butyl 4-{4-[2-anilino-3-(trifluoromethyl)benzamido]phenyl}piperazin-1-carboxylate (**41**): Reaction of the carboxylic acid **14** (230 mg (0.82 mmol)) with the amine **17** (227 mg (0.82 mmol)), 2-chloro-*N*-methylpyridinium iodide (367 mg (1.44 mmol)) and DIPEA (530 mg (4.10 mmol)) in dry dichloromethane (25 mL) gave the crude product. It was purified by column chromatography (flash silica gel, CH/EtAc 3:1) yielding compound **41** as pale brown amorphous solid (44 mg (10%)). IR = 3320, 1705, 1640, 1597, 1528, 1453, 1420, 1316, 1232, 1167, 1131, 753; ^1^H NMR (CDCl_3_) δ = 1.47 (s, 9H, (CH_3_)_3_), 3.02–3.05 (m, 4H, N(CH_2_)_2_), 3.52–3.55 (m, 4H, N(CH_2_)_2_), 6.09 (br s, 1H, NH), 6.75–6.79 (m, 4H, 2′-H, 3″-H, 5″-H, 6′-H), 6.89–6.93 (m, 1H, 4′-H), 7.05–7.09 (m, 2H, 2″-H, 6″-H), 7.17–7.22 (m, 2H, 3′-H, 5′-H), 7.44 (td, *J* = 7.9 Hz, 1H, 5-H), 7.84 (dd, *J* = 7.9, 1.6 Hz, 1H, 4-H), 8.34 (dd, *J* = 7.9, 1.6 Hz, 1H, 6-H), 9.42 (br s, 1H, NH); ^13^C NMR (CDCl_3_) δ = 28.41 ((CH_3_)_3_), 43.53 (N(CH_2_)_2_), 49.65 (N(CH_2_)_2_), 79.88 (C*Me*_3_), 115.95 (C-2′, C-6′), 116.93 (C-3″, C-5″), 121.23 (q, *J* = 274 Hz, CF_3_), 122.18 (C-2″, C-4′, C-6″), 125.37 (C-5), 125.53 (q, *J* = 29.2 Hz, C-3), 129.71 (C-3′, C-5′), 129.80 (q, *J* = 5.5 Hz, C-4), 130.08 (C-1″), 132.57 (C-1), 135.49 (C-6), 137.41 (q, *J* = 1.3 Hz, C-2), 144.60 (C-1′), 148.48 (C-4″), 154.68 (N(C=O)O), 163.11 ((C=O)NH); HRMS (ESI+): calcd for C_29_H_32_F_3_N_4_O_3_^+^ [M + H]^+^: 541.2421; found: 541.2410.

*tert*-Butyl 4-{4-[2-(fluoroanilino)-3-(trifluoromethyl)benzamido]phenyl}piperazin-1-carboxylate (**42**): Reaction of the carboxylic acid **15** (299 mg (1.00 mmol)) with the carboxylic acid **17** (277 mg (1.00 mmol)), 2-chloro-*N*-methylpyridinium iodide (447 mg (1.75 mmol)) and DIPEA (646 mg (5.00 mmol)) in dry dichloromethane (30 mL) gave the crude product. It was purified by column chromatography (flash silica gel, CH_2_Cl_2_/*Me*OH 79:1) yielding compound **42** as pale brown amorphous solid (124 mg (22%)). IR = 3427, 1665, 1508, 1454, 1315, 1229, 1167, 824; ^1^H NMR (CDCl_3_) δ = 1.48 (s, 9H, (CH_3_)_3_), 3.04–3.07 (m, 4H, N(CH_2_)_2_), 3.53–3.56 (m, 4H, N(CH_2_)_2_), 6.05 (br s, 1H, NH), 6.72–6.76 (m, 2H, 2′-H, 6′-H), 6.79–6.81 (m, 2H, 3″-H, 5″-H), 6.85–6.90 (m, 2H, 3′-H, 5′-H), 7.13 (d, *J* = 8.9 Hz, 2H, 2″-H, 6″-H), 7.42 (t, *J* = 7.9 Hz, 1H, 5-H), 7.83 (dd, *J* = 7.9, 1.6 Hz, 1H, 4-H), 8.28 (dd, *J* = 7.9, 1.6 Hz, 1H, 6-H), 9.24 (s, 1H, NH); ^13^C NMR (CDCl_3_) δ = 28.41 ((CH_3_)_3_), 43.38 (N(CH_2_)_2_), 49.62 (N(CH_2_)_2_), 79.89 (C*Me*_3_), 116.22 (d, *J* = 22.9 Hz, C-3′, C-5′), 116.97 (C-3″, C-5″), 117.80 (d, *J* = 7.9 Hz, C-2′, C-6′), 121.89 (C-2″, C-6″), 123.94 (q, *J* = 273 Hz, CF_3_), 125.13 (q, *J* = 29.2 Hz, C-3), 125.16 (C-5), 129.79 (q, *J* = 5.2 Hz, C-4), 130.00 (C-1″), 132.16 (C-1), 135.45 (C-6), 137.86 (q, *J* = 1.8 Hz, C-2), 140.74 (d, *J* = 2.5 Hz, C-1′), 148.53 (C-4″), 154.68 (N(C=O)O), 158.34 (d, *J* = 241 Hz, C-4′), 163.13 ((C=O)NH); HRMS (ESI+): calcd for C_29_H_31_F_4_N_4_O_3_^+^ [M + H]^+^: 559.2327; found: 559.2311.

*N*-{4-[4-(2,2-Dimethylpropanoyl)piperazin-1-yl]phenyl}-2-(4-fluorophenoxy)benzamid (**43**): Reaction of the carboxylic acid **9** (232 mg (1.00 mmol)) with the amine **19** (261 mg (1.00 mmol)), 2-chloro-*N*-methylpyridinium iodide (447 mg (1.75 mmol)) and DIPEA (646 mg (5.00 mmol)) in dry dichloromethane (30 mL) gave the crude product. It was purified by column chromatography (silica gel, CH/EtAc 1:1) yielding compound **43** as pale yellow amorphous solid (158 mg (33%)). IR = 3385, 1654, 1614, 1518, 1504, 1477, 1419, 1321, 1205, 857; ^1^H NMR (CDCl_3_) δ = 1.31 (s, 9H, (CH_3_)_3_), 3.10–3.13 (m, 4H, N(CH_2_)_2_), 3.78–3.81 (m, 4H, N(CH_2_)_2_), 6.83 (d, *J* = 7.8 Hz, 1H, 3-H), 6.90 (d, *J* = 9.0 Hz, 2H, 3″-H, 5″-H), 7.06–7.14 (m, 4H, 2′-H, 3′-H, 5′-H, 6′-H), 7.23–7.28 (m, 1H, 5-H), 7.42 (ddd, *J* = 9.0, 7.9, 1.8 Hz, 1H, 4-H), 7.53 (d, *J* = 9.0 Hz, 2H, 2″-H, 6″-H), 8.32 (dd, *J* = 7.9, 1.9 Hz, 1H, 6-H), 9.42 (s, 1H, NH); ^13^C NMR (CDCl_3_) δ = 28.42 ((CH_3_)_3_), 38.65 (C*Me*_3_), 44.97 (N(CH_2_)_2_), 49.98 (N(CH_2_)_2_), 116.95 (d, *J* = 23.6 Hz, C-3′, C-5′), 117.01 (C-3″, C-5″), 117.80 (C-3), 121.15 (d, *J* = 8.3 Hz, C-2′, C-6′), 121.69 (C-2″, C-6″), 123.97 (C-5), 124.03 (C-1), 131.20 (C-1″), 132.49 (C-6), 132.98 (C-4), 147.98 (C-4″), 151.06 (d, *J* = 2.6 Hz, C-1′), 155.43 (C-2), 159.65 (d, *J* = 244 Hz, C-4′), 162.35 (N(C=O)O), 176.37 ((C=O)NH); HRMS (ESI+): calcd for C_28_H_31_FN_3_O_3_^+^ [M + H]^+^: 476.2344; found: 476.2332.

*N*-{4-[4-(2,2-Dimethylpropanoyl)piperazin-1-yl]phenyl}-2-(4-fluorophenoxy)-3-nitrobenzamid (**44**): Reaction of the carboxylic acid **11** (140 mg (0.51 mmol)) with the amine **19** (133 mg (0.51 mmol)), 2-chloro-*N*-methylpyridinium iodide (227 mg (0.89 mmol)) and DIPEA (300 mg (2.55 mmol)) in dry dichloromethane (15 mL) gave the crude product. It was purified by column chromatography (silica gel, EtAc/CH 3:1) yielding compound **44** as orange amorphous solid (194 mg (73%)). IR = 3423, 1677, 1604, 1524, 1501, 1363, 1323, 1189, 836, 776; ^1^H NMR (CDCl_3_) δ = 1.31 (s, 9H, (CH_3_)_3_), 3.10–3.13 (m, 4H, N(CH_2_)_2_), 3.77–3.80 (m, 4H, N(CH_2_)_2_), 6.83–6.87 (m, 4H, 2′-H, 3″-H, 5″-H, 6′-H), 6.96–7.01 (m, 2H, 3′-H, 5′-H), 7.33 (d, *J* = 9.0 Hz, 2H, 2″-H, 6″-H), 7.55 (t, *J* = 8.0 Hz, 1H, 5-H), 8.08 (dd, *J* = 8.0, 1.8 Hz, 1H, 4-H), 8.44 (dd, *J* = 8.0, 1.8 Hz, 1H, 6-H), 8.66 (s, 1H, NH); ^13^C NMR (CDCl_3_) δ = 28.41 ((CH_3_)_3_), 38.66 (C*Me*_3_), 44.91 (N(CH_2_)_2_), 49.68 (N(CH_2_)_2_), 116.60 (d, *J* = 8.3 Hz, C-2′, C-6′), 116.81 (C-3″, C-5″), 116.82 (d, *J* = 23.8 Hz, C-3′, C-5′), 121.71 (C-2″, C-6″), 126.32 (C-5), 128.63 (C-4), 129.93 (C-1″), 131.12 (C-1), 136.58 (C-6), 143.84 (C-3), 144.97 (C-2), 148.52 (C-4″), 152.86 (d, *J* = 2.6 Hz, C-1′), 158.91 (d, *J* = 244 Hz, C-4′), 160.49 ((C=O)NH), 176.38 (C=O); HRMS (ESI+): calcd for C_28_H_30_FN_4_O_5_^+^ [M + H]^+^: 521.2195; found: 521.2183.

*N*-{4-[4-(2,2-Dimethylpropanoyl)piperazin-1-yl]phenyl}-3-fluoro-2-(4-fluorophenoxy)benzamid (**45**): Reaction of the carboxylic acid **10** (100 mg (0.40 mmol)) with the amine **19** (105 mg (0.40 mmol)), 2-chloro-*N*-methylpyridinium iodide (179 mg (0.70 mmol)) and DIPEA (259 mg (2.00 mmol) in dry dichloromethane (12 mL) gave the crude product. It was purified by column chromatography (flash silica gel, CH/CH_2_Cl_2_/EtAc 1:0.3:1.25) yielding compound **45** as pale yellow amorphous solid (122 mg (63%)). IR = 3405, 1623, 1502, 1462, 1417, 1320, 1270, 1232, 1188, 1016, 828, 767; ^1^H NMR (CDCl_3_) δ = 1.31 (s, 9H, (CH_3_)_3_), 3.09–3.12 (m, 4H, N(CH_2_)_2_), 3.77–3.81 (m, 4H, N(CH_2_)_2_), 6.88 (d, *J* = 9.0 Hz, 2H, 3″-H, 5″-H), 6.93–6.97 (m, 2H, 2′-H, 6′-H), 6.99–7.04 (m, 2H, 3′-H, 5′-H), 7.30–7.38 (m, 2H, 4-H, 5-H), 7.42 (d, *J* = 9.0 Hz, 2H, 2″-H, 6″-H), 8.04–8.07 (m, 1H, 6-H), 9.03 (s, 1H, NH); ^13^C NMR (CDCl_3_) δ = 28.41 ((CH_3_)_3_), 38.65 (C*Me*_3_), 44.93 (N(CH_2_)_2_), 49.84 (N(CH_2_)_2_), 116.61 (d, *J* = 23.8 Hz, C-3′, C-5′), 116.72 (d, *J* = 8.3 Hz, C-2′, C-6′), 116.93 (C-3″, C-5″), 120.29 (d, *J* = 18.6 Hz, C-4), 121.55 (C-2″, C-6″), 126.27 (d, *J* = 7.7 Hz, C-5), 127.10 (d, *J* = 3.3 Hz, C-6), 129.32 (C-1), 130.63 (C-1″), 140.40 (d, *J* = 13.1 Hz, C-2), 148.22 (C-4″), 152.96 (t, *J* = 2.2 Hz, C-1′), 154.98 (d, *J* = 252 Hz, C-3), 158.85 (d, *J* = 243 Hz, C-4′), 161.17 (d, *J* = 3.2 Hz, (C=O)NH), 176.36 (C=O); HRMS (ESI+): calcd for C_28_H_30_F_2_N_3_O_3_^+^ [M + H]^+^: 494.2250; found: 494.2234.

### 3.3. Biological Tests

#### 3.3.1. In Vitro Microplate Assay against *P. falciparum* NF54

The in vitro activity of compounds against erythrocytic stages of the drug sensitive NF54 strain of *P. falciparum* originating from Thailand was determined using a ^3^H-hypoxanthine incorporation assay [35,36,37]. Compounds were dissolved in DMSO at 10 mg/mL and further diluted in medium before adding to parasite cultures that were incubated in RPMI 1640 medium without hypoxanthine, supplemented with HEPES (5.94 g/L), NaHCO_3_ (2.1 g/L), neomycin (100 U/mL), AlbumaxR (5 g/L) and washed human red blood cells A+ at 2.5% haematocrit (0.3% parasitaemia). Serial drug dilutions of eleven 3-fold dilutions steps covering a range from 100 to 0.002 µg/mL were prepared. The 96-well plates were incubated in a humidified atmosphere at 37 °C; 4% CO_2_, 3% O_2_, 93% N_2_. After 48 h of incubation time, 0.05 mL of ^3^H-hypoxanthine (=0.5 µCi) was added to each well of the plate. The plates were incubated for further 24 h under the same conditions. Plates were then harvested using a Betaplate^TM^ cell harvester (Wallac, Zurich, Switzerland). Red blood cells were transferred onto a glass fiber filter and then washed with distilled water. The dried filters were inserted into a plastic foil with 10 mL of scintillation fluid and counted in a Betaplate^TM^ liquid scintillation counter (Wallac, Zurich, Switzerland). IC_50_ values were calculated from sigmoidal inhibition curves by linear regression using Microsoft Excel [38]. Chloroquine (Sigma C6628) was used as control.

#### 3.3.2. In Vitro Cytotoxicity with L-6 Cells

The cytotoxicity assays were performed using 96-well microtiter plates, each well containing 4000 L-6 cells (a primary cell line derived from rat skeletal myofibroblasts, ATCC CRL-1458^TM^) in 0.1 mL of RPMI 1640 medium supplemented with 1% glutamine (200 mM) and 10% fetal bovine serum [39,40]. Serial drug dilutions of eleven 3-fold dilution steps covering a range from 100 to 0.002 µg/mL were prepared. After 70 h of incubation, the plates were inspected under an inverted microscope to assure growth of the controls and sterile conditions. Then, 0.01 mL resazurin solution (resazurin, 12.5 mg in 100 mL double-distilled water) was added to each well and the plates were incubated for another 2 h. The plates were read with a Spectramax Gemini XS microplate fluorometer (Molecular Devices Cooperation, Sunnyvale, CA, USA) using an excitation wavelength of 536 nm and an emission wavelength of 588 nm. IC_50_ values were calculated by linear regression from the sigmoidal dose inhibition curves using SoftmaxPro software (Molecular Devices Cooperation, Sunnyvale, CA, USA) [38]. Podophyllotoxin (Sigma P4405) was used as control.

#### 3.3.3. Parallel Artificial Membrane Permeability Assay

With the high-throughput PAMPA the newly synthesized compounds were tested for their passive permeability through cell membranes without the influence of efflux pumps or transporter proteins. The assay was performed using a Corning^®^ Gentest^TM^ Pre-coated PAMPA Plate System with 96-well polystyrene plates. The bottom of the acceptor plate consists of a porous membrane, whereby the pores are lined with a lipid-oil-lipid triple layer. Stock solutions of each test compound at 10 mM were prepared in DMSO or methanol and diluted with phosphate-buffered saline (PBS at a pH of 7.4) to a final concentration of 200 µM. Hydrochlorothiazide (*Pe* = 0.9 nm/s) and caffeine (*Pe* = 80 nm/s) were used as standards. The donor plate (bottom plate) was filled with the compound solutions, whereby all compounds were tested in quadruplicates. Each well of the acceptor plate (top plate) was filled with PBS buffer. Donor and acceptor plates were combined and incubated at room temperature for 5 h. After that, the plates were separated and 150 µL of each well of both plates were transferred to 96-well UV plates (Greiner Bio-One). Absorption at different wavelengths covering a range from 200 to 300 nm was measured using a SpectraMax M3 UV plate reader. By measuring serial dilutions of five dilution steps covering a range from 200 to 12.5 µM, a calibration curve was prepared. The plates were analyzed at the wavelength where the R^2^ value of the calibration curve was higher than 0.99 [41]. Effective permeability *Pe* of each test compound was calculated using the following Equations (1)–(3):

(1) $$Pe=\frac{-\ln\left[1-\frac{c_{A}\left(t\right)}{c_{equ}}\right]}{S*\left(\frac{1}{V_{D}}+\frac{1}{V_{A}}\right)*t}$$

where:

*Pe*—effective permeability;
*S*—filter area (0.3 cm^2^);
*V_D_*—donor well volume (0.3 mL);
*V_A_*—acceptor well volume (0.2 mL);
*t*—incubation time (18,000 s);
*c_A_*(*t*)*—*acceptor well compound concentration at time *t*;
*c_equ_*—equilibrium concentration.

(2) $$c_{equ}=\frac{\left[c_{D}\left(t\right)*V_{D}+c_{A}\left(t\right)*V_{A}\right]}{\left(V_{D}+V_{A}\right)}$$

where:

*V_D_*—donor well volume (0.3 mL);
*V_A_*—acceptor well volume (0.2 mL);
*c_A_*(*t*)*—*acceptor well compound concentration at time *t*;
*c_D_*(*t*)*—*donor well compound concentration at time *t*.

Recovery of compounds from donor and acceptor wells (mass retention) was calculated as shown in the equation below. Data were only accepted when recovery exceeded 70%.

(3) $$R=1-\frac{\left[c_{D}\left(t\right)*V_{D}+c_{A}\left(t\right)*V_{A}\right]}{\left(c_{0}*V_{D}\right)}$$

where:

*R*—mass retention (%);
*V_D_*—donor well volume (0.3 mL);
*V_A_*—acceptor well volume (0.2 mL);
*c_A_*(*t*)*—*acceptor well compound concentration at time *t*;
*c_D_*(*t*)*—*donor well compound concentration at time *t*;
*c*_0_—initial donor well compound concentration (200 µM).

#### 3.3.4. Ligand Efficiency (LE)

Ligand efficiency was calculated as shown in the following Equation (4):

(4) $$LE=\frac{1.37}{HA}*pIC_{50}$$

where:

*LE*—ligand efficiency;
*HA*—number of heavy atoms;
*pIC*_50_—negative logarithm of IC_50_.

## 4. Conclusions

**Figure 6:**
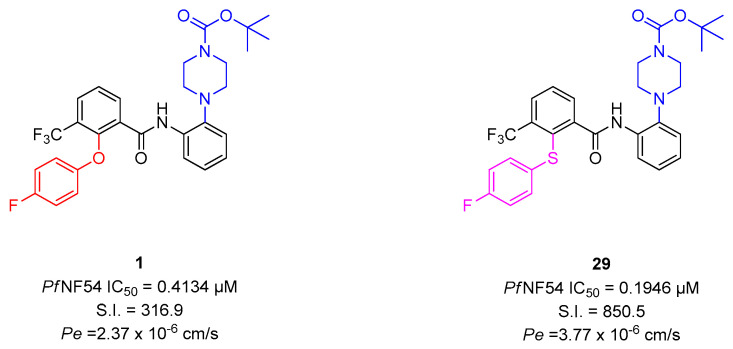
Structure−activity relationships of compounds **1** and **29**.

This paper deals with the synthesis of derivates of MMV’s Malaria Box compound **1**, which exhibits multi-stage activity against different strains of *P. falciparum* and lack of resistance development. It is a 2-(4-fluorophenoxy)-3-(trifluoromethyl)benzanilide with a *N*-bocpiperazinyl group in *ortho* position of the anilide nitrogen. The first series focused on the derivatization of the anilino moiety showing the positive influence of a *N*-bocpiperazinyl group or a 4-pivaloylpiperazinyl group in *ortho* or *para* position, which became partial structure of all of the new compounds. The 3-(trifluoromethyl) group was replaced by hydrogen, fluoro, amino or nitrogen substituents, but turned out to be the preferable substitution. The 2-(4-fluorophenoxy) moiety was replaced by an anilino, a 4-fluoroanilino or a (4-fluorophenyl)sulfanyl substituent. The latter was partial structure of **29** which exhibits a *N*-bocpiperazinyl group in 2′-position of the benzanilide. Compared to **1** it showed improved activity, selectivity and passive permeability (Figure 6).

## Figures and Tables

**Table 1:** Activities of compounds **26**–**47** against *P. falciparum* NF54 and L-6 cells, expressed as IC50 (µM) ^a^.

Compound	*Pf*NF54 ^b^ IC_50_ (µM)	S.I. = IC_50_ (Cyt.)/IC_50_ (*Pf*NF54)	Cytotoxicity L-6 Cells IC_50_ (µM)
**1**	0.4134	316.9	131.0
**2**	0.2690	461.0	124.0
**3**	0.6172	299.7	185.0
**4**	0.5795	171.9	99.62
**26**	0.6266	245.5	153.8
**27**	0.6496	237.5	154.3
**28**	0.5908	153.9	90.39
**29**	0.1946	850.5	165.5
**30**	1.131	19.87	22.47
**31**	0.6142	209.6	128.8
**32**	0.6364	193.8	123.4
**33**	2.145	17.84	38.27
**34**	2.613	43.75	114.3
**35**	2.138	7.275	15.55
**36**	3.397	59.88	203.4
**37**	4.072	8.676	35.33
**38**	1.432	49.46	70.82
**39**	0.1494	212.8	31.79
**40**	3.094	31.78	98.30
**41**	0.6336	53.29	33.76
**42**	0.5389	174.8	94.17
**43**	5.615	32.84	184.4
**44**	1.498	96.41	144.5
**45**	1.793	60.90	109.2
**46**	1.010	126.0	127.3
**47**	3.083	51.38	158.4
CQ	0.009	9672	90.92
POD			0.012

CQ = chloroquine; POD = podophyllotoxin; ^a^ IC_50_ values represent the average of four determinations (two determinations of two independent experiments); ^b^ sensitive to chloroquine.

**Table 2:** Key physicochemical parameters and passive permeability values of compounds **26**–**47**.

Compound	log P/log D_7.4_ ^a^	LE (kcal/mol/HA)	*Pe* ^b^ (10^−6^ cm/s)
**1**	6.44	0.219	2.37
**2**	6.44	0.225	0.09
**3**	6.57	0.218	0.23
**4**	5.56	0.236	0.24
**26**	5.56	0.236	n.d.
**27**	5.71	0.229	n.d.
**28**	5.50	0.200	1.68
**29**	7.13	0.230	3.77
**30**	5.42	0.233	8.52
**31**	7.54	0.218	0.65
**32**	7.68	0.212	0.57
**33**	5.69	0.222	n.d.
**34**	5.63	0.201	6.92
**35**	5.83	0.216	0.00
**36**	5.56	0.208	2.84
**37**	5.71	0.200	0.70
**38**	5.50	0.219	1.53
**39**	7.13	0.234	4.31
**40**	5.42	0.216	5.57
**41**	7.54	0.218	1.32
**42**	7.68	0.215	0.20
**43**	5.69	0.206	0.02
**44**	5.63	0.210	n.d.
**45**	5.83	0.219	5.13
**46**	4.73	0.222	1.44
**47**	4.73	0.204	0.62
Hydrochlorothiazide			0.09
Caffeine			8.00

^a^ log P and log D were calculated using the ChemAxon software JChem for Excel 14.9.1500.912 (2014) (Chemaxon, Budapest, Hungary); ^b^ determined by PAMPA, n.d. could not be determined.

## Data Availability

Data is contained within the article and supplementary material.

## Supplementary Materials

The following supporting information can be downloaded at: https://www.mdpi.com/article/10.3390/ph15121503/s1, Figures S1–S27: ^1^H- and ^13^C-NMR spectra for compounds **10**, **11**, **13**–**15** and **26**–**47**.
