# Supplementary material for: New Derivatives of the Multi-Stage Active Malaria Box Compound MMV030666 and Their Antiplasmodial Potencies
Source: Pharmaceuticals (Basel). 2022 Dec 2;15(12):1503. doi: 10.3390/ph15121503 (PMC9783227; doi:10.3390/ph15121503)

Supplementary Information

New Derivatives of the Multi-Stage Active Malaria Box Compound MMV030666 and Their Antiplasmodial Potencies

Theresa Hermann [1,*], Robin Wallner [1], Johanna Dolensky [1], Werner Seebacher [1], Eva-Maria Pferschy-Wenzig [2], Marcel Kaiser [3,4], Pascal Mäser [3,4] and Robert Weis [1]

[1] Pharmaceutical Chemistry, Institute of Pharmaceutical Sciences, University of Graz, Schubertstraße 1, 8010 Graz, Austria
[2] Pharmacognosy, Institute of Pharmaceutical Sciences, University of Graz, Beethovenstraße 8, 8010 Graz, Austria
[3] Swiss Tropical and Public Health Institute, Kreuzstraße 2, Allschwil, CH-4123 Basel, Switzerland
[4] Faculty of Philosophy and Natural Sciences, Swiss Tropical and Public Health Institute Petersplatz 1, University of Basel, CH-4003 Basel, Switzerland
* Correspondence: theresa.hermann@uni-graz.at; Tel.: +43-316-380-5381; Fax: +43-316-380-9846

NMR spectra data of compounds 10, 11, 13-15 and 26-47

Figure S1. 1H NMR at 400 MHz and 13C NMR at 100 MHz spectra for compound **10** ($CDCl_3$)

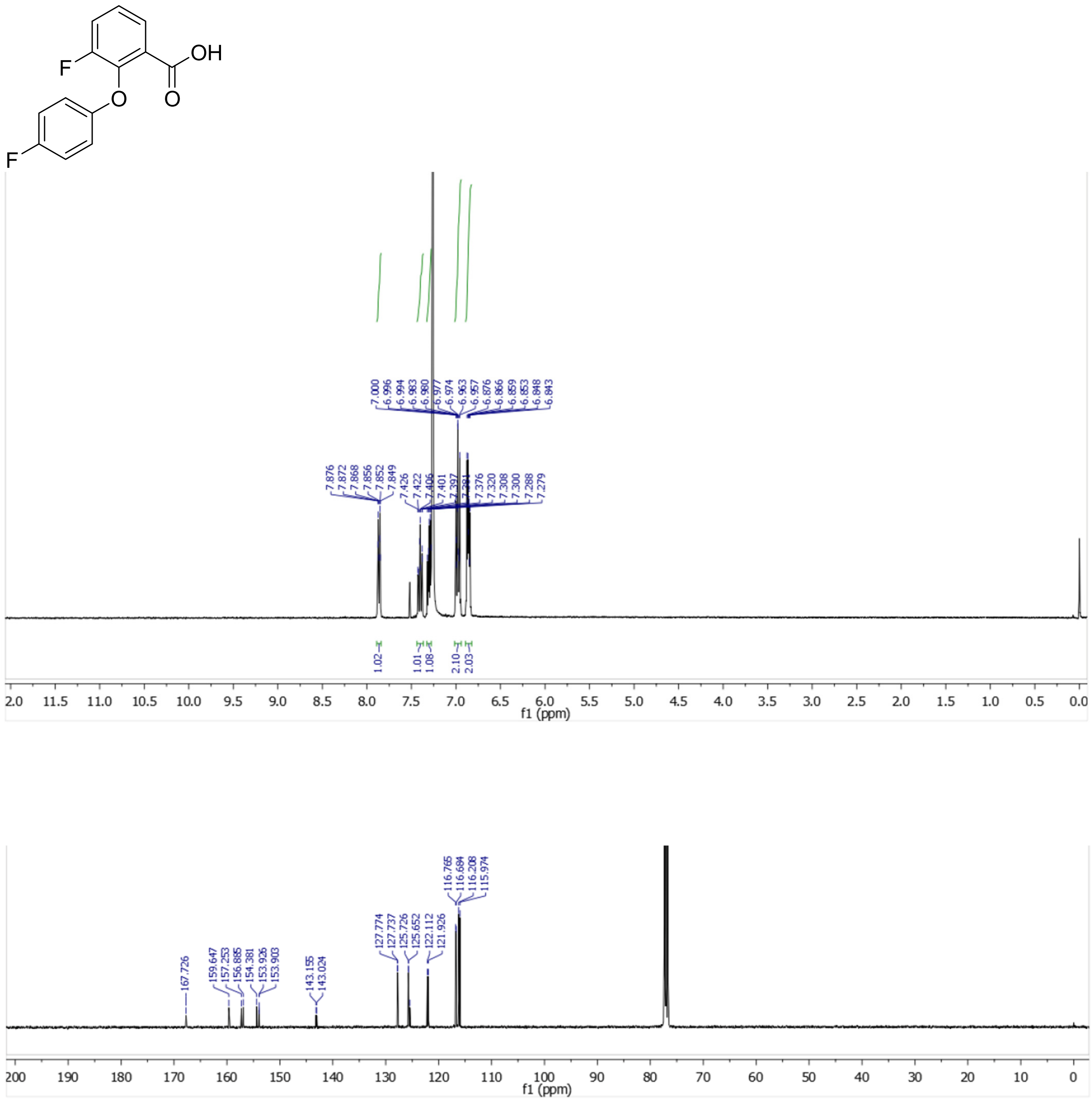

Figure S2. 1H NMR at 400 MHz and 13C NMR at 100 MHz spectra for compound **11** ($CDCl_3$)

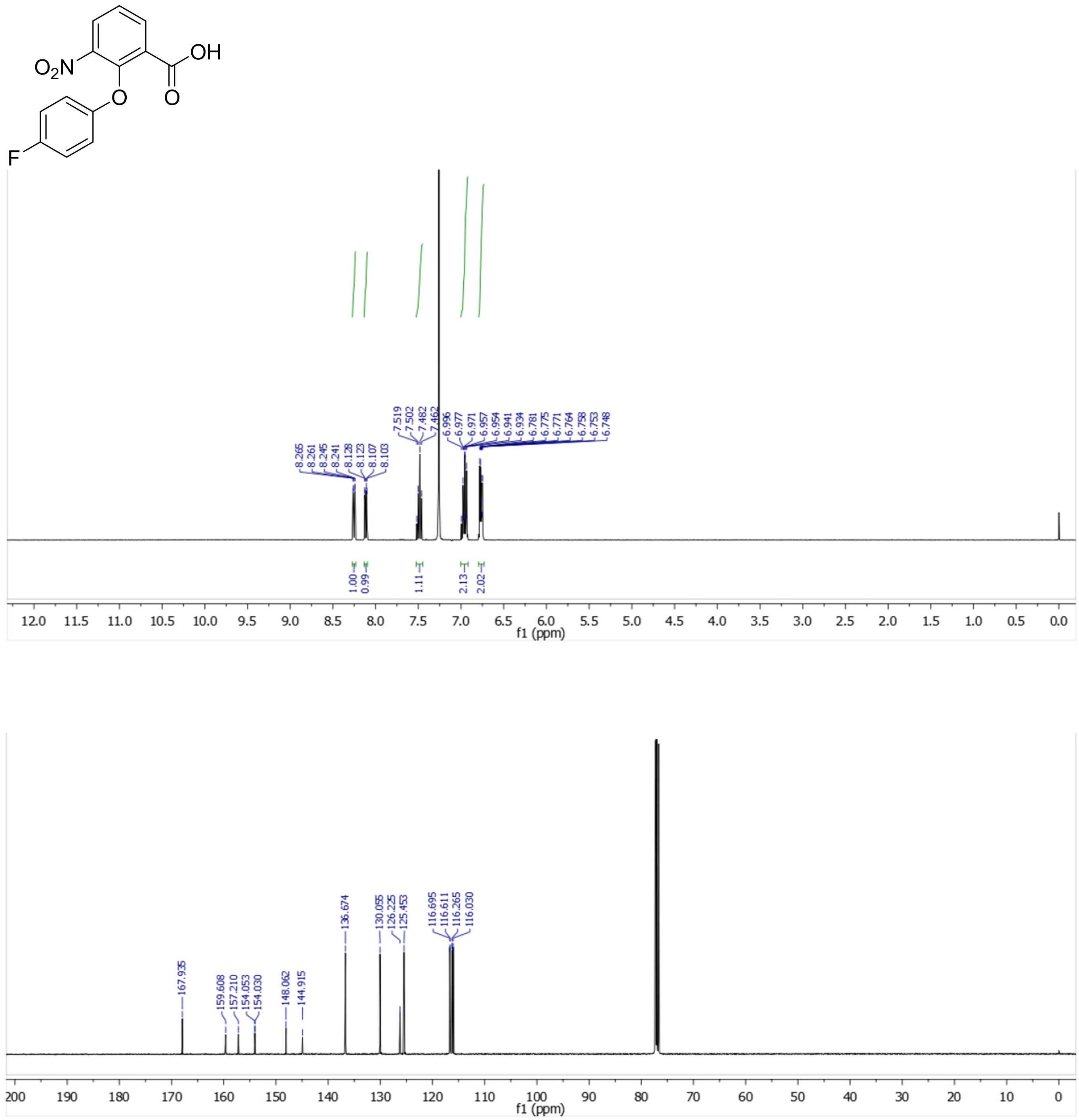

Figure S3. 1H NMR at 400 MHz and 13C NMR at 100 MHz spectra for compound **13** ($CDCl_3$)

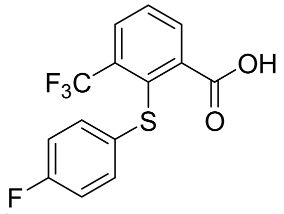

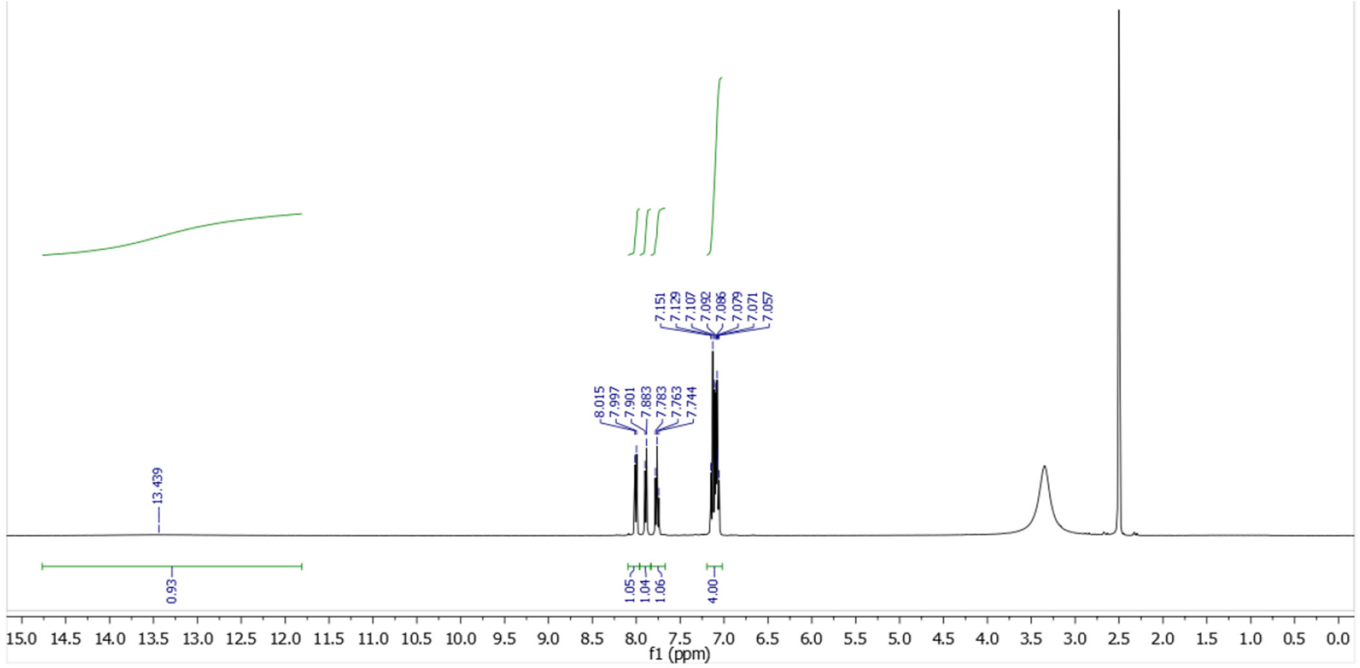

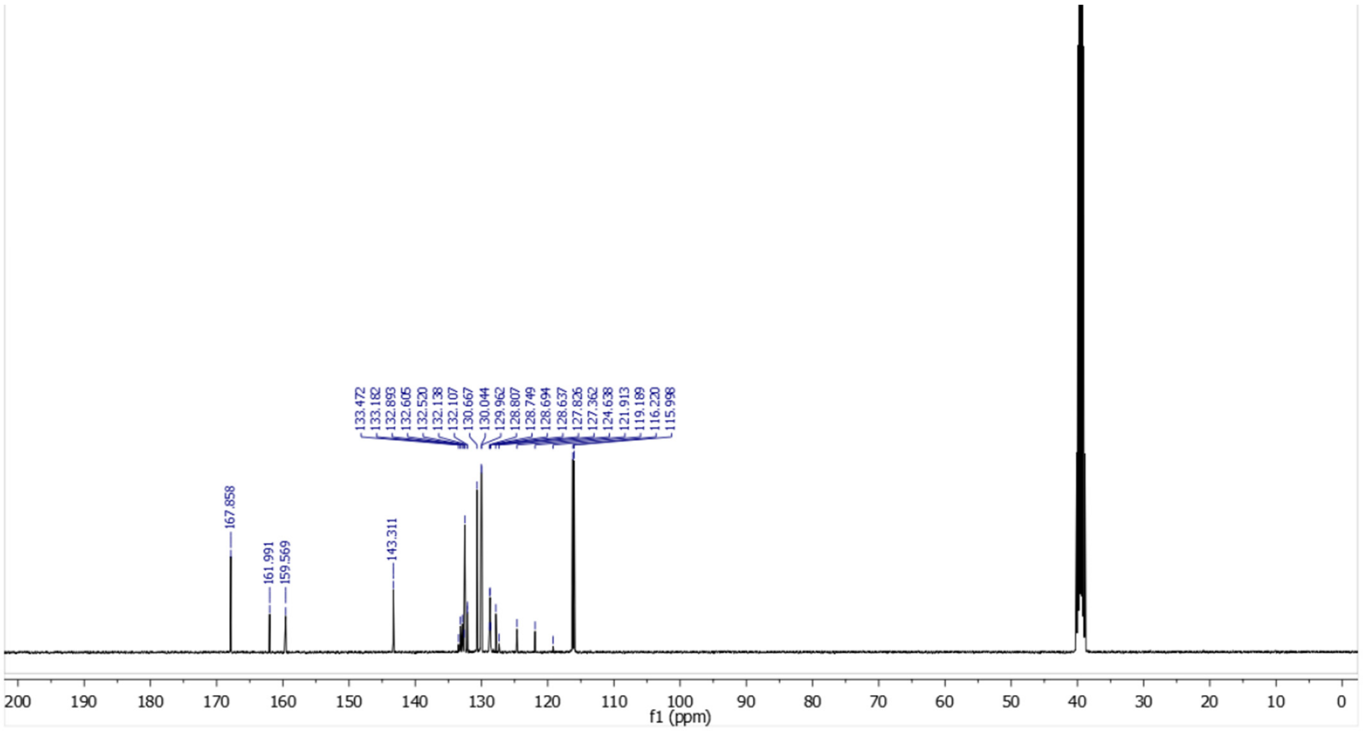

Figure S4. 1H NMR at 400 MHz and 13C NMR at 100 MHz spectra for compound **14** ($CDCl_3$)

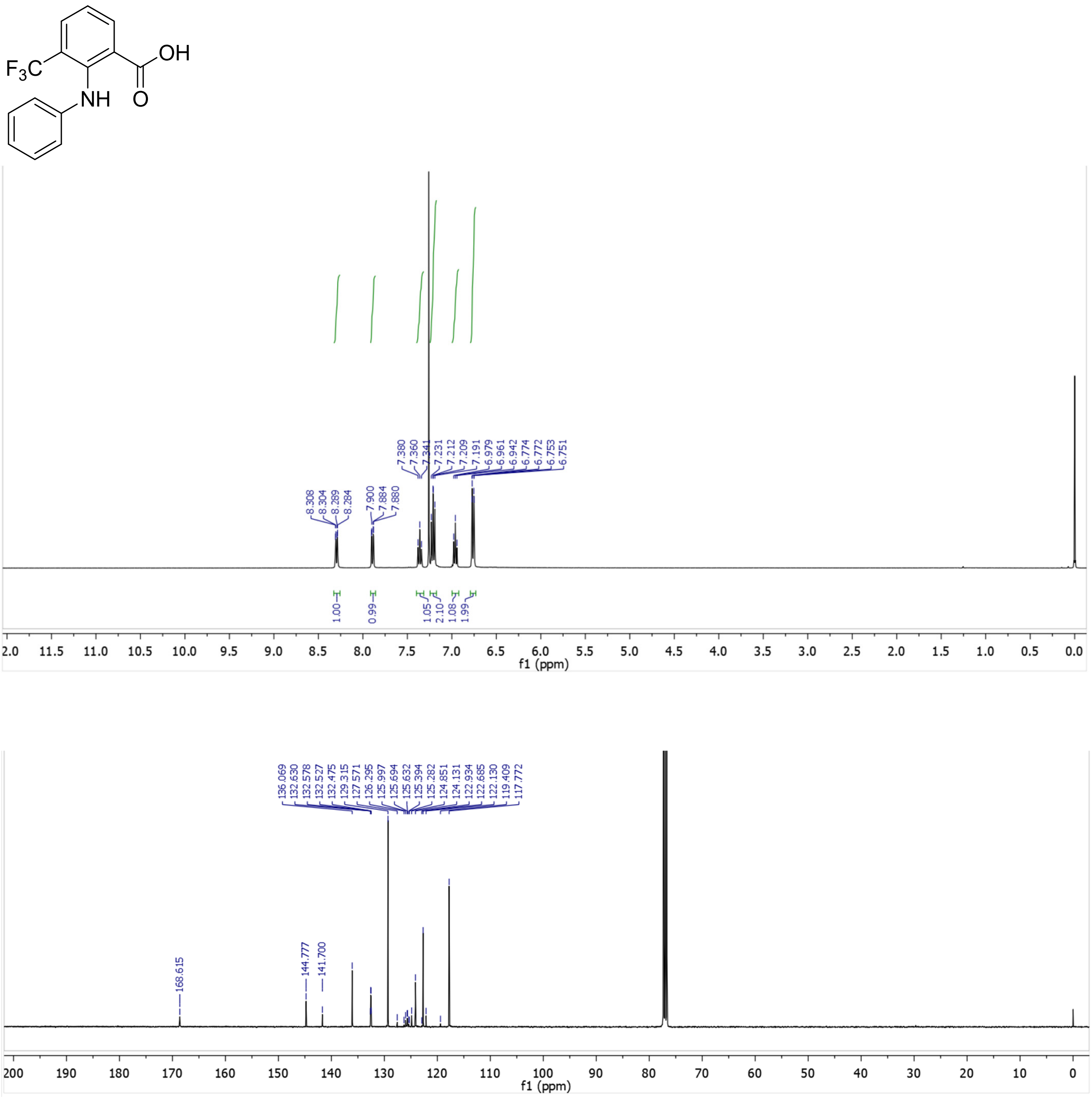

Figure S5. 1H NMR at 400 MHz and 13C NMR at 100 MHz spectra for compound **15** (CDCl3)

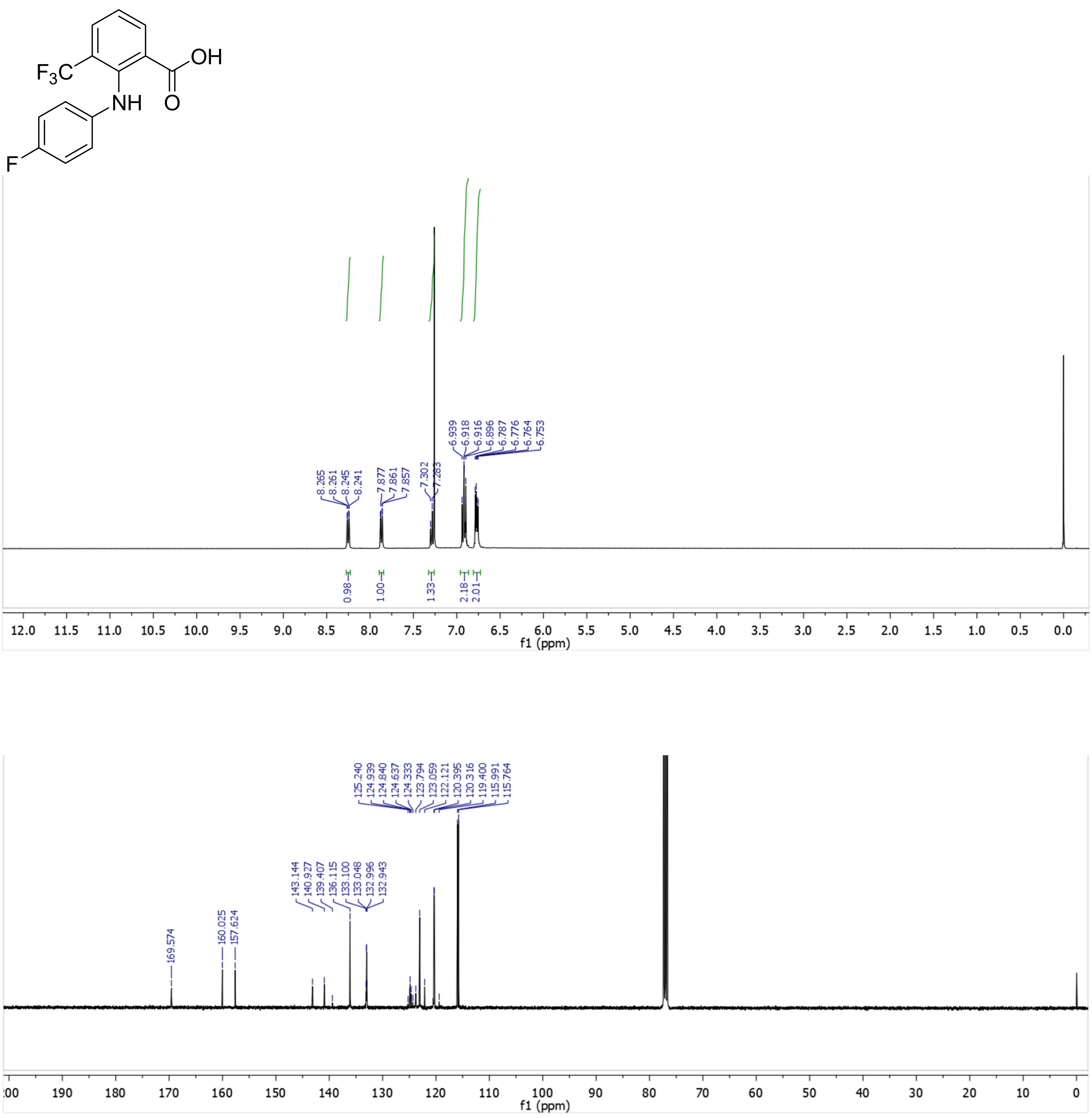

Figure S6. 1H NMR at 400 MHz and 13C NMR at 100 MHz spectra for compound **26** ($CDCl_3$)

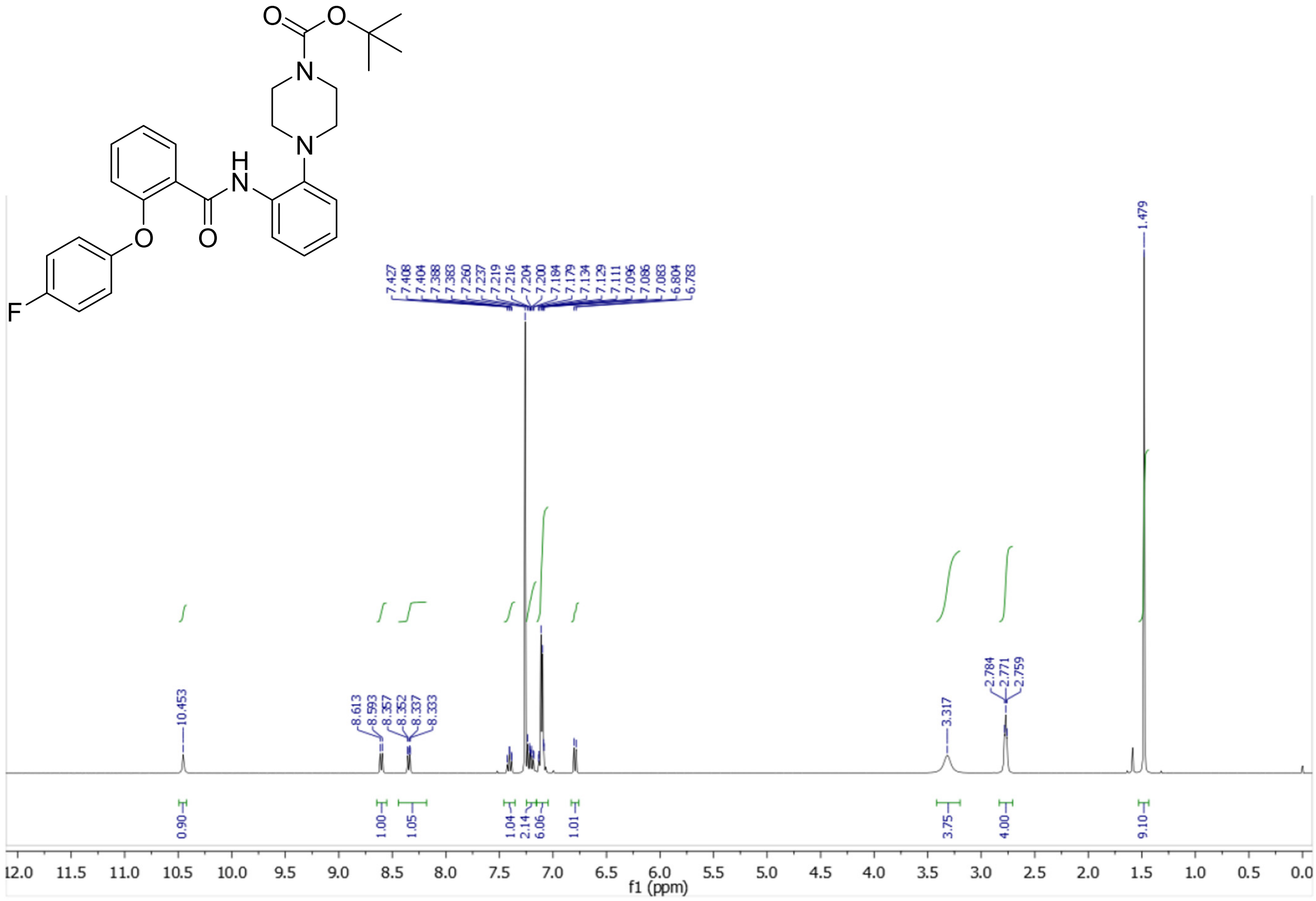

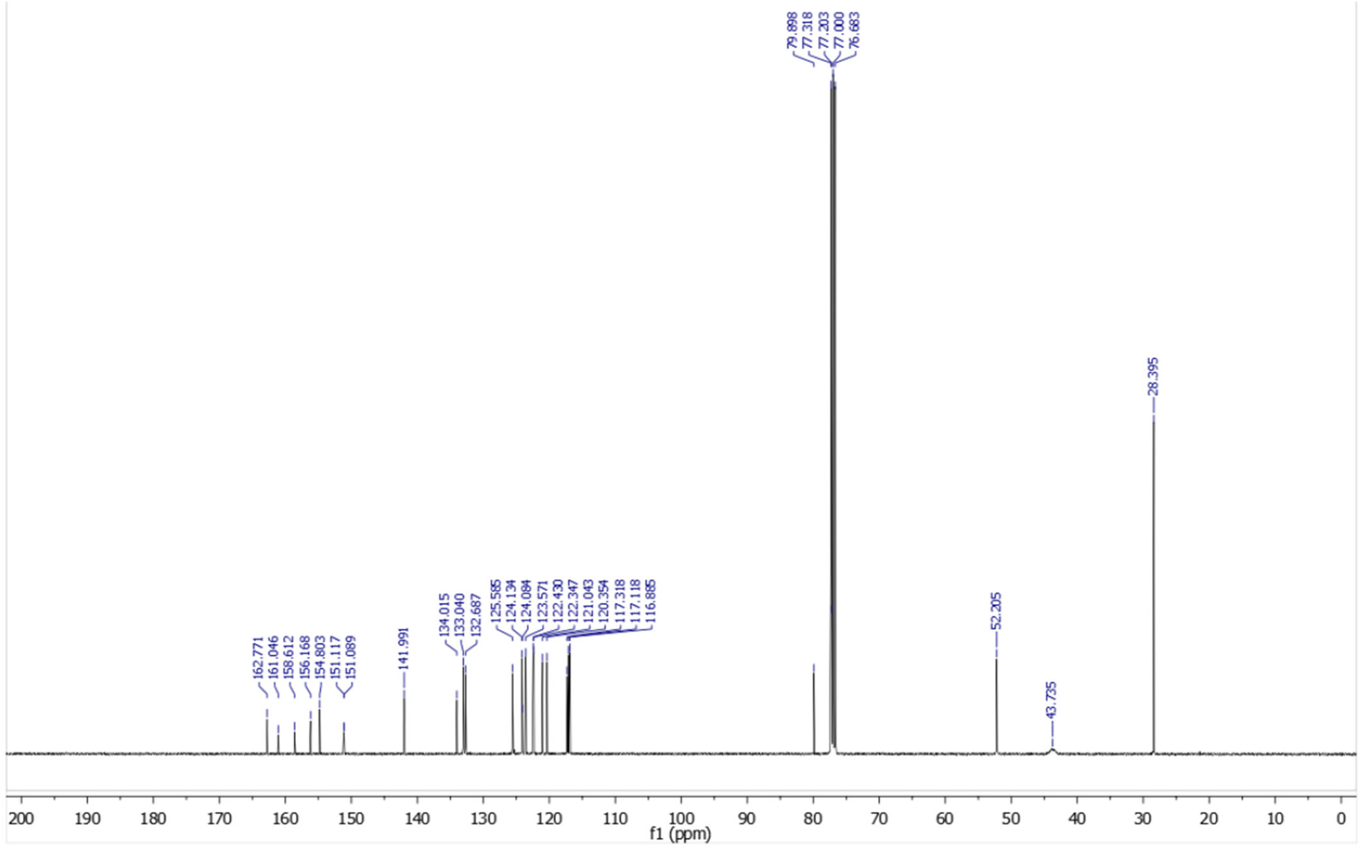

Figure S7. 1H NMR at 400 MHz and 13C NMR at 100 MHz spectra for compound **27** ($CDCl_3$)

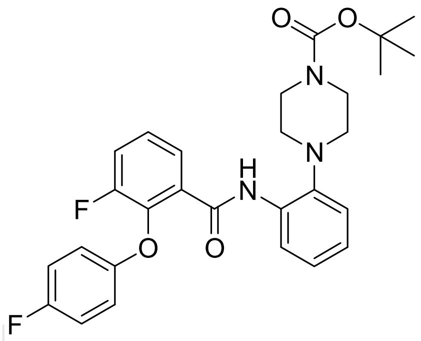

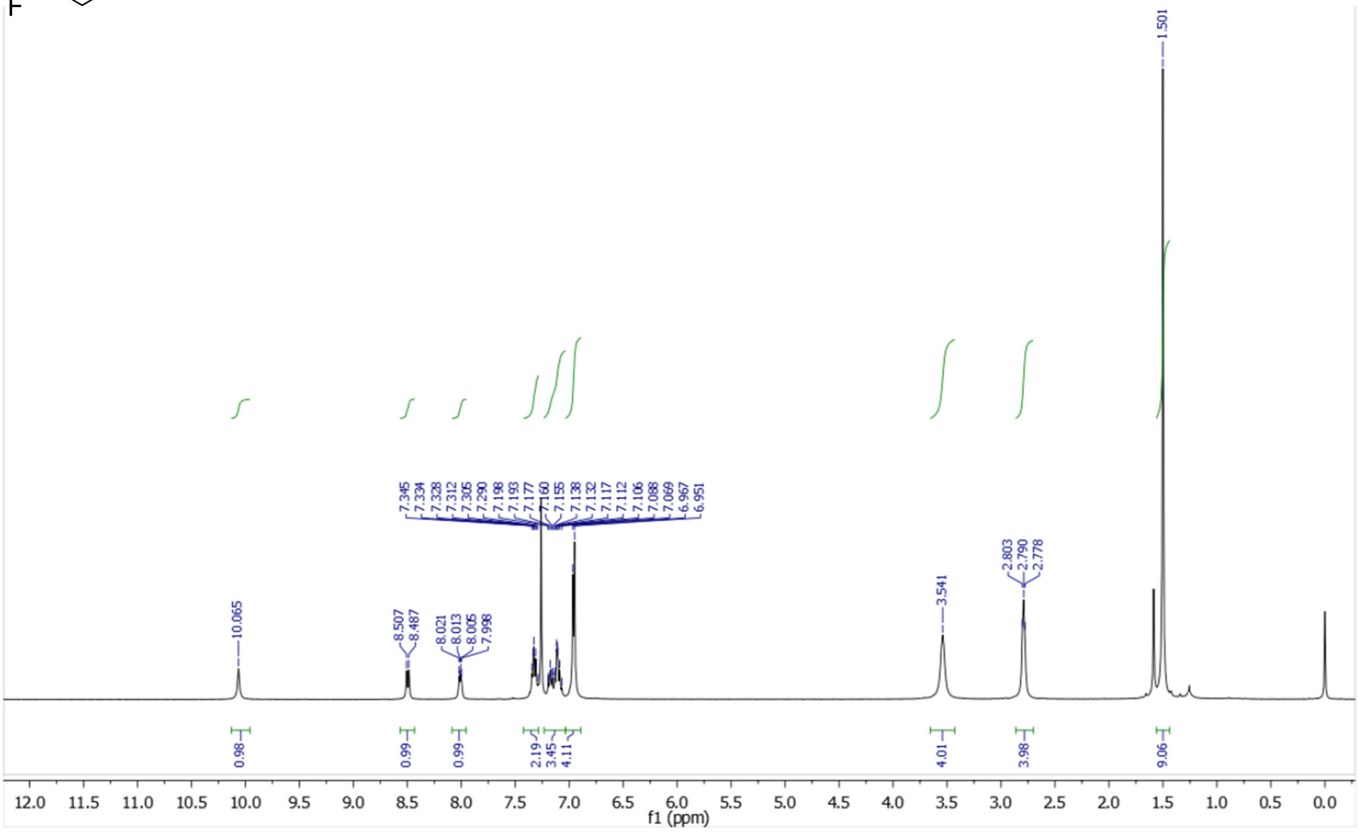

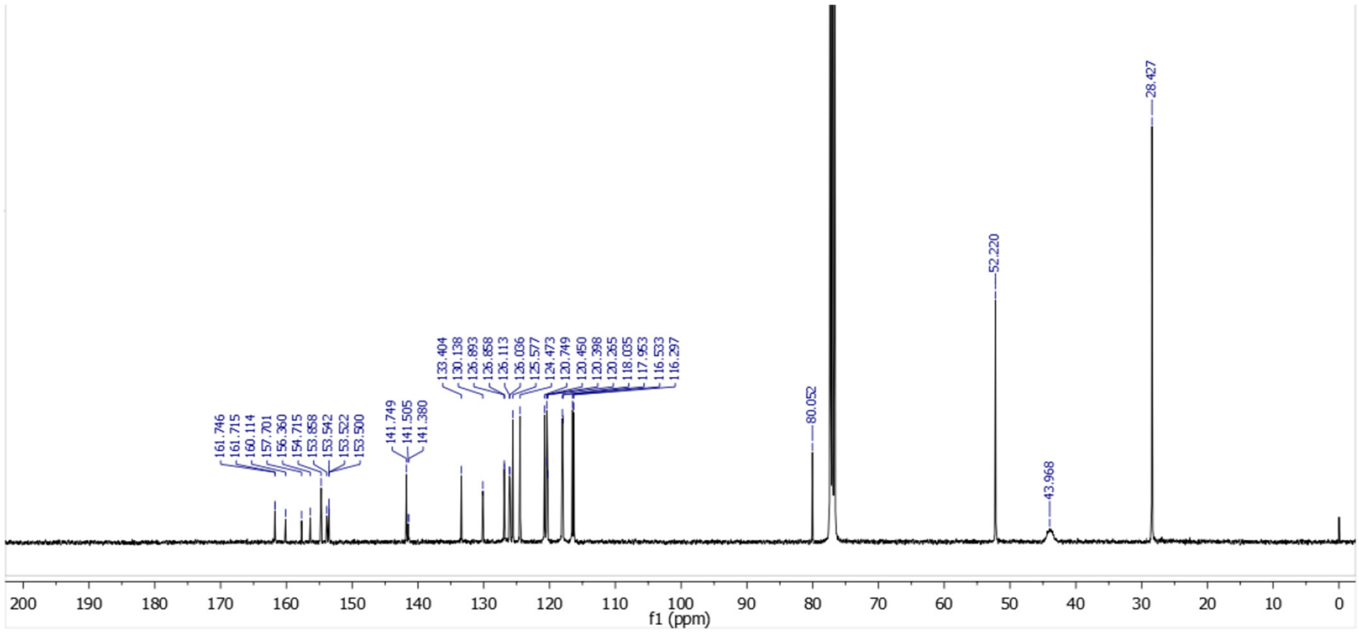

Figure S8. 1H NMR at 400 MHz and 13C NMR at 100 MHz spectra for compound **28** ($CDCl_3$)

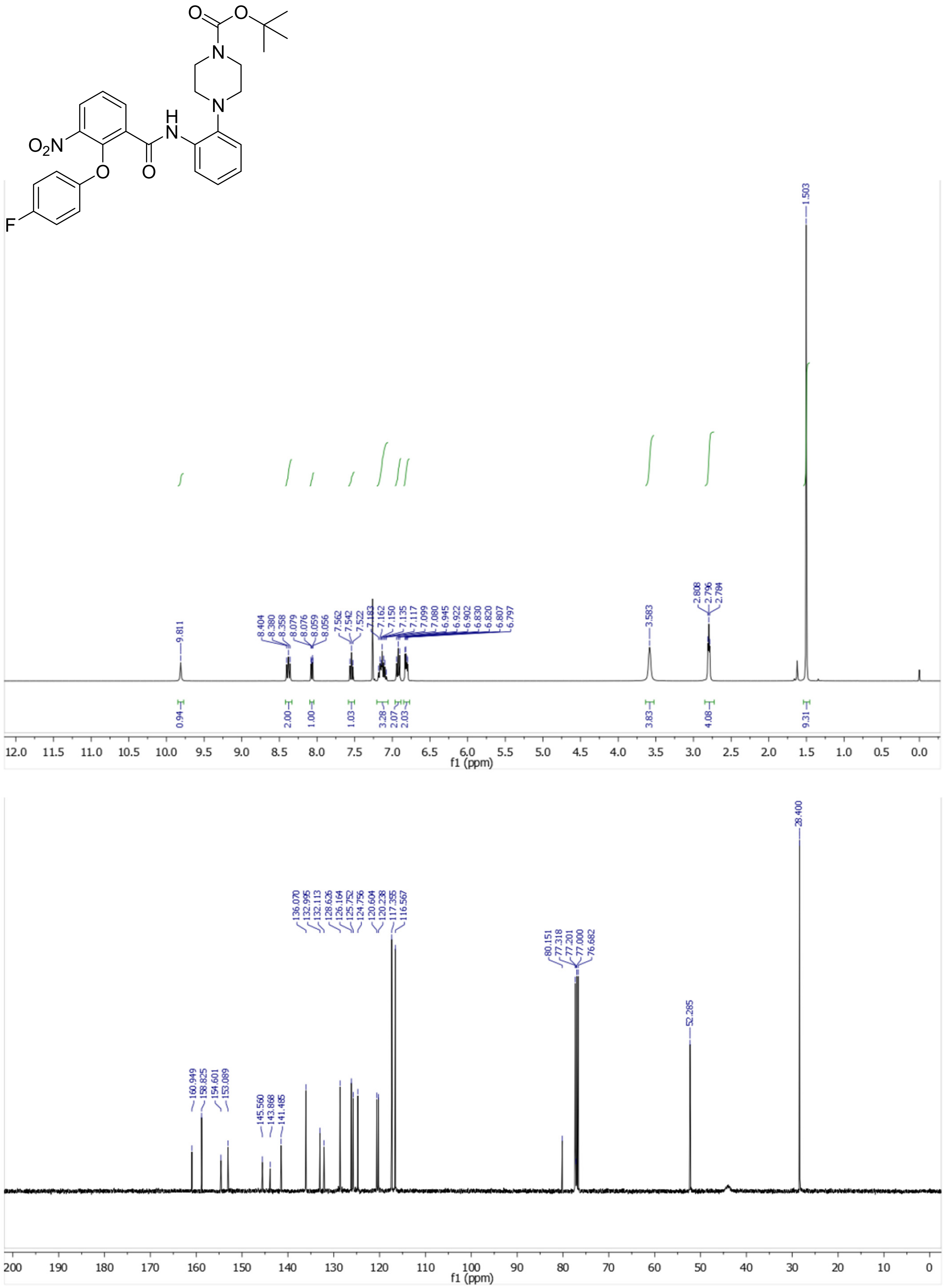

Figure S9. 1H NMR at 400 MHz and 13C NMR at 100 MHz spectra for compound **29** ($CDCl_3$)

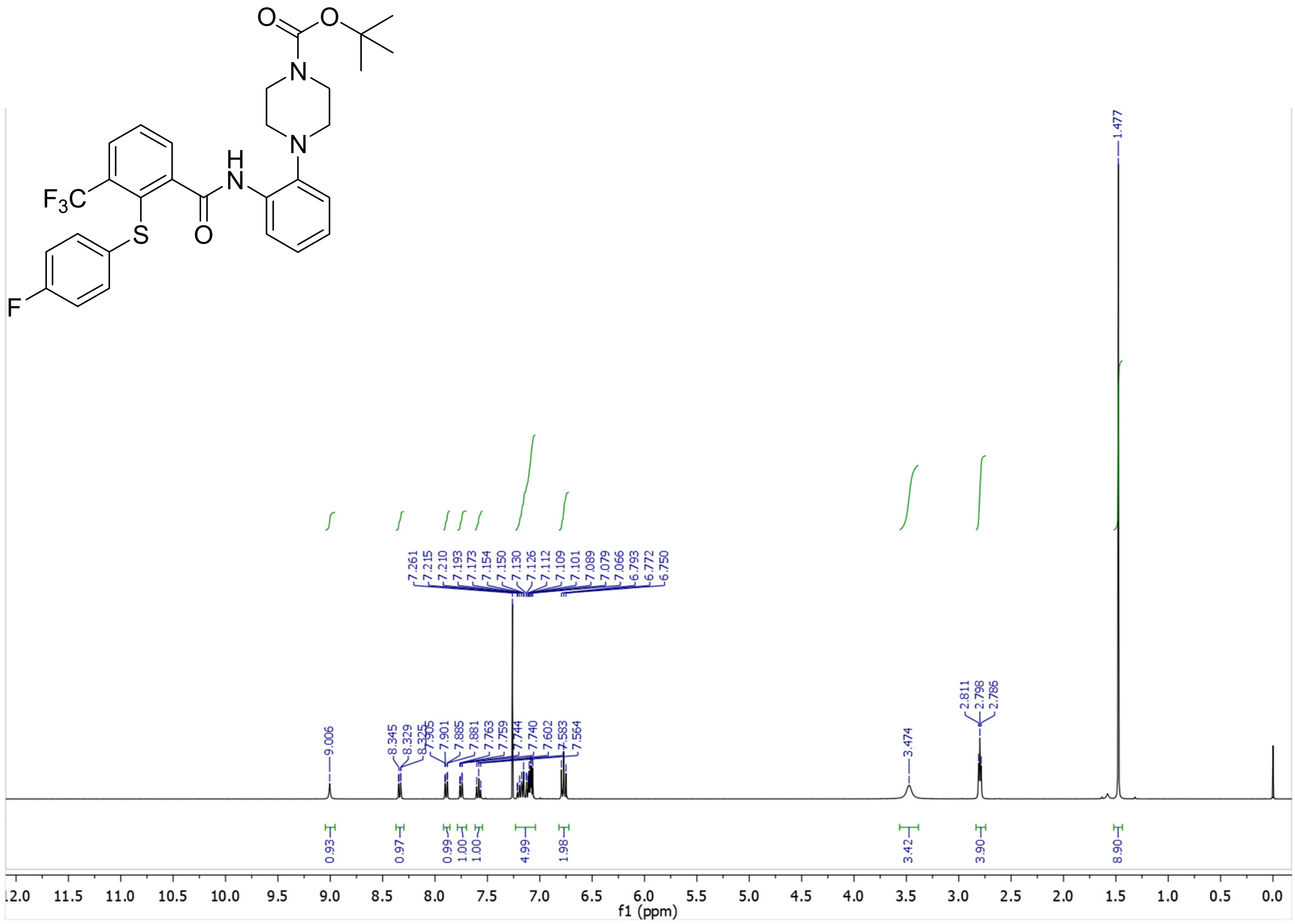

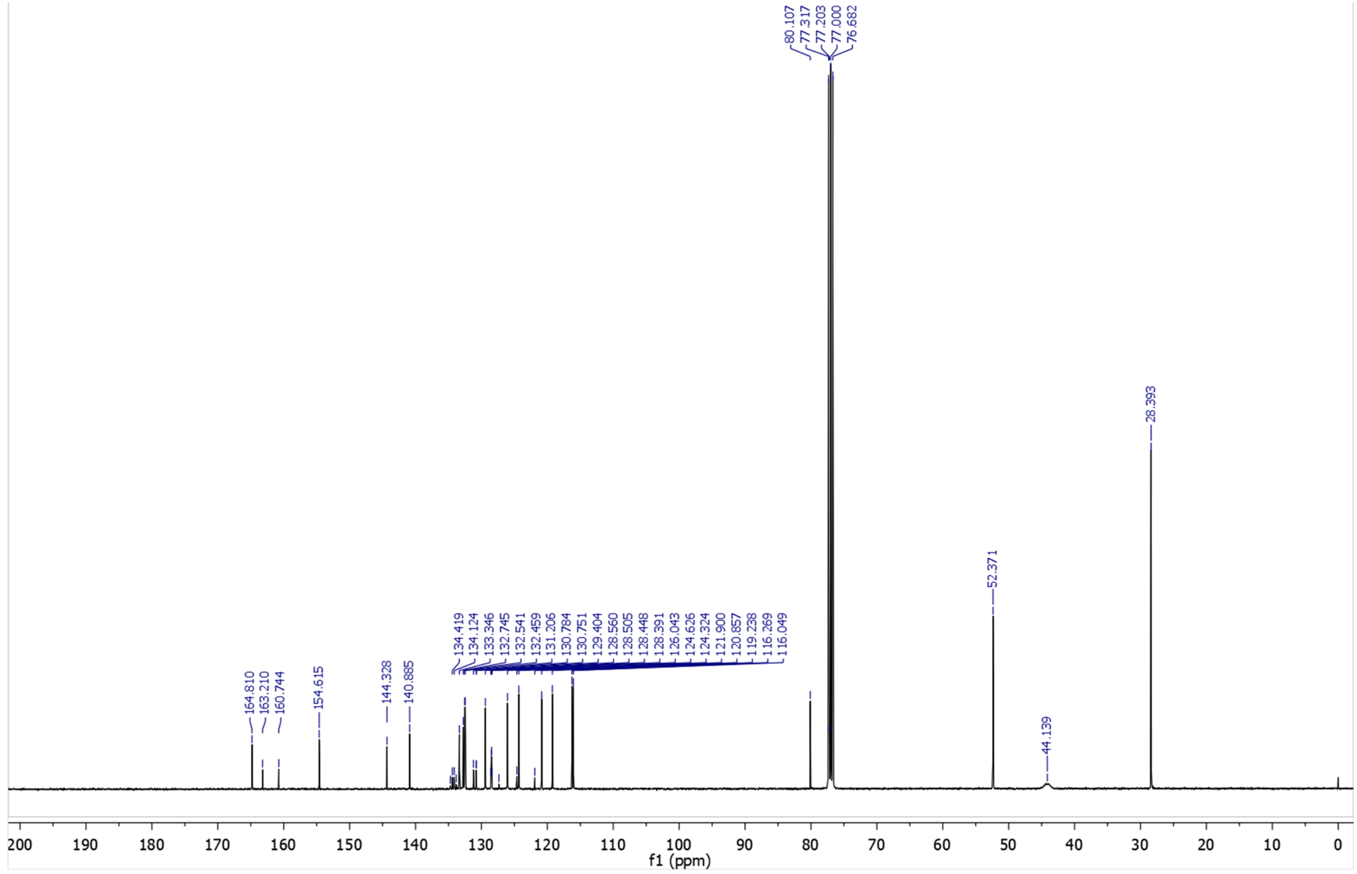

Figure S10. 1H NMR at 400 MHz and 13C NMR at 100 MHz spectra for compound **30** ($CDCl_3$)

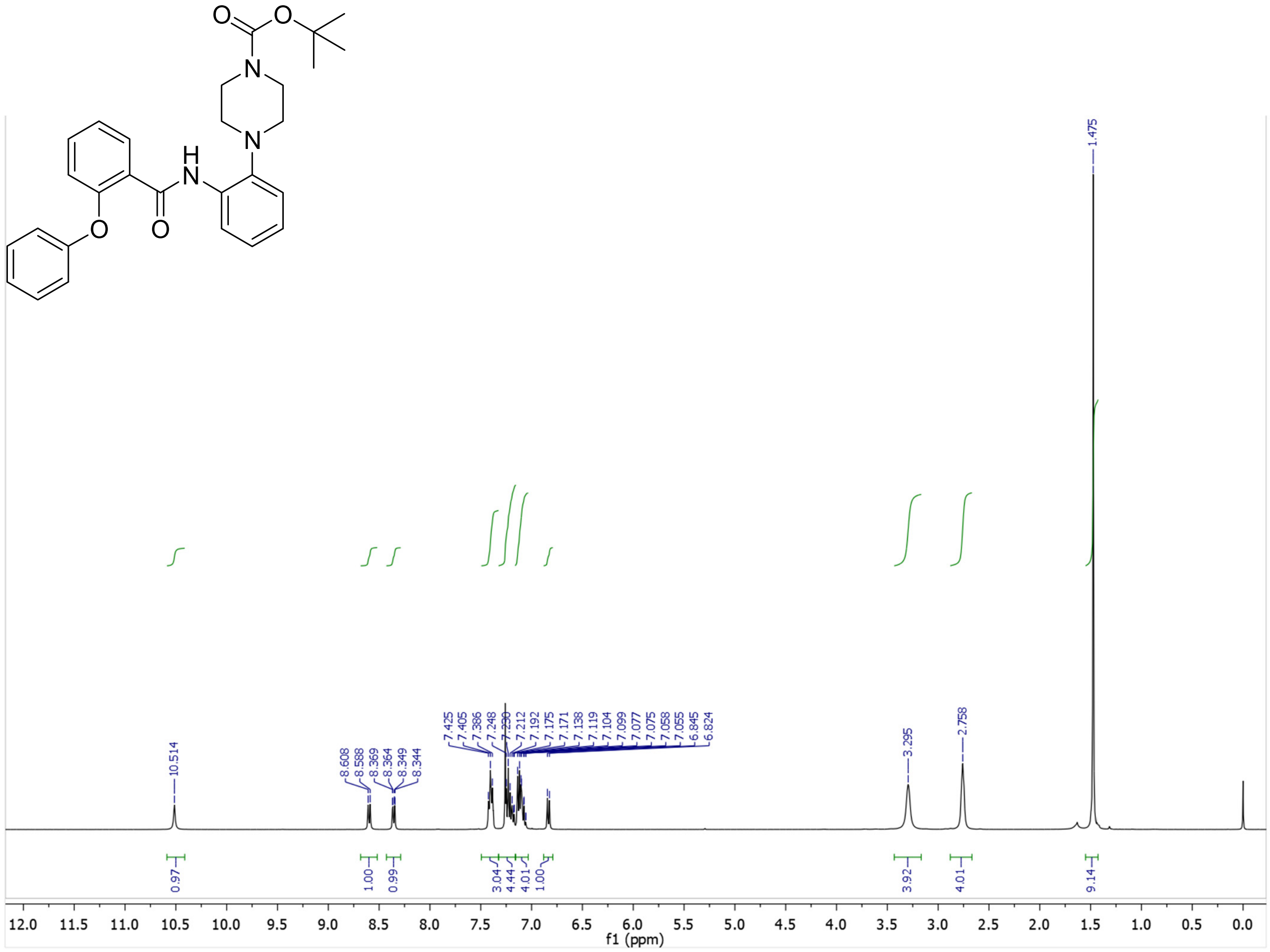

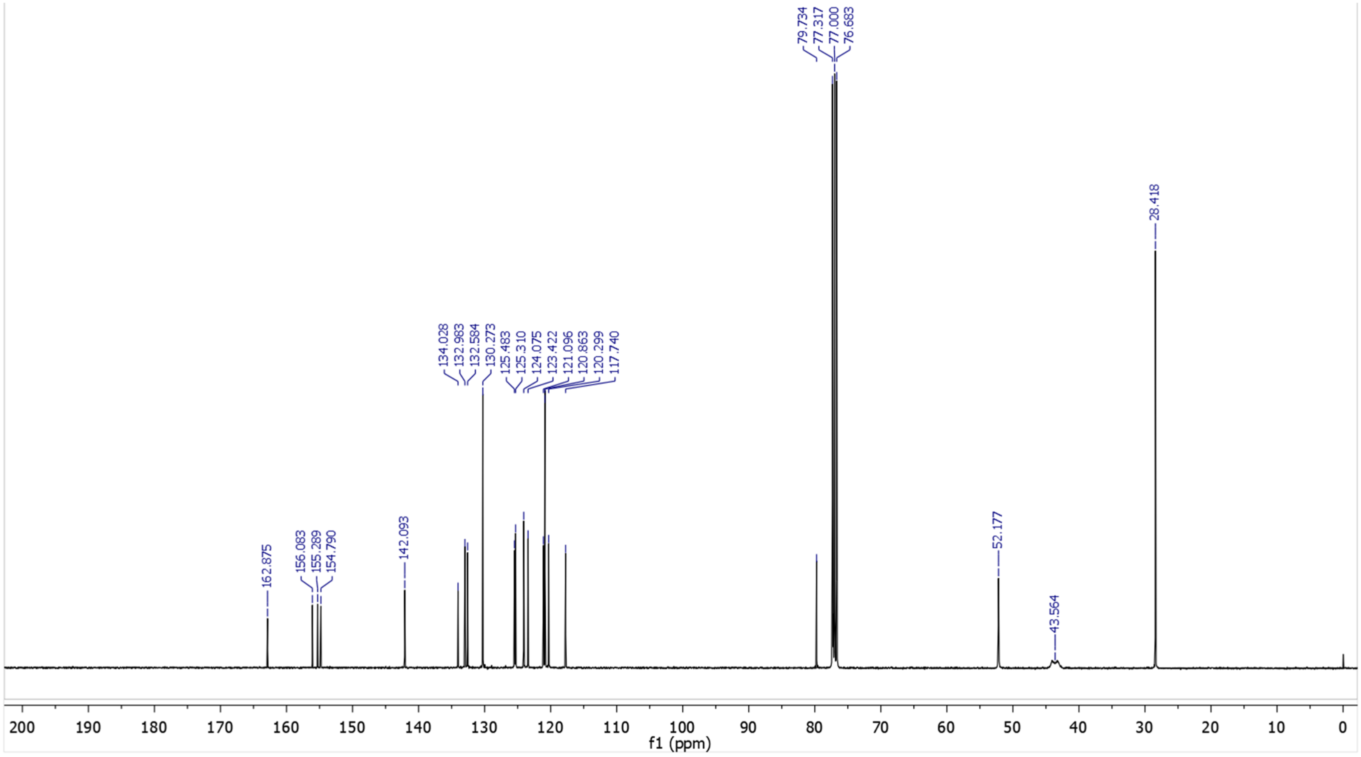

Figure S11. 1H NMR at 400 MHz and 13C NMR at 100 MHz spectra for compound **31** ($CDCl_3$)

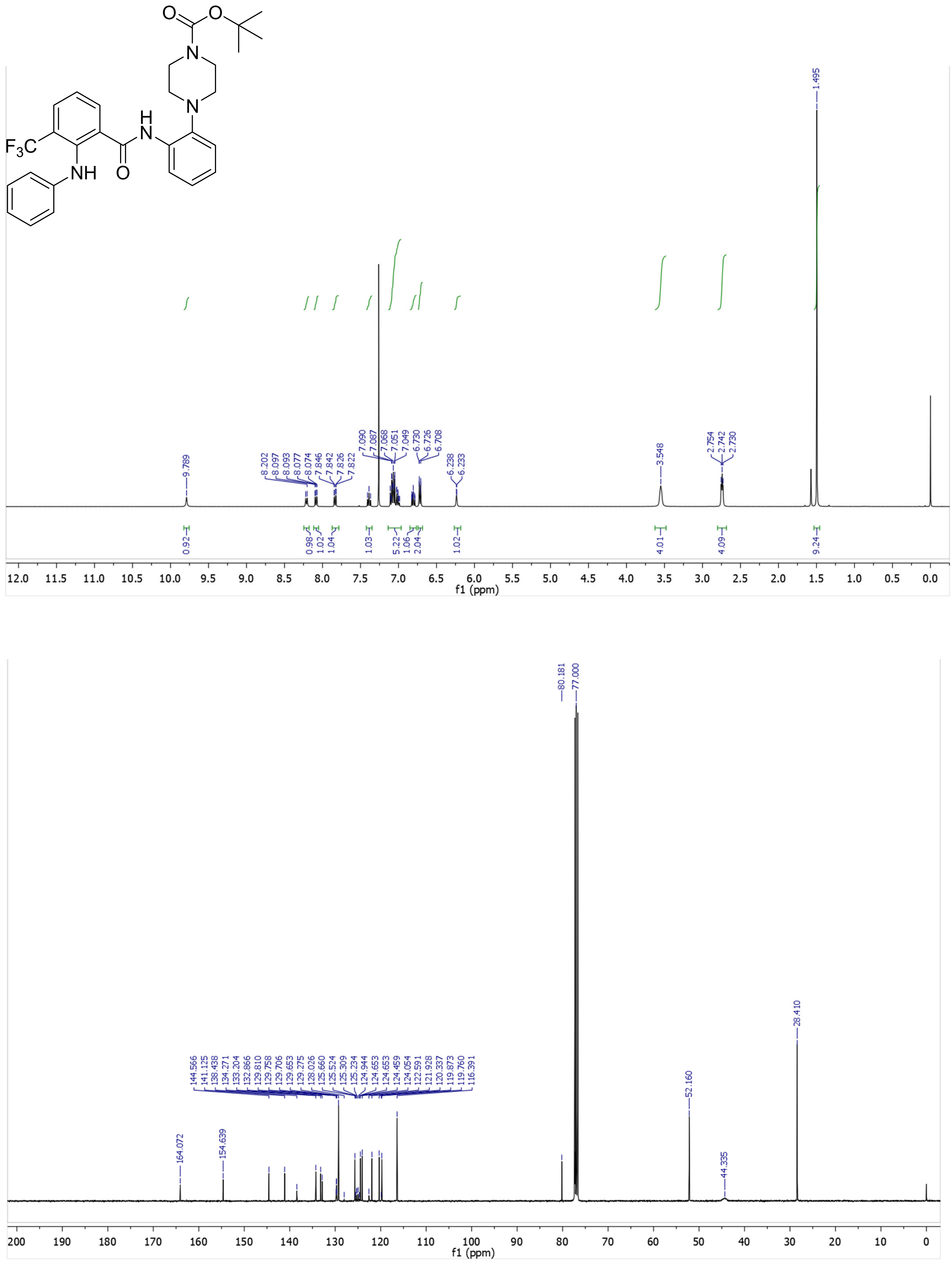

Figure S12. 1H NMR at 400 MHz and 13C NMR at 100 MHz spectra for compound **32** ($CDCl_3$)

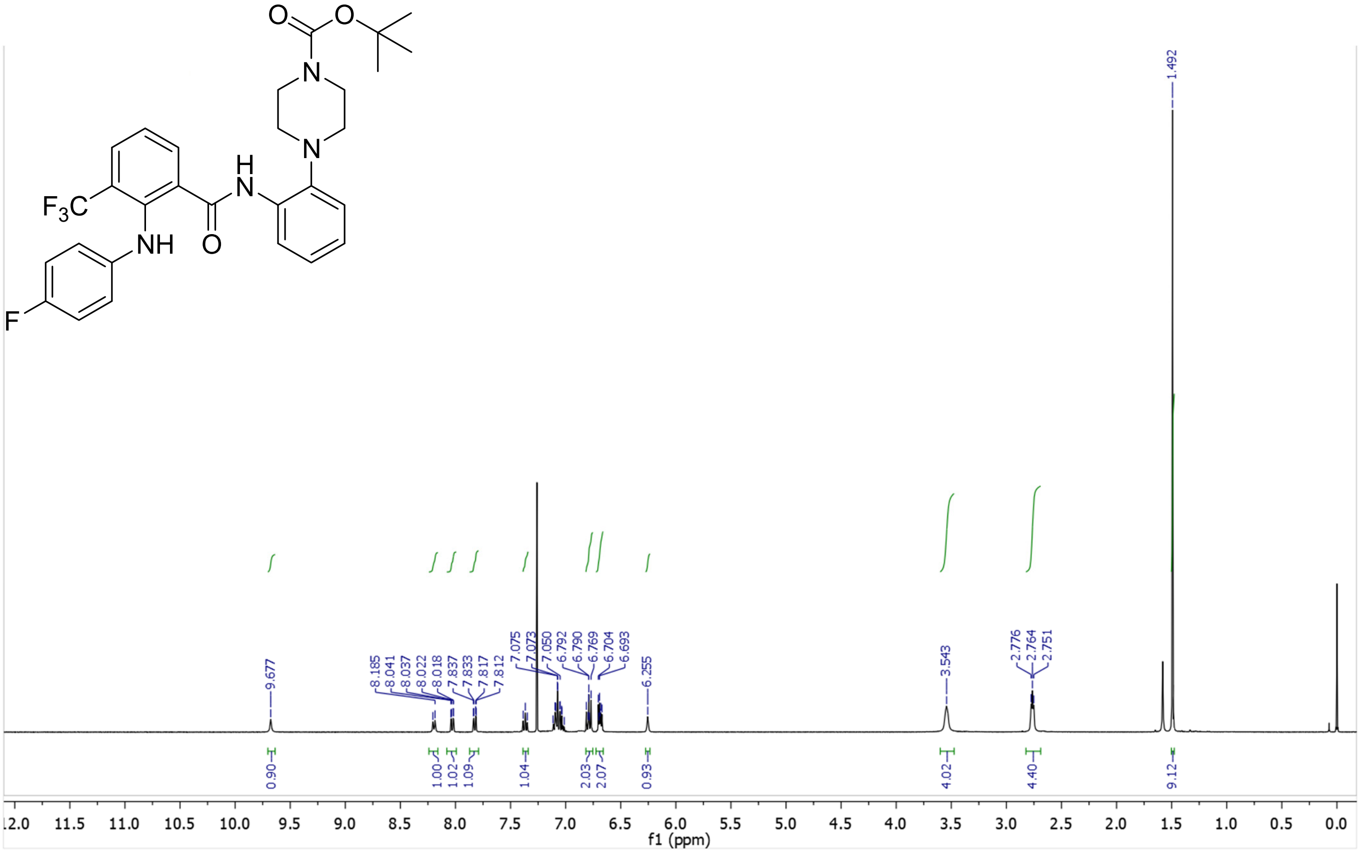

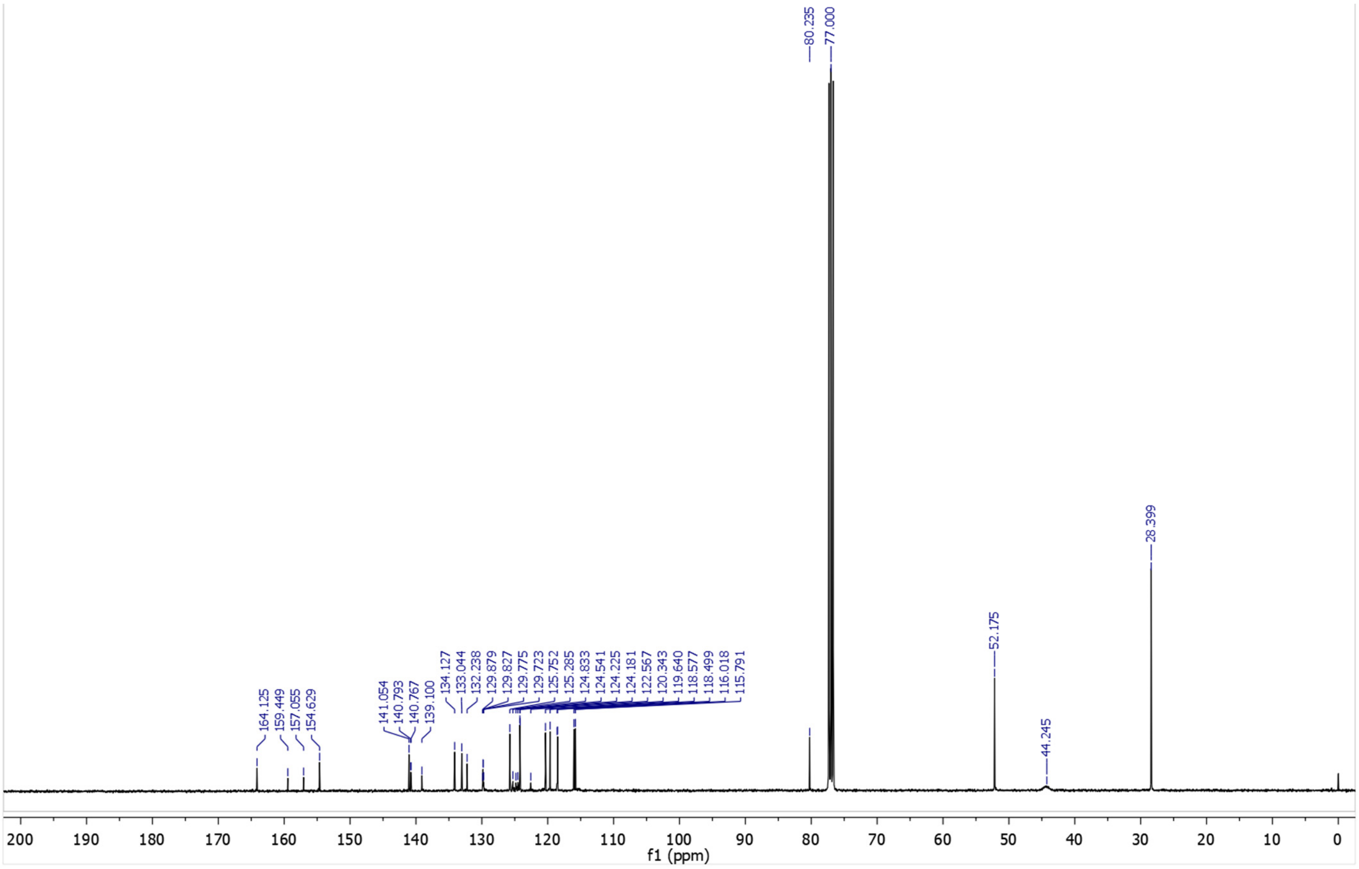

Figure S13. 1H NMR at 400 MHz and 13C NMR at 100 MHz spectra for compound **33** ($CDCl_3$)

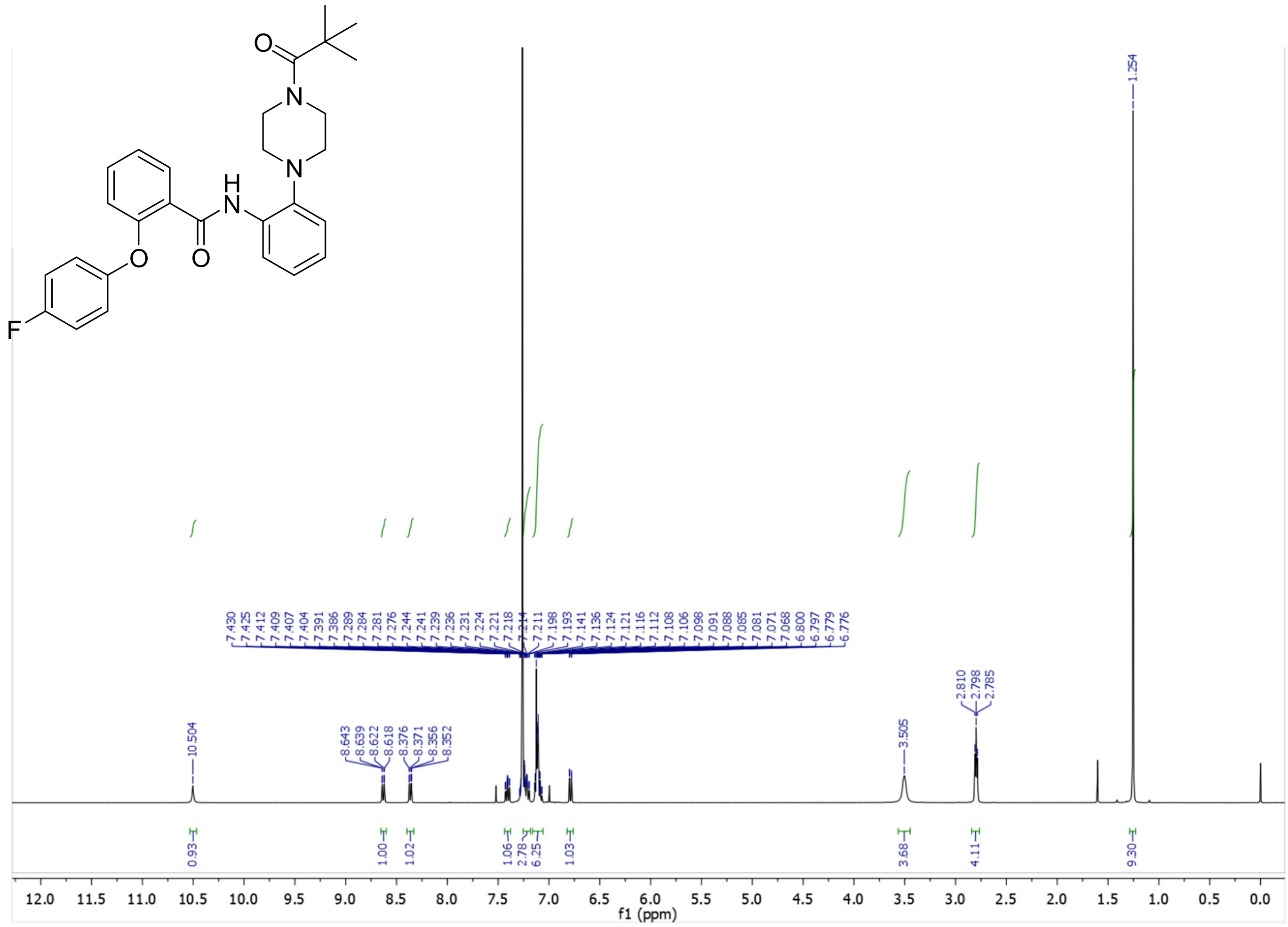

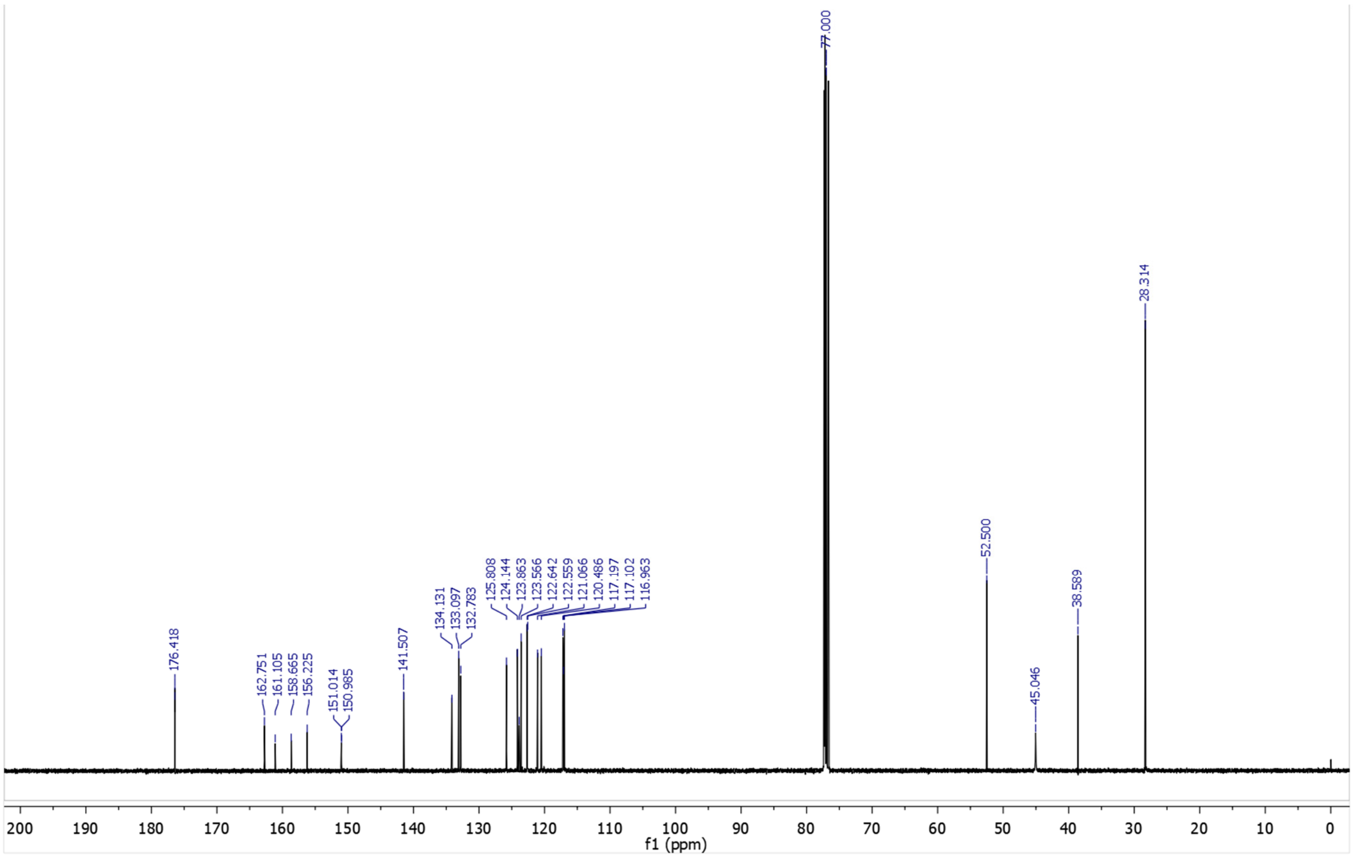

Figure S14. 1H NMR at 400 MHz and 13C NMR at 100 MHz spectra for compound **34** ($CDCl_3$)

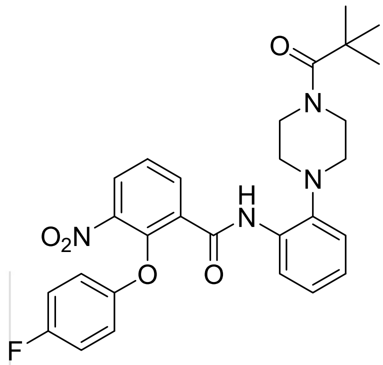

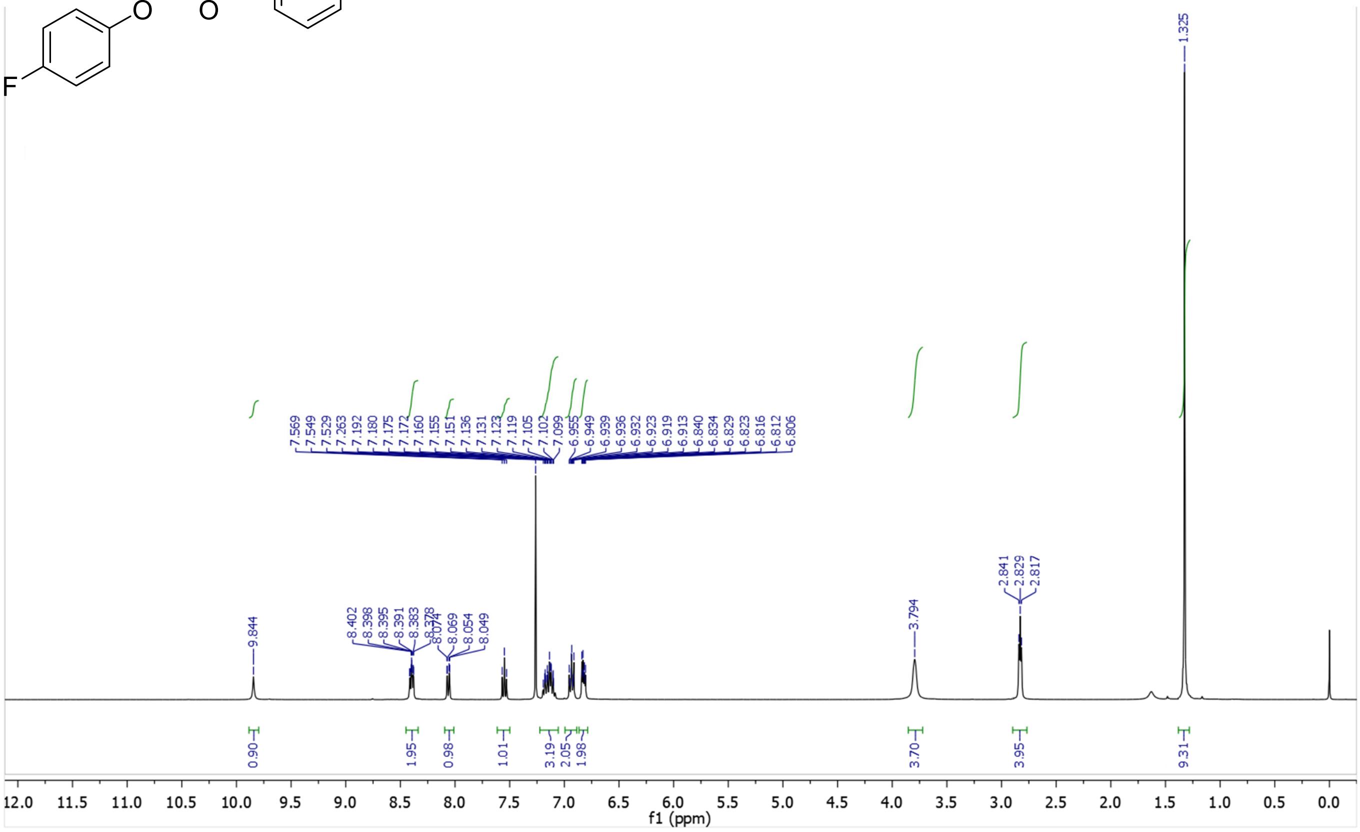

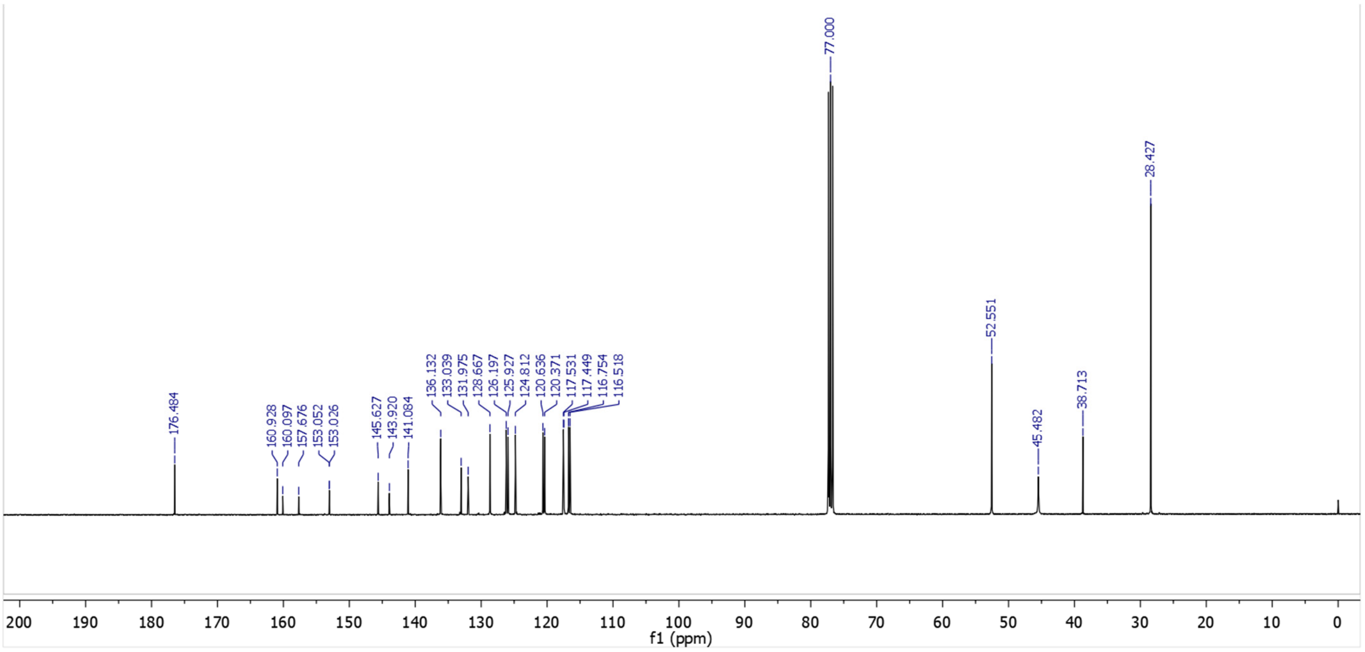

Figure S15. 1H NMR at 400 MHz and 13C NMR at 100 MHz spectra for compound **35** ($CDCl_3$)

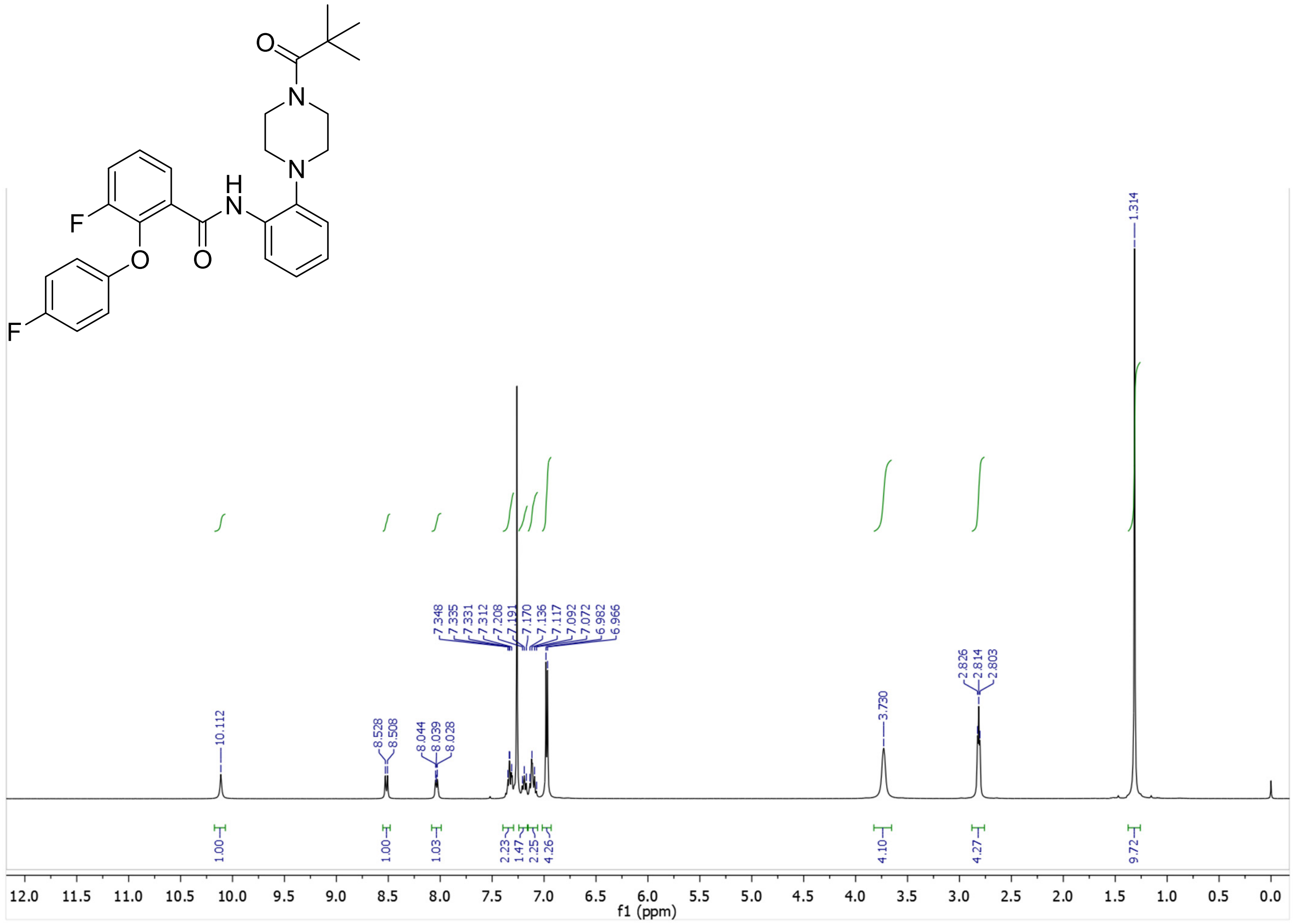

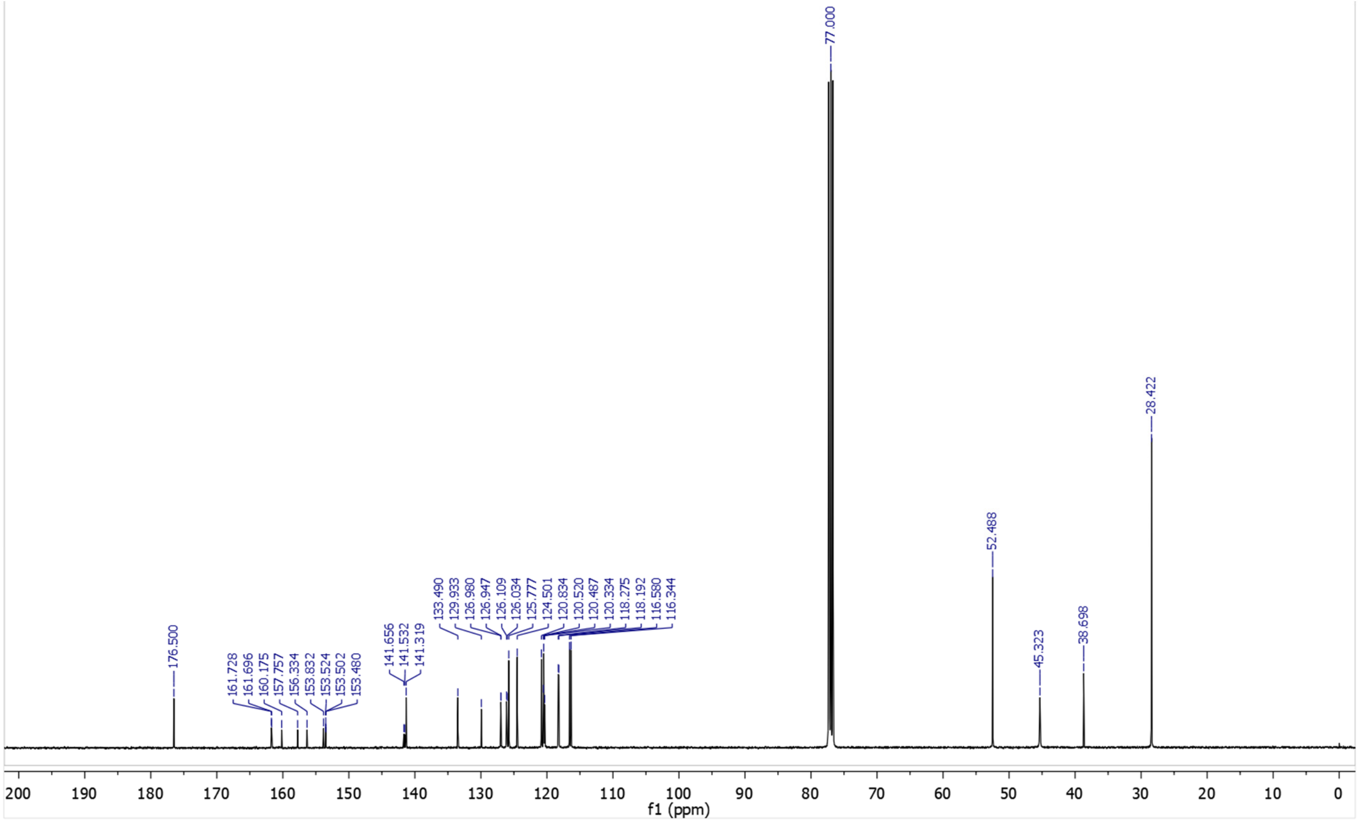

Figure S16. 1H NMR at 400 MHz and 13C NMR at 100 MHz spectra for compound **36** ($CDCl_3$)

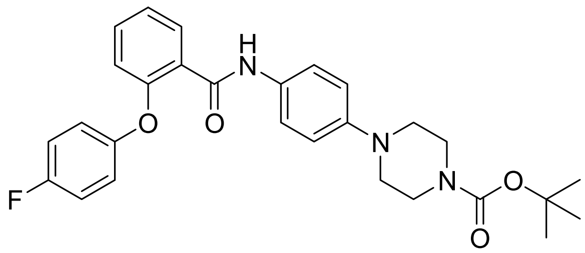

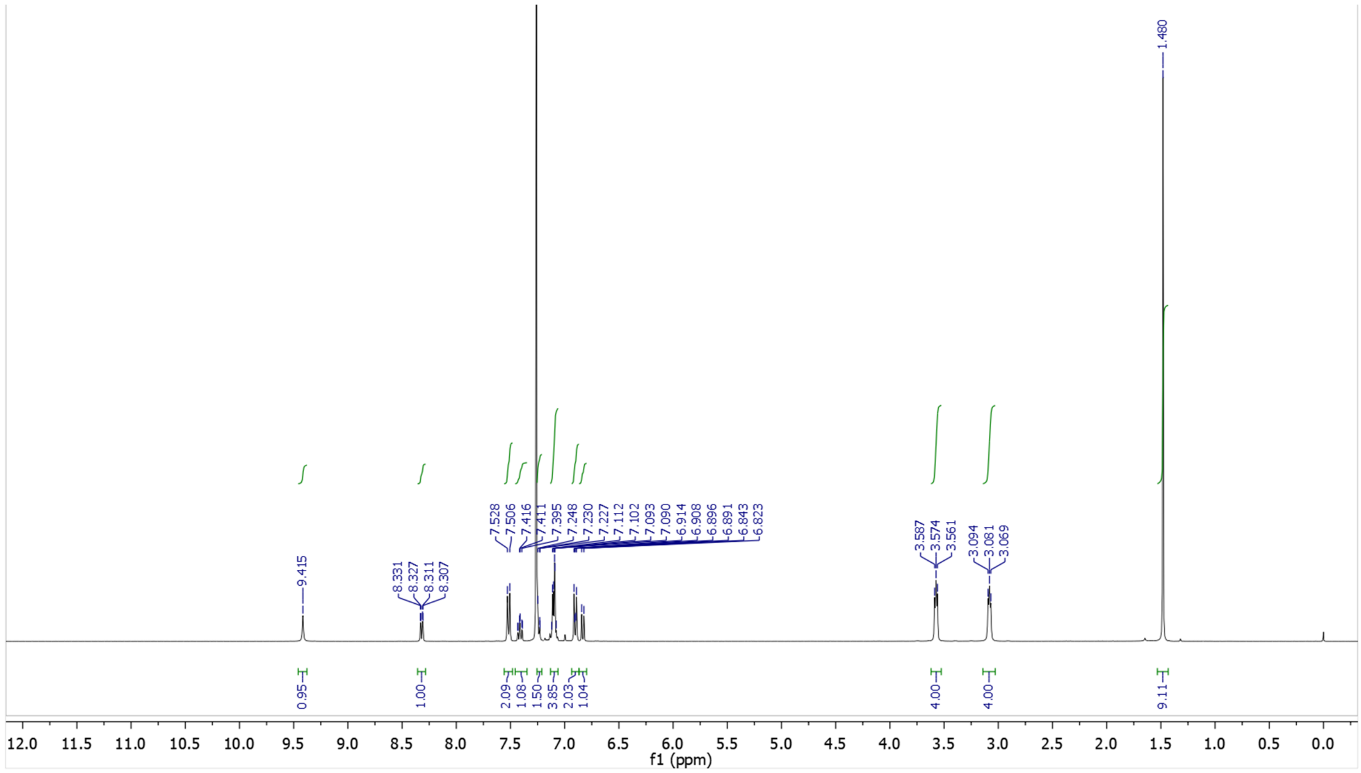

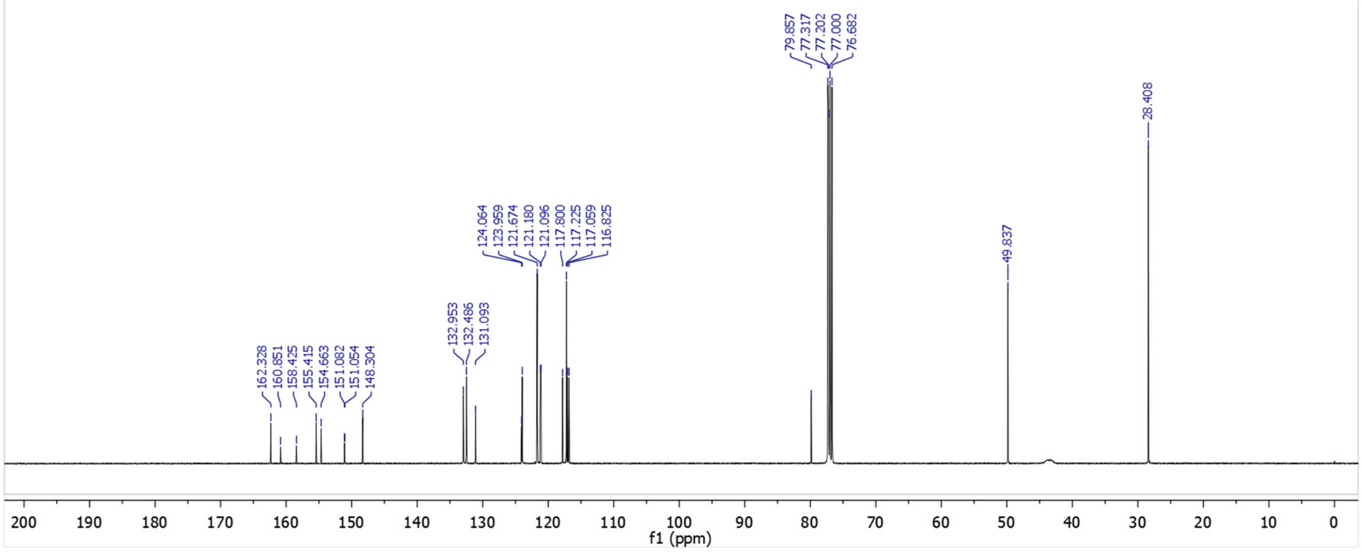

Figure S17. 1H NMR at 400 MHz and 13C NMR at 100 MHz spectra for compound **37** ($CDCl_3$)

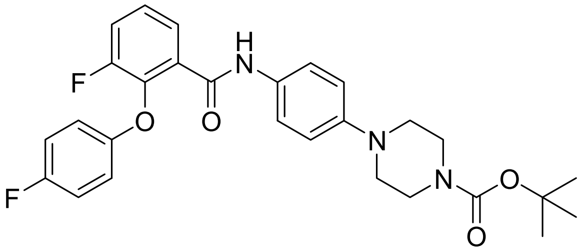

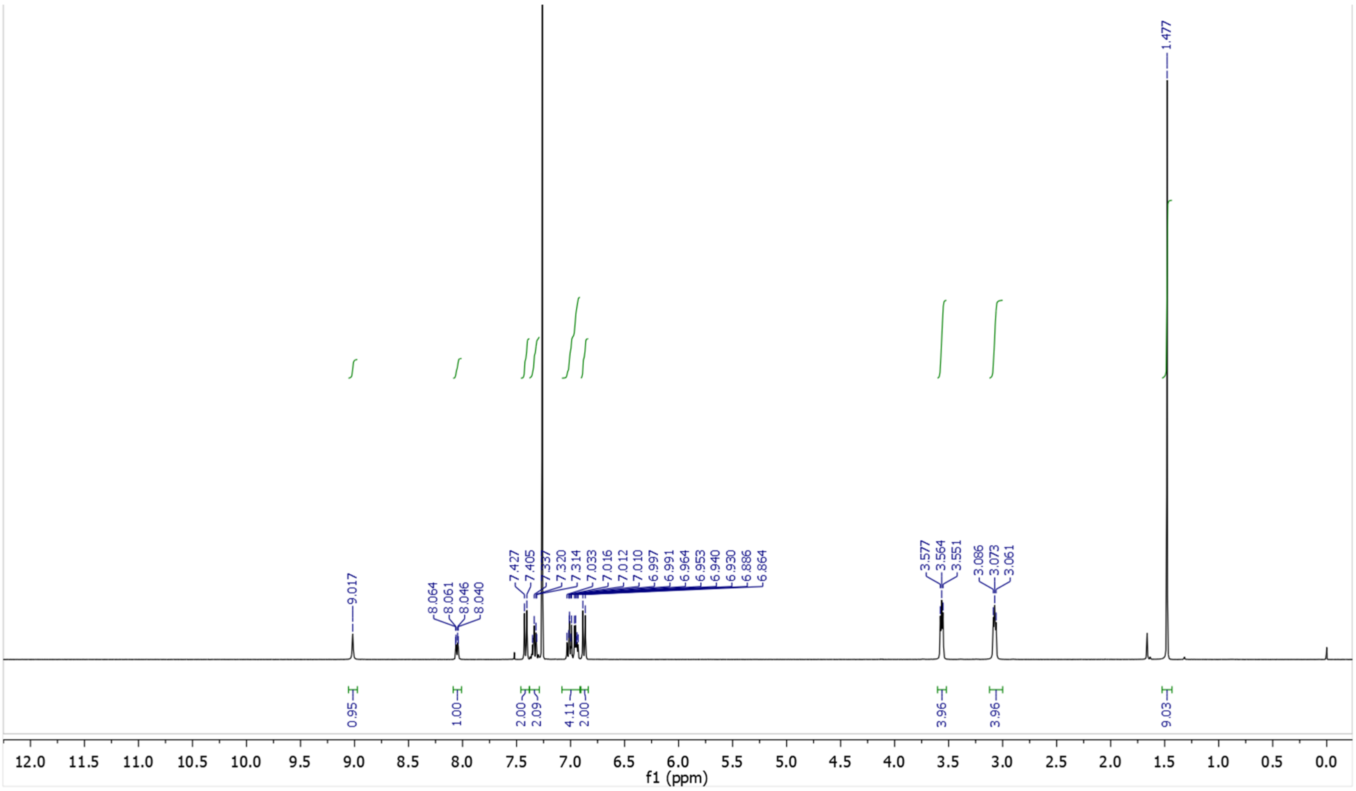

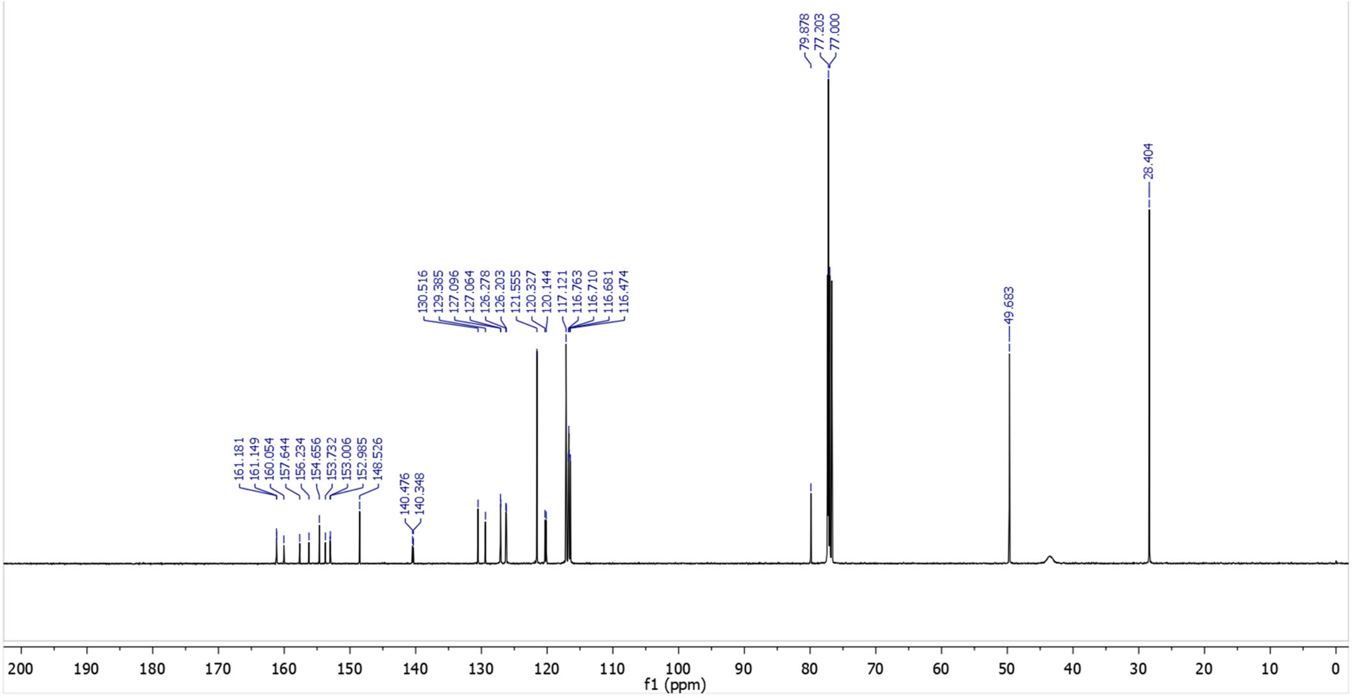

Figure S18. 1H NMR at 400 MHz and 13C NMR at 100 MHz spectra for compound **38** ($CDCl_3$)

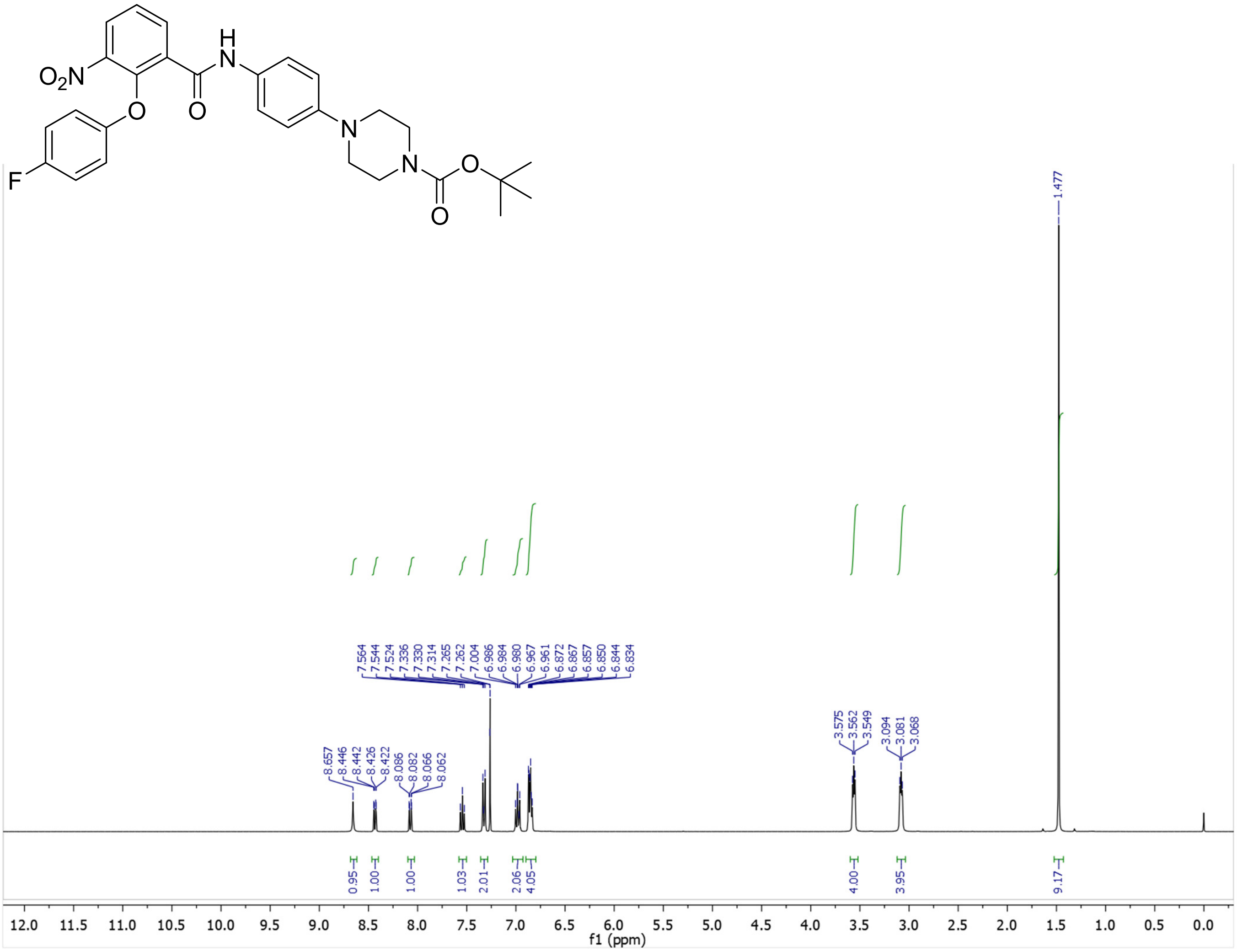

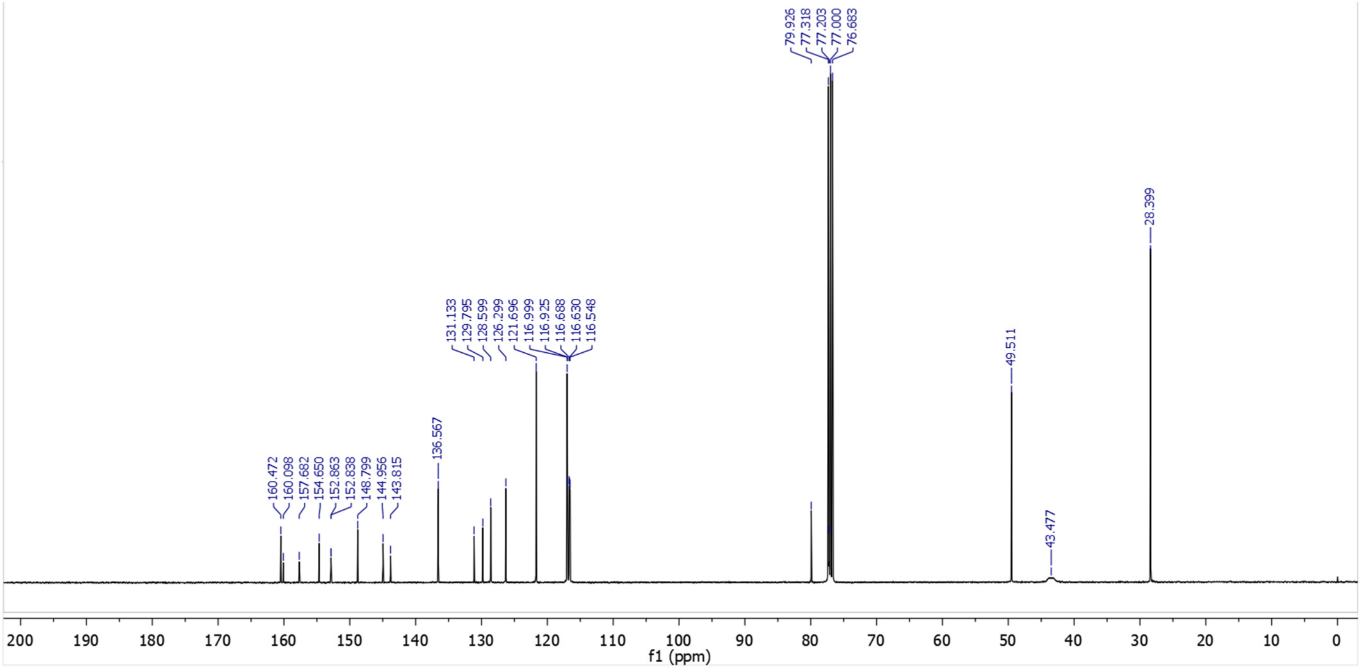

Figure S19. 1H NMR at 400 MHz and 13C NMR at 100 MHz spectra for compound **39** ($CDCl_3$)

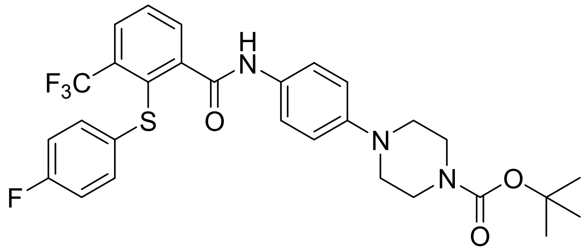

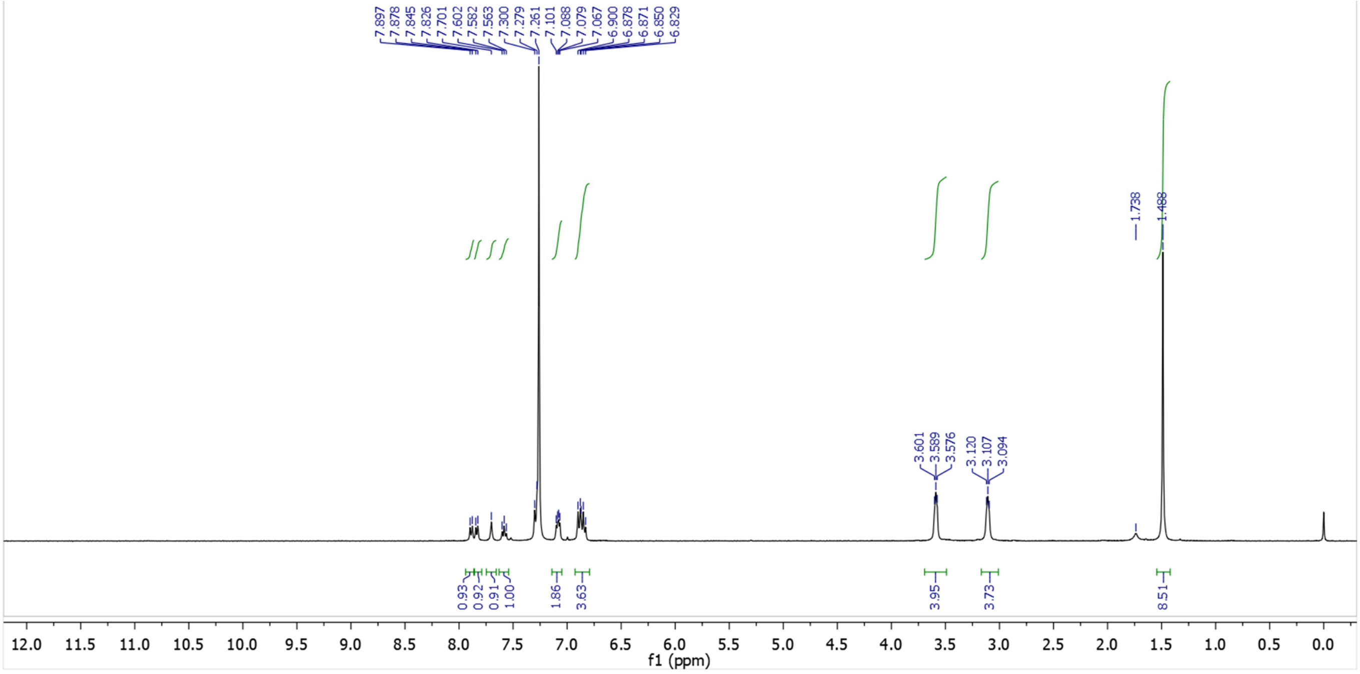

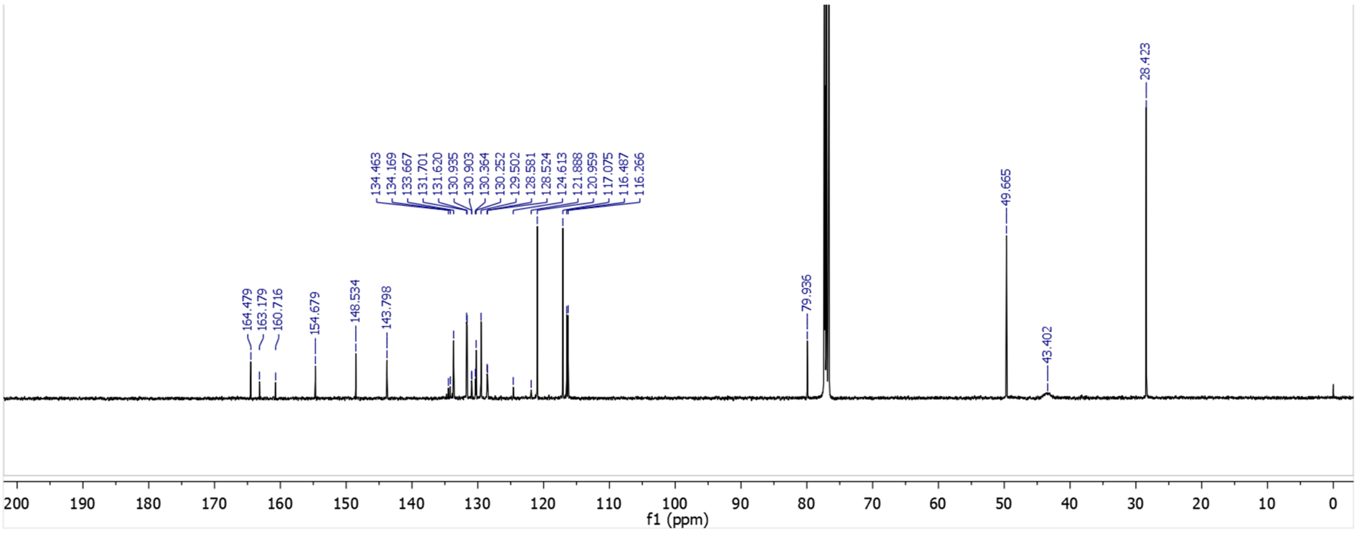

Figure S20. 1H NMR at 400 MHz and 13C NMR at 100 MHz spectra for compound **40** ($CDCl_3$)

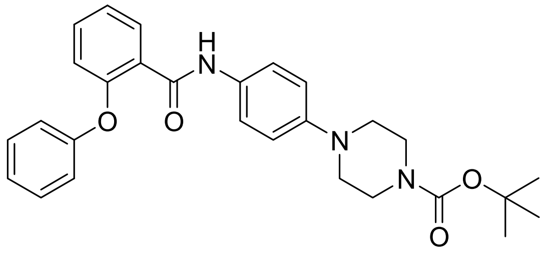

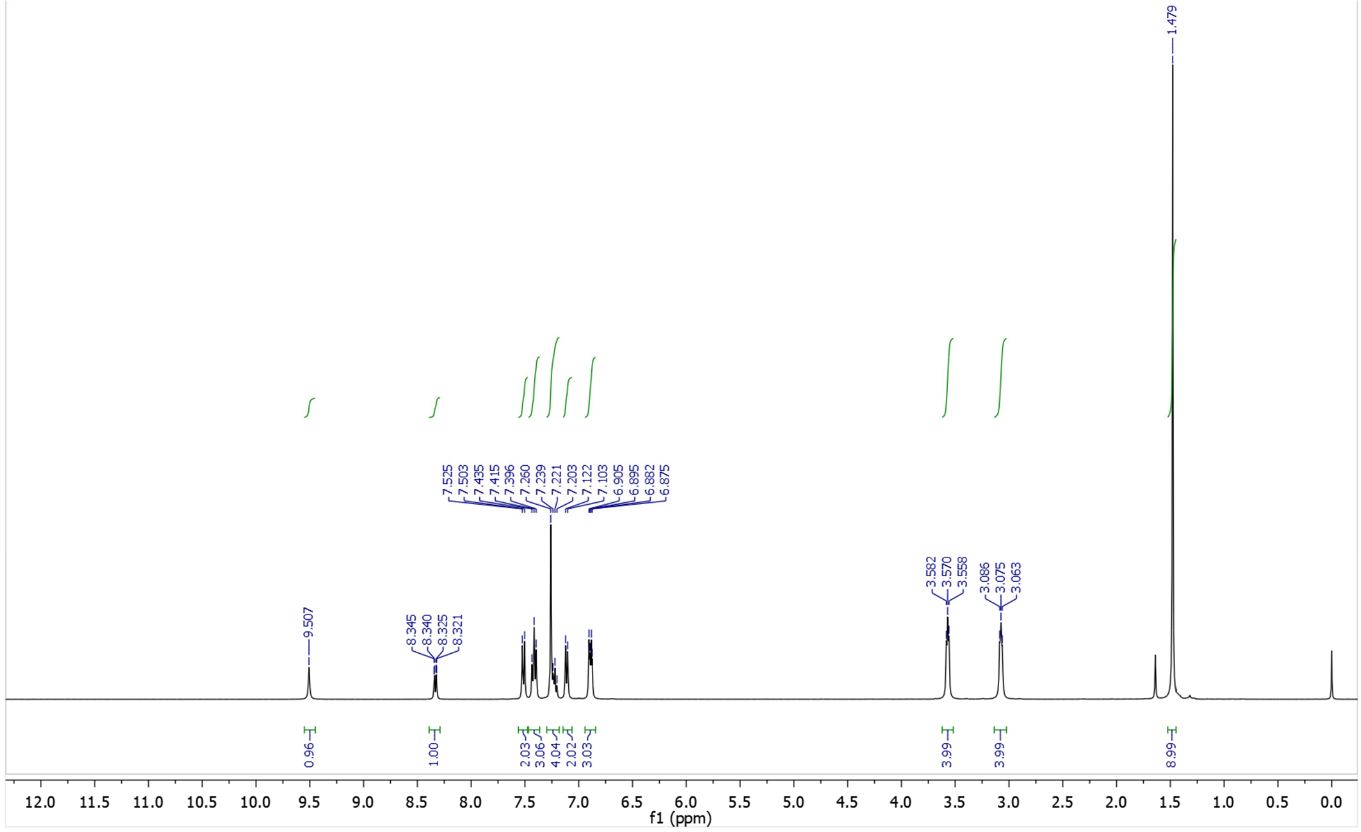

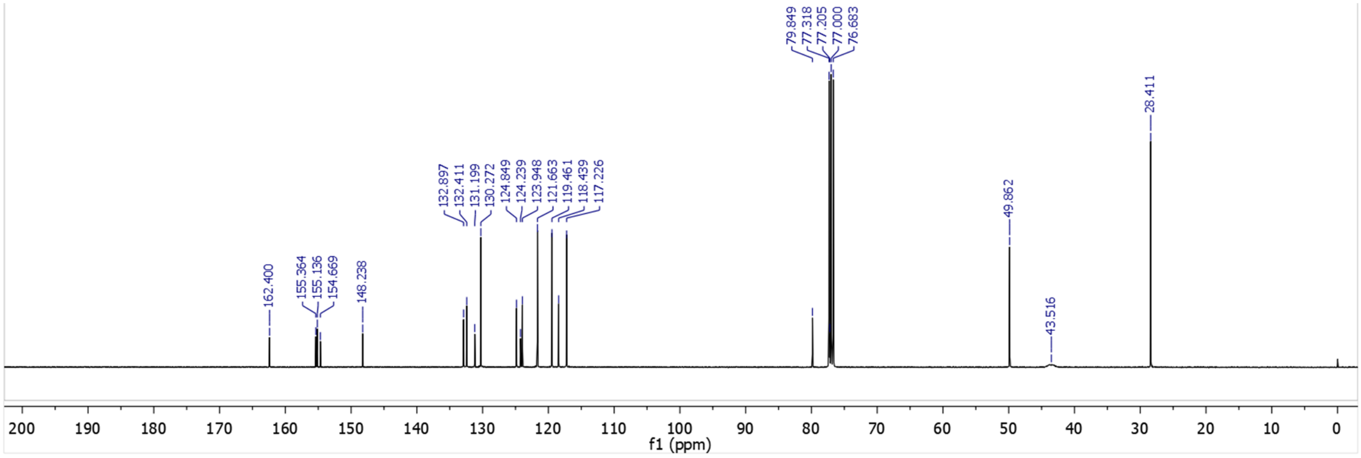

Figure S21. 1H NMR at 400 MHz and 13C NMR at 100 MHz spectra for compound **41** ($CDCl_3$)

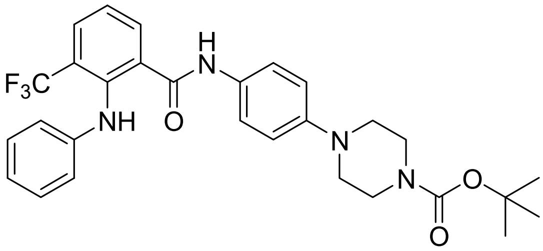

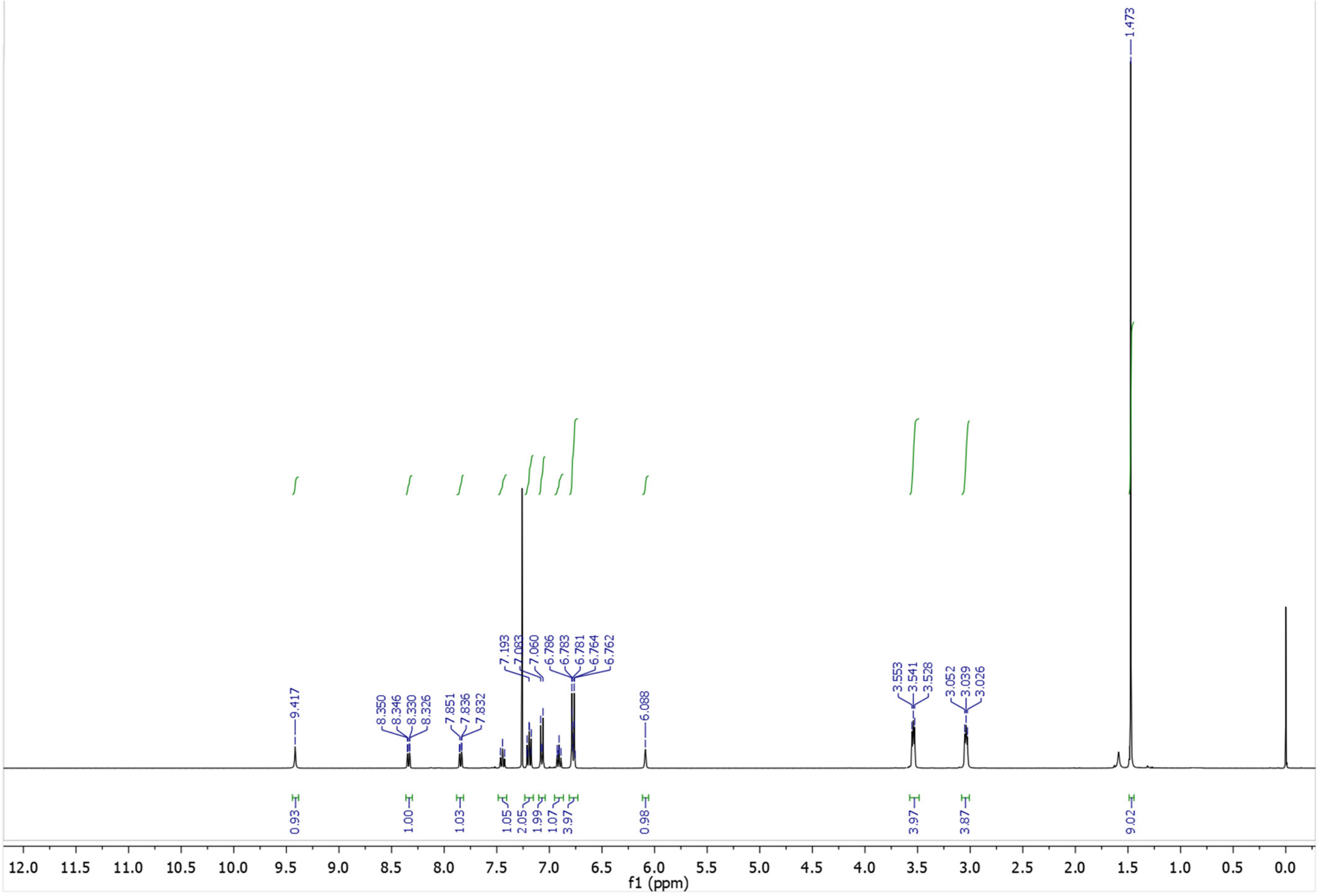

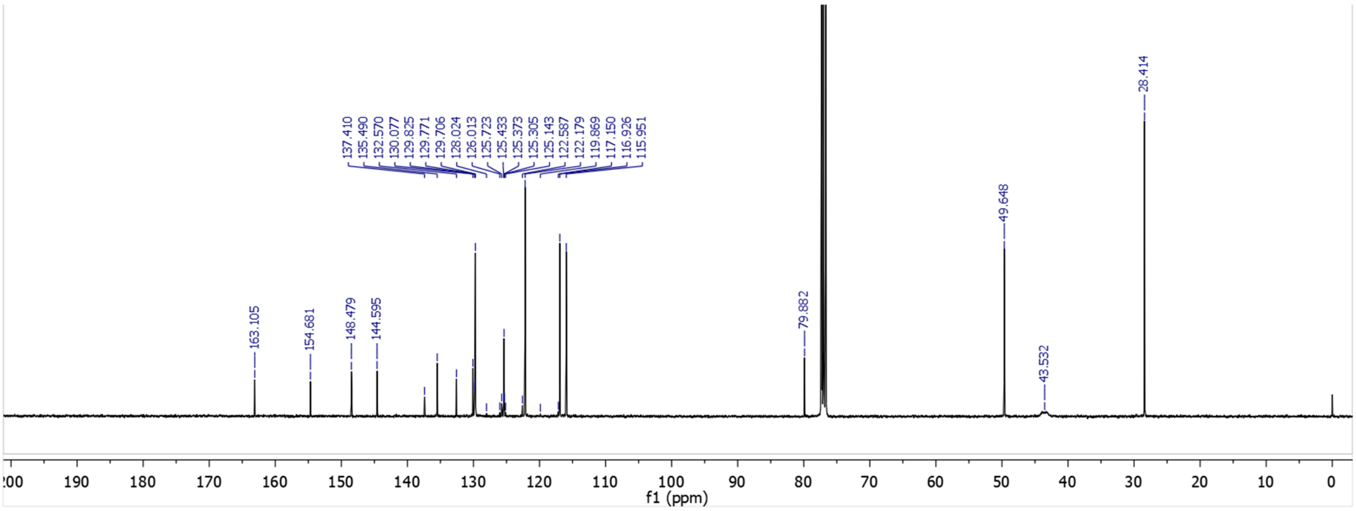

Figure S22. 1H NMR at 400 MHz and 13C NMR at 100 MHz spectra for compound **42** ($CDCl_3$)

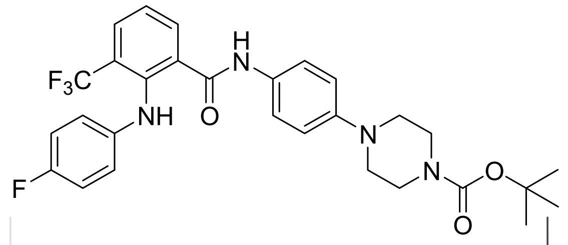

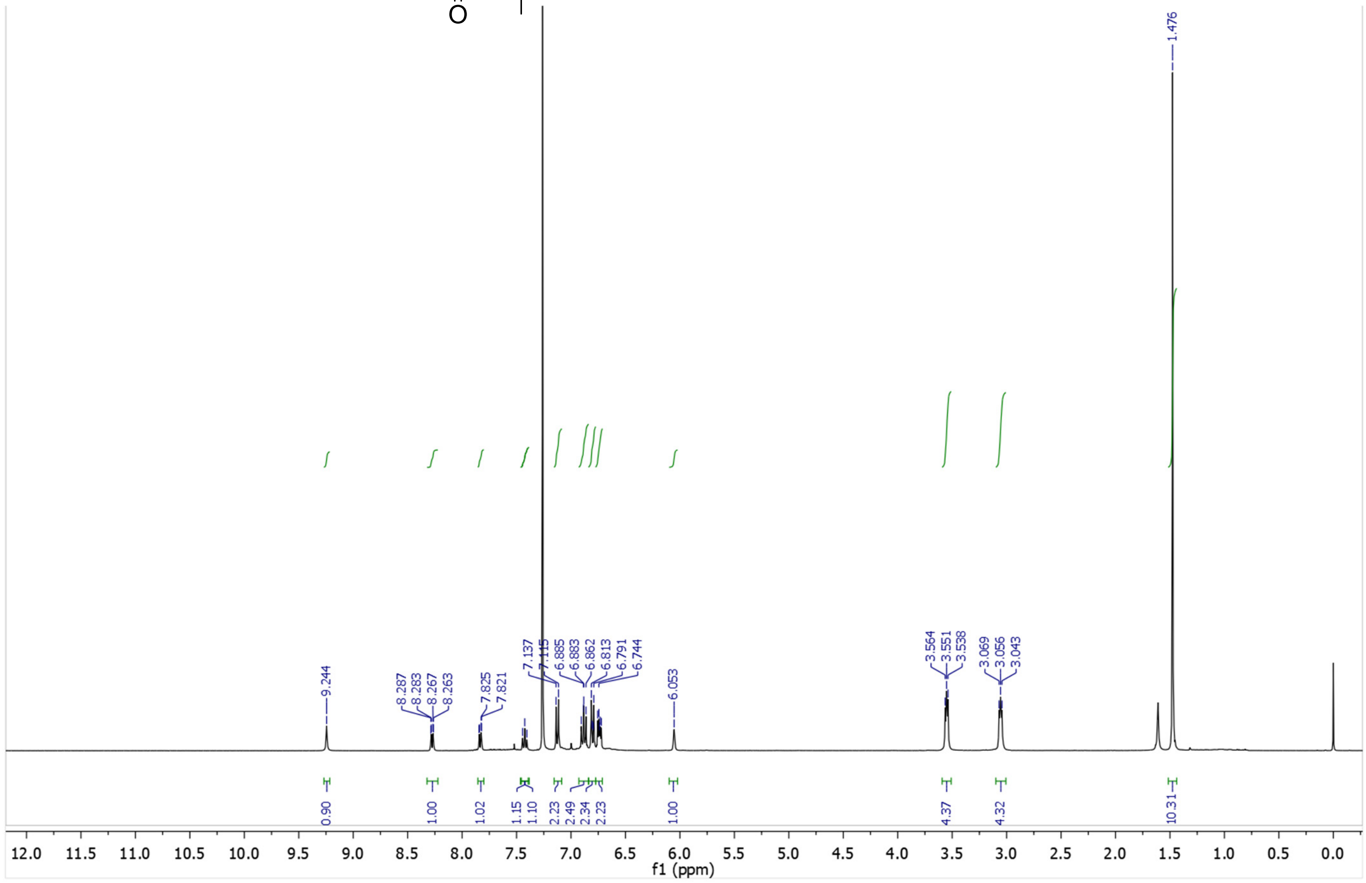

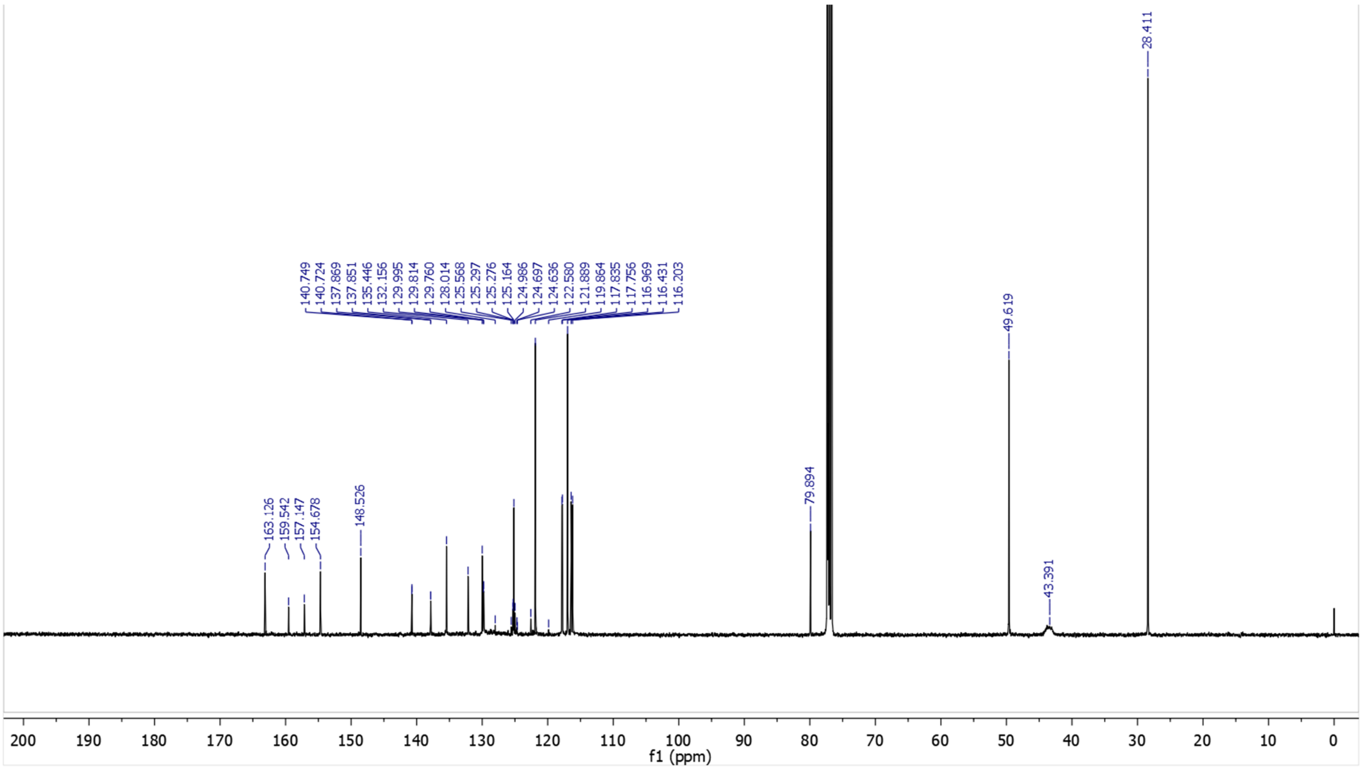

Figure S23. 1H NMR at 400 MHz and 13C NMR at 100 MHz spectra for compound **43** ($CDCl_3$)

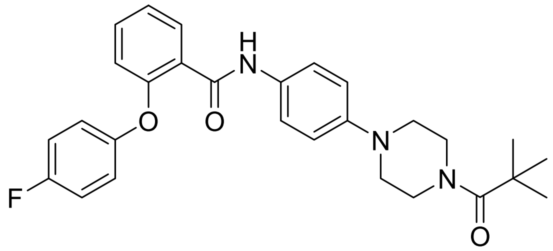

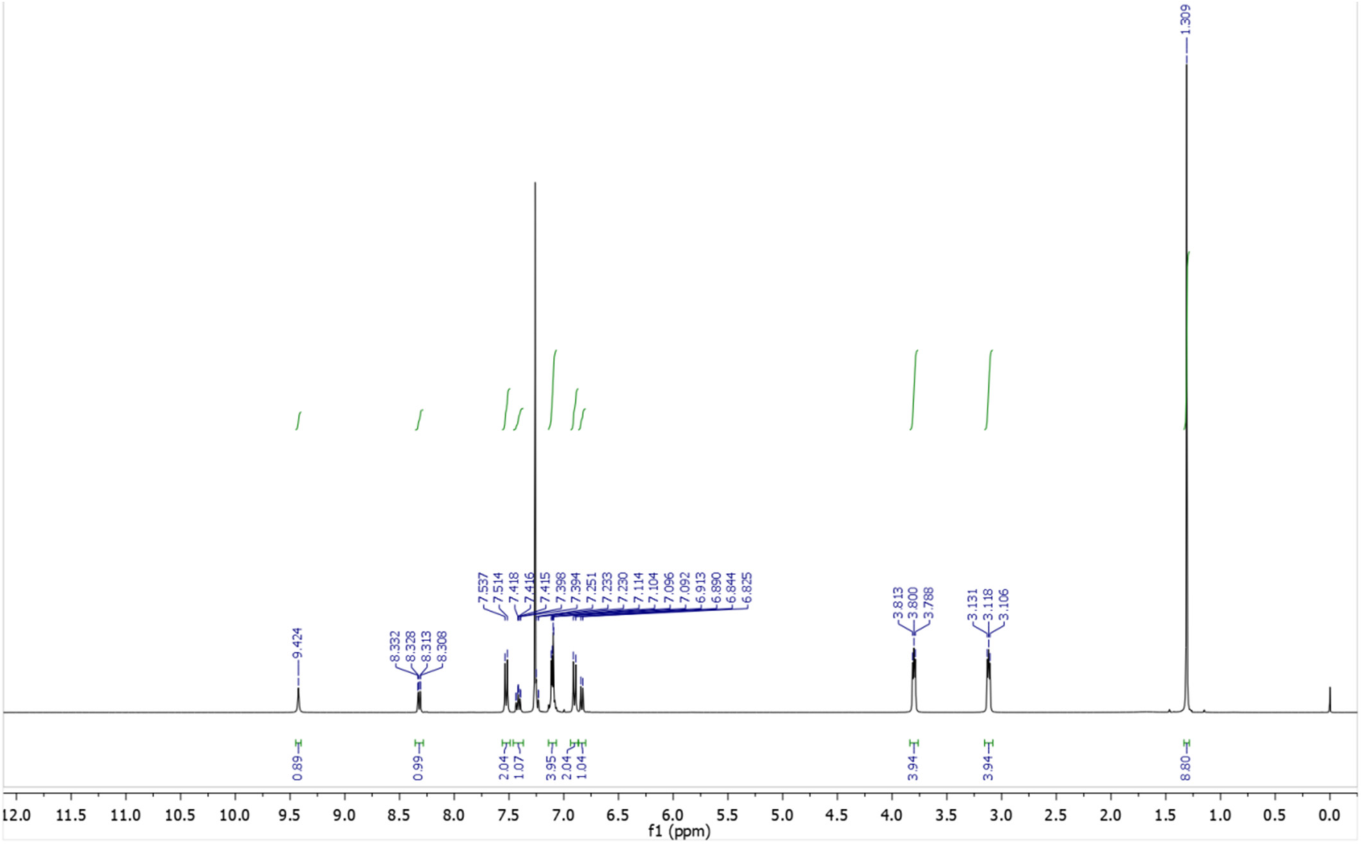

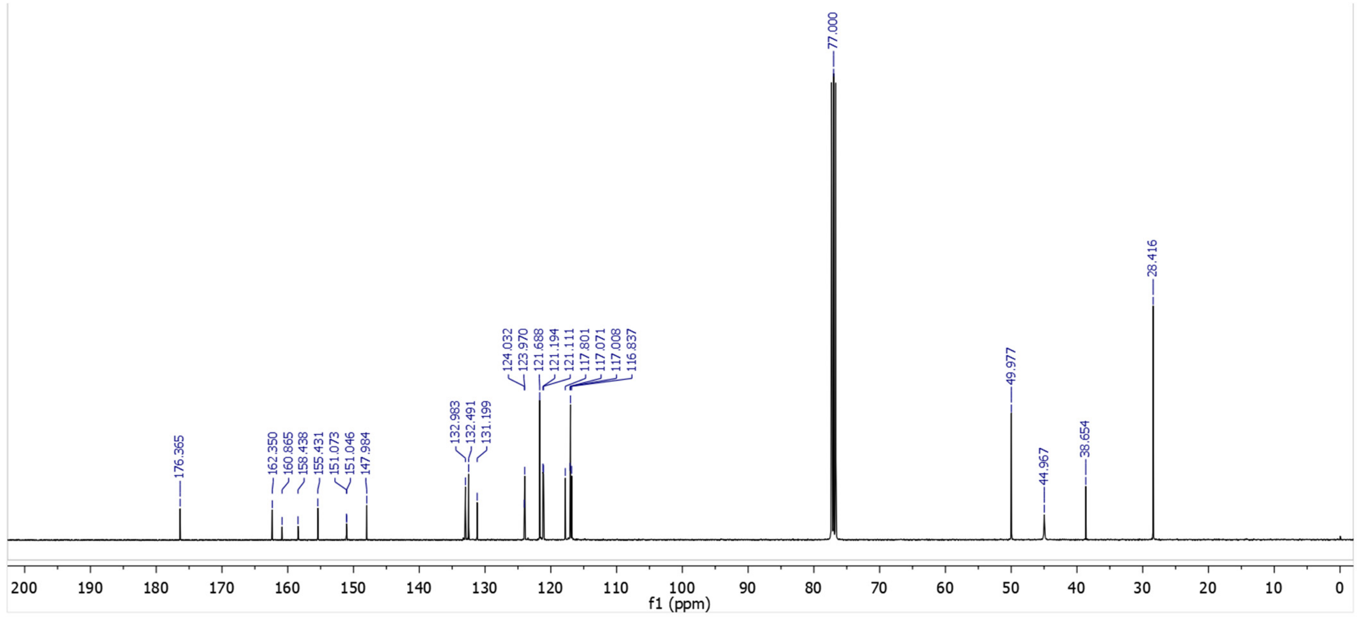

Figure S24. 1H NMR at 400 MHz and 13C NMR at 100 MHz spectra for compound **44** ($CDCl_3$)

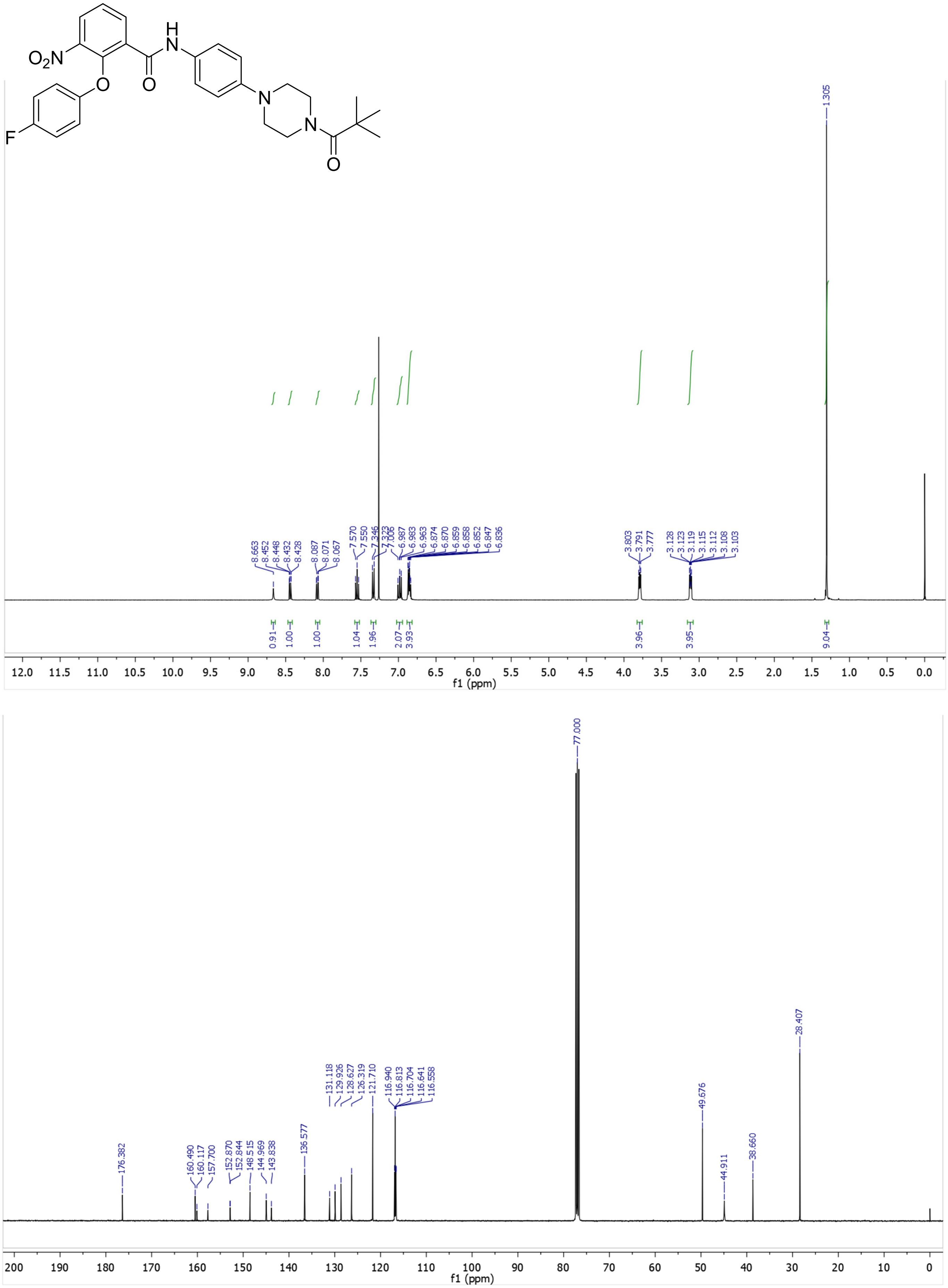

Figure S25. 1H NMR at 400 MHz and 13C NMR at 100 MHz spectra for compound **45** ($CDCl_3$)

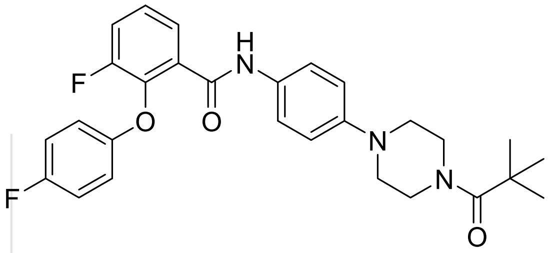

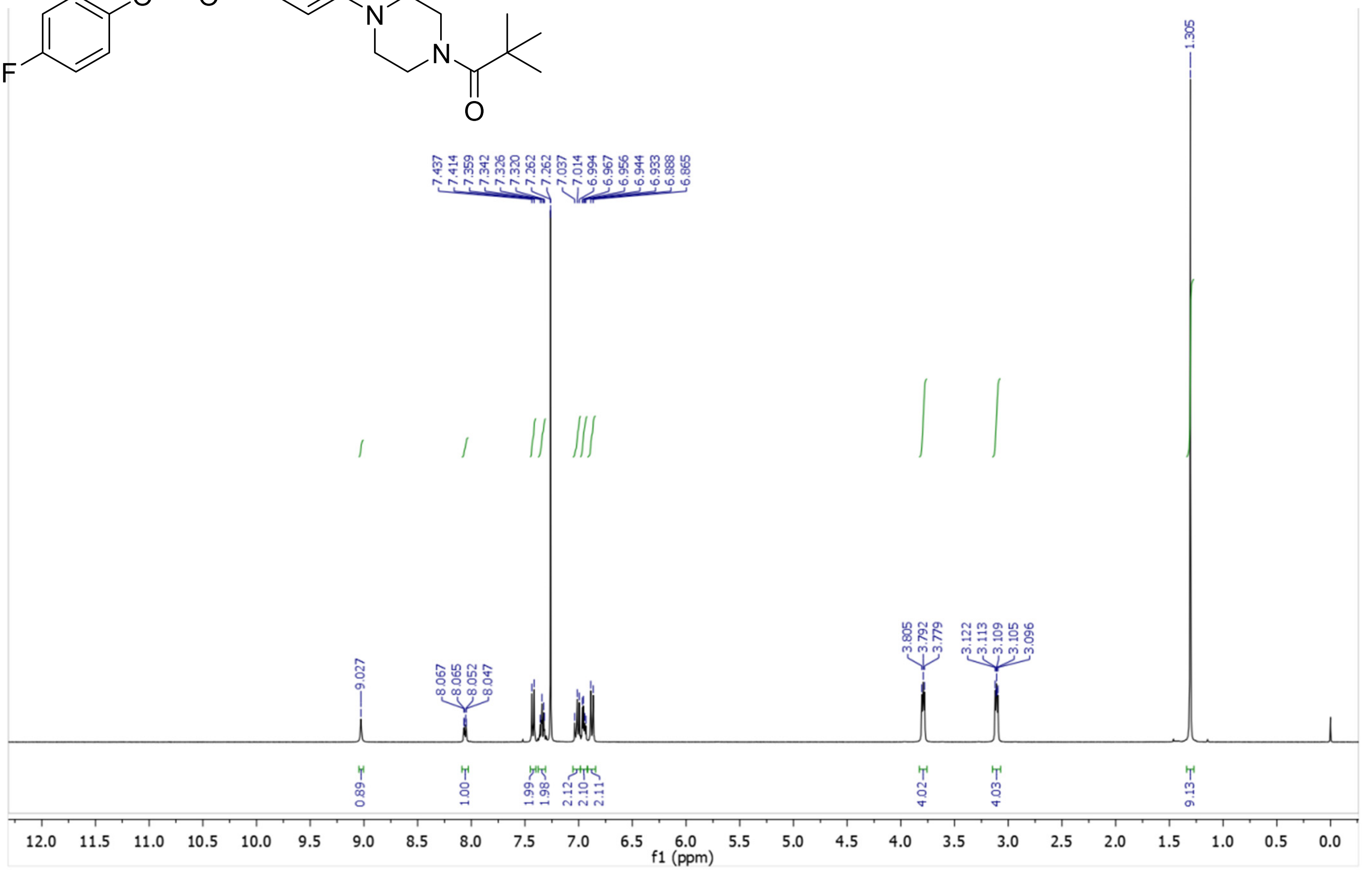

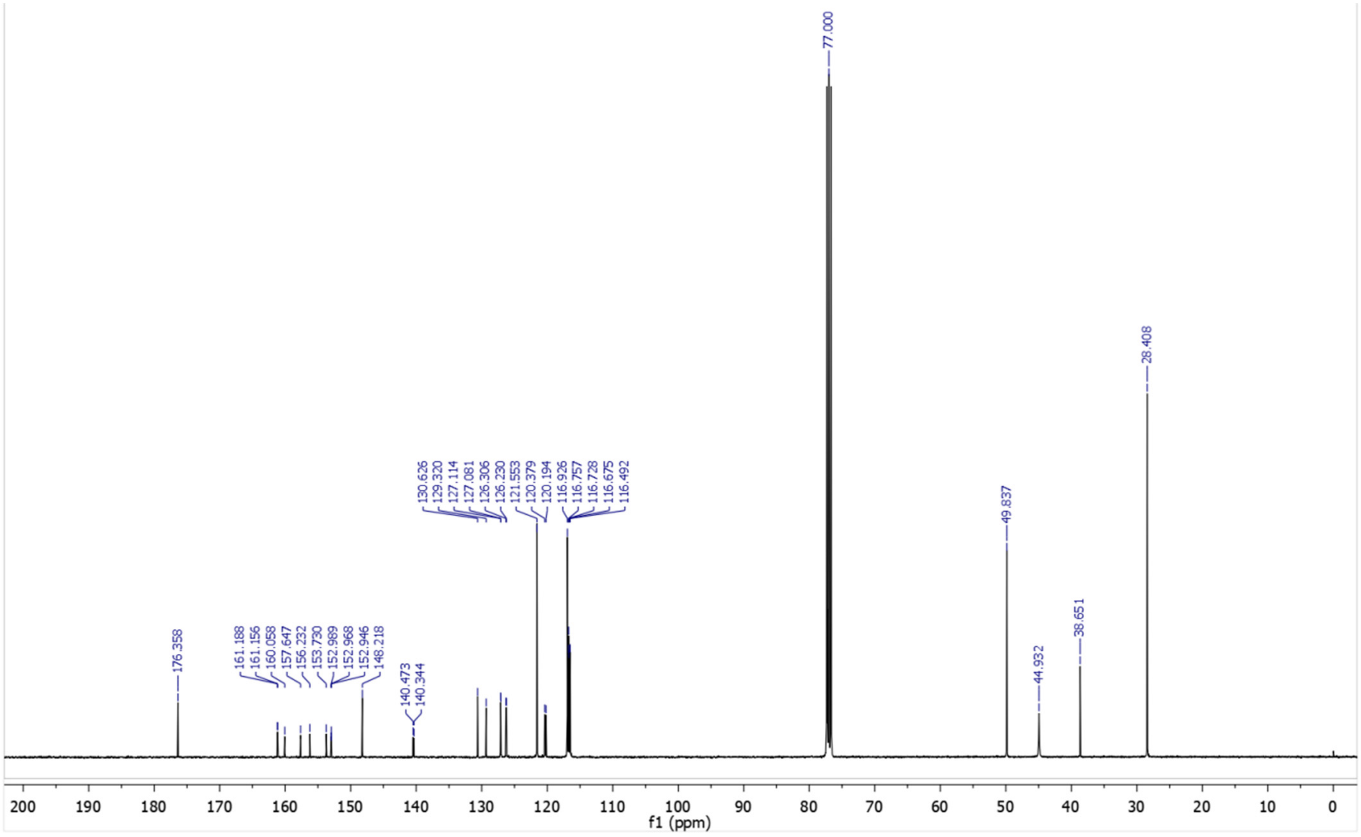

Figure S26. 1H NMR at 400 MHz and 13C NMR at 100 MHz spectra for compound **46** ($CDCl_3$)

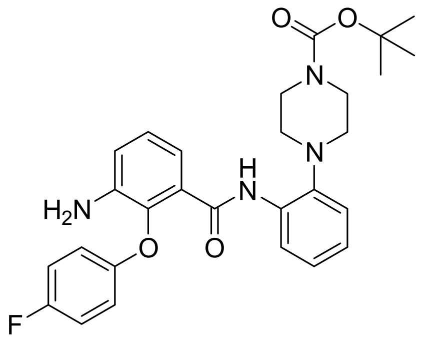

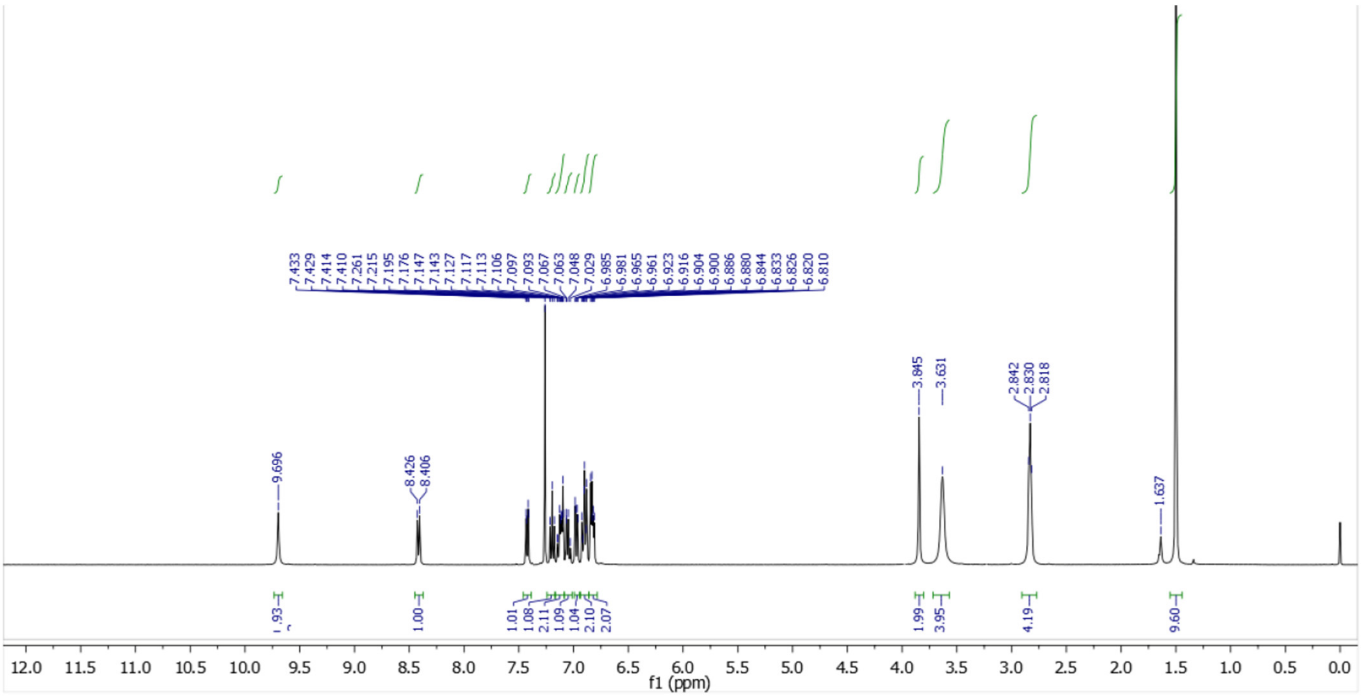

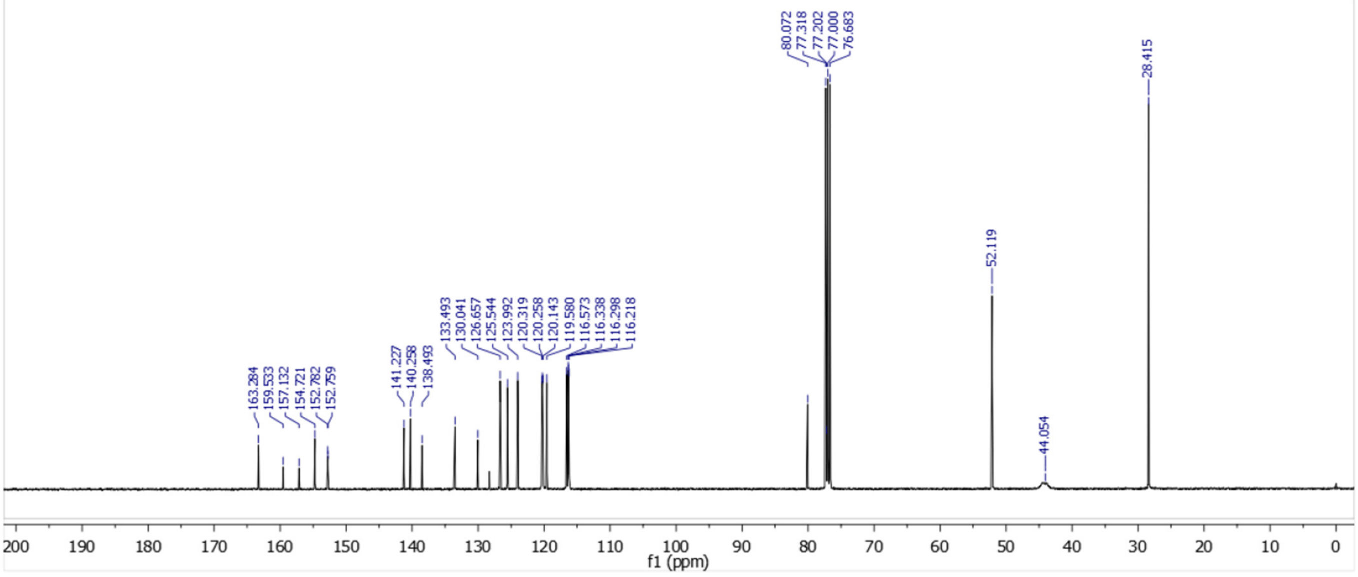

Figure S27. 1H NMR at 400 MHz and 13C NMR at 100 MHz spectra for compound **47** ($CDCl_3$)

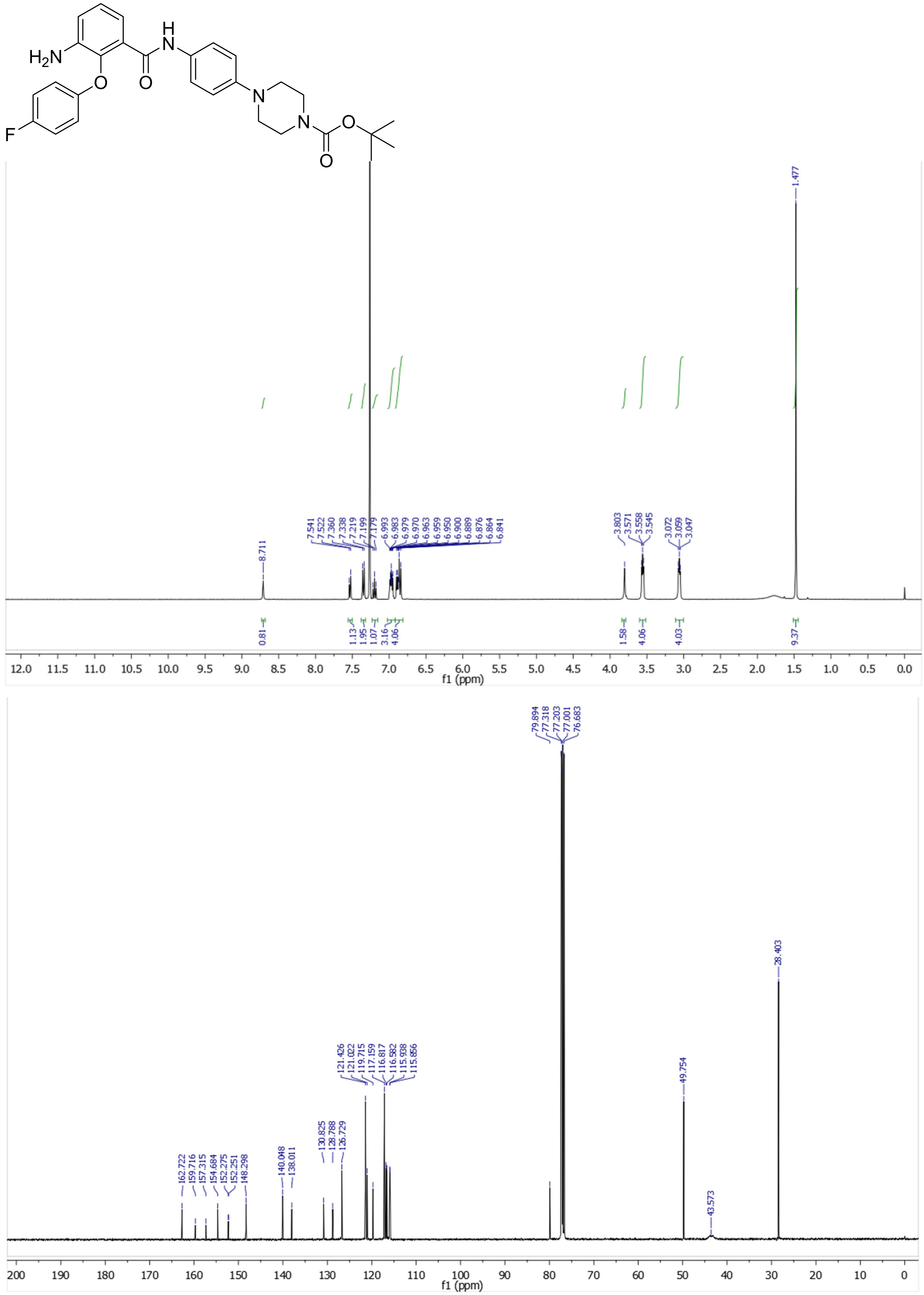

Supplement: Supplementary file 1 [file pharmaceuticals-15-01503-s001.zip › pharmaceuticals-2052533-supplementary.pdf]
